# Access to 2-oxoazetidine-3-carboxylic acid derivatives via thermal microwave-assisted Wolff rearrangement of 3-diazotetramic acids in the presence of nucleophiles

**DOI:** 10.3762/bjoc.20.164

**Published:** 2024-08-05

**Authors:** Ivan Lyutin, Vasilisa Krivovicheva, Grigory Kantin, Dmitry Dar’in

**Affiliations:** 1 Institute of Chemistry, Saint Petersburg State University, 26 Universitetskiy pr., Peterhof, Saint Petersburg 198504, Russian Federation; 2 Saint Petersburg Research Institute of Phthisiopulmonology, 2-4 Ligovsky pr., Saint Petersburg 191036, Russian Federation

**Keywords:** β-lactams, diazotetramic acids, nucleophiles, spirocycles, thermolysis, Wolff rearrangement

## Abstract

In this work, we report an efficient approach to 2-oxoazetidine-3-carboxylic acid derivatives based on a thermally promoted Wolff rearrangement of diazotetramic acids in the presence of nucleophiles. The method allows easy variation of the substituent in the exocyclic acyl group by introducing different *N*-, *O*-, and *S*-nucleophilic reagents into the reaction. The reaction of chiral diazotetramic acids leads exclusively to *trans*-diastereomeric β-lactams. The use of variously substituted diazotetramic acids, including spirocyclic derivatives, as well as a wide range of nucleophiles provides access to a structural diversity of medically relevant 2-oxoazetidine-3-carboxylic acid amides and esters.

## Introduction

The importance of the β-lactam (azetidin-2-one) scaffold to medicinal chemistry and drug design is self-evident. This four-membered heterocycle is a key fragment of many antibiotics [1], including penicillin and its analogues, as well as other pharmacologically important molecules [2]. Therefore, the search for new efficient and versatile methods for the preparation of structurally diverse β-lactam derivatives is of great importance and relevance.

**Scheme 1:**
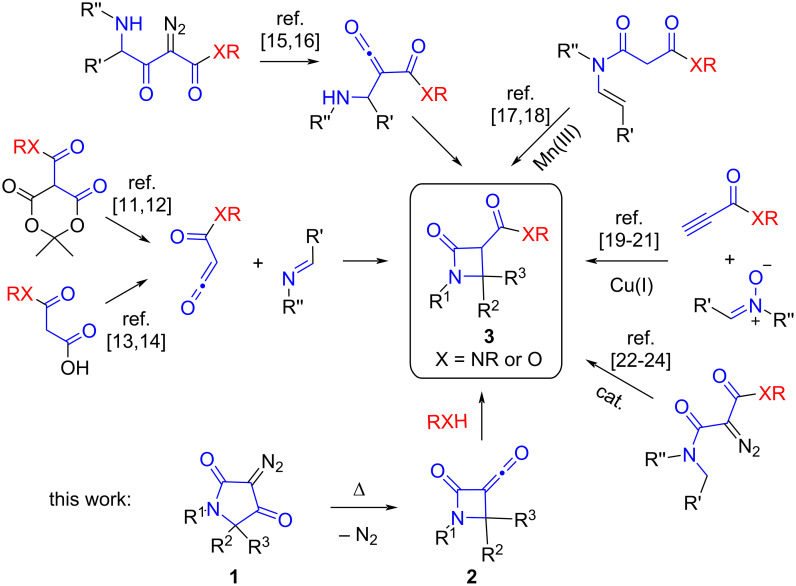
Synthetic routes to 2-oxoazetidine-3-carboxylic acid derivatives.

Continuing the investigation of the reactivity and synthetic potential of diazotetramic acids (**1**), we have recently shown that these diazo reagents can act as precursors of β-lactam ketenes **2** generated by a thermally promoted Wolff rearrangement [3]. The interaction of such ketenes with nucleophiles of different nature could serve as a source of libraries of structurally diverse 2-oxoazetidine-3-carboxylic acid derivatives **3** (Scheme 1).

The 2-oxoazetidine-3-carboxylic acid derivatives (mainly amides) exhibit various types of biological activity, among which the following can be highlighted: inhibition of β-lactamases [4–5], antitubercular properties [6], antiproliferative and antibacterial activity [7], herbicidal properties [8–9], inhibition of neutral amino acid transporter (SLC6A19) [10]. Hence, developing new synthetic methods to create structurally diverse 2-oxoazetidine-3-carboxylic acid derivatives is a highly valuable endeavour that could have a positive impact on future drug discovery.

Most synthetic approaches to amides and esters of 2-oxoazetidine-3-carboxylic acids reported in the literature are based on the construction of the β-lactam ring (Scheme 1). The main methods include the [2 + 2] cycloaddition of acyl ketenes, generated by various methods, with imines [11–14] and the Wolff rearrangement of γ-amino-α-diazo-β-ketoesters followed by intramolecular cyclization [15–16]. Additionally, the manganese(III)-promoted cyclization of *N*-alkenyl malonamides [17–18] and the Cu(I)-catalyzed reaction of propiolic acid derivatives with nitrones (Kinugasa reaction) [19–21] should also be mentioned, as well as intramolecular C–H insertion using diazomonomalonamides under the action of various catalysts which is a very efficient method for preparing β-lactam esters [22–25].

At the same time, from the point of view of easy variation of the substituent in the exocyclic acyl group (RX), a method allowing the introduction of this moiety at the last step of the synthesis would be of great demand. We proposed that, besides modifying the 2-oxoazetidine-3-carboxylic acids themselves, such an approach could involve diazotetramic acids, subjected to thermal Wolff rearrangement, with various nucleophiles.

The application of the Wolff rearrangement in organic synthesis as a route to generate ketenes is being actively investigated, involving both acyclic and carbocyclic diazocarbonyl compounds [26]. At the same time, the use of diazoheterocyclic reagents (including diazotetramic acids) in this transformation, with the formation of heterocyclic ring contraction products, is represented in the literature only by isolated examples [27–31]. In addition, photoinitiation is mainly used, while the possibilities of thermolysis remain virtually unexplored.

Herein, we report our findings obtained while investigating a synthetic approach to 2-oxoazetidine-3-carboxylic acid derivatives based on the thermally promoted Wolff rearrangement of diazotetramic acids.

## Results and Discussion

Diazotetramic acid derivatives **1** are available in a wide variety using the techniques described previously [32]. The conditions for their thermal decomposition were tested in a previous study [3]. The reaction requires rather severe heating under microwave irradiation (200 °C, chlorobenzene, sealed vial), ensuring complete conversion of the diazo compound in a rather short time.

**Scheme 2:**
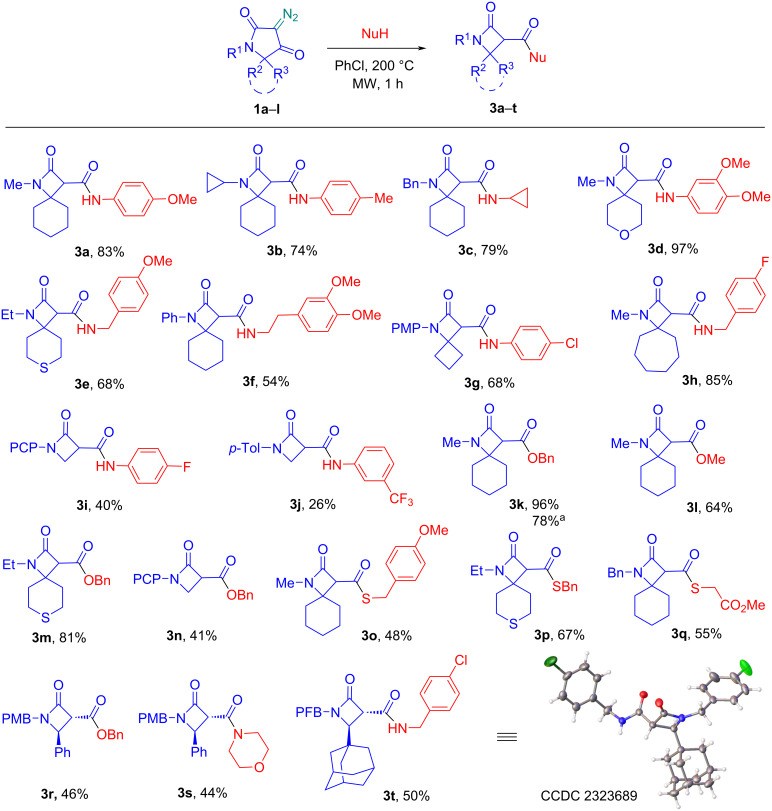
Scope of diazotetramic acids **1** thermolysis in the presence of various nucleophiles. PMP = *p*-methoxyphenyl, PCP = *p*-chlorophenyl, PMB = *p*-methoxybenzyl, PFB = *p*-fluorobenzyl; reaction scale – 0.25 mmol; ^a^scaled-up (1.5 mmol) yield.

Initial experiments using *p*-anisidine as a nucleophile showed that the target β-lactam derivative **3a** could be obtained in high yield (83%) after simple chromatographic separation of the reaction mixture (Scheme 2). When the synthesis was carried out using conventional heating in 1,2-dichlorobenzene (200 °C, 1 h), product **3a** was obtained in slightly lower yield (75%), so further experiments were carried out using microwave activation. We then introduced various aromatic and aliphatic amines as well as alcohols and mercaptans into the reaction. In order to demonstrate the structural diversity of the compounds obtained, a wide range of diazotetramic acids **1** of different structures was used. It can be observed that the 5-monosubstituted diazo derivatives, and especially those with no substituents in position 5, form the target products in lower, often moderate yields (see products **3i**,**j**,**n** and **3r**,**s**,**t**) compared to the 5,5-disubstituted (spirocyclic) analogues. This result may be related to the lower stability of the less-substituted β-lactam derivatives under thermolysis conditions. Additional alkyl substituents sterically shield the ring and prevent an unwanted nucleophilic attack leading to product degradation.

In reactions with alcohols and mercaptans, the corresponding esters **3k–n**,**r** and thioesters **3o–q** were successfully obtained in moderate to high yields. The synthesis of compound **3k** was additionally carried out on a scaled-up experiment (1.5 mmol vs 0.25 mmol), allowing sufficient amounts to be obtained for further modifications (vide infra). However, a marked decrease in the yield (78% vs 96%) was observed upon scaling up.

In the case of products with two stereogenic centers (**3r–t**), the formation of a single *trans*-diastereomer was observed. According to literature data, the vicinal coupling constants in the 3,4-disubsituted β-lactam cycle have characteristic values for the two diastereomers, lying in the intervals 5.5–6.0 Hz and 1.5–2.5 Hz for the *cis* and *trans* forms, respectively [33–34]. This makes it easy to assign the stereochemistry of the products obtained. Additional confirmation was gained from X-ray analysis data for structure **3t** (Scheme 2).

**Scheme 3:**
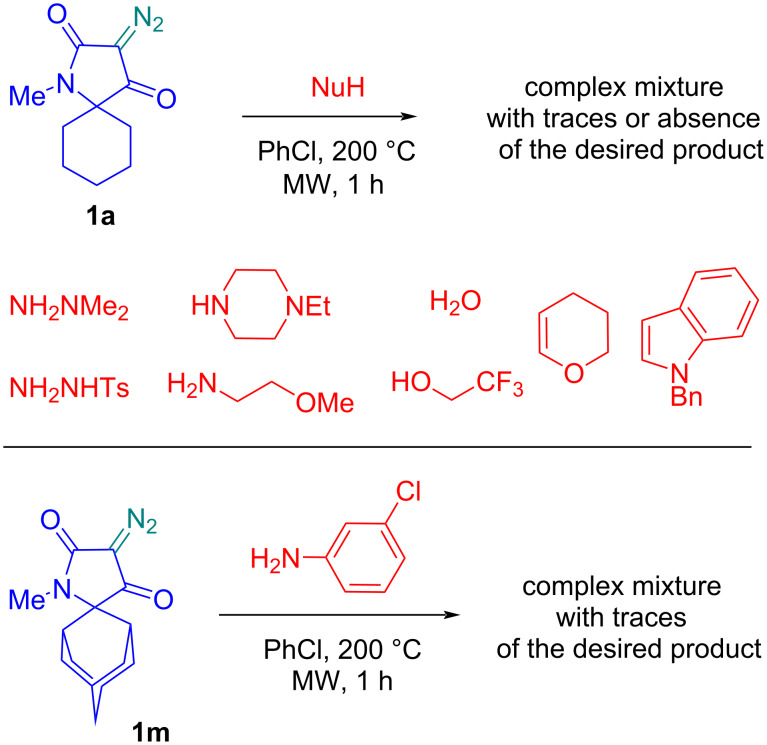
Negative results with several *N*-, *O*-, and *C*-nucleophiles and with diazo reagent **1m**.

In some cases, we were unable to isolate the target product of the reaction, which was either observed in trace amounts or was not detected in the reaction mixture at all, making it extremely difficult to interpret (Scheme 3). Negative results were observed for dimethyl and tosylhydrazine, *N*-ethylpiperazine, and β-methoxyethylamine. Attempts to obtain directly 2-oxoazetidine carboxylic acid (or its decarboxylation product) or its trifluoroethyl ester by running the synthesis with water or trifluoroethanol were also unsuccessful. Acylation of the π-excessive double bonds of *N*-alkylindole and dihydropyran by the in situ-generated ketene, previously described using carbocyclic diazodiketones [35] were also unsuccessful. Of the diazotetramic acids, only the spiro adamantane derivative **1m** was not able to form the desired β-lactam. These reactions gave complex mixtures of unidentified products.

**Scheme 4:**
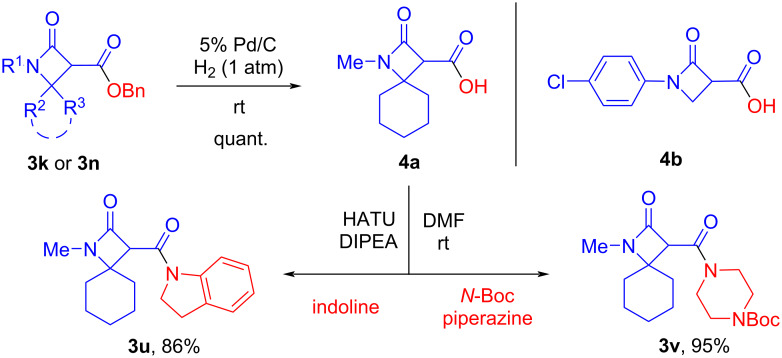
Preparation of acids **4** by hydrogenolysis of benzyl esters and examples of acid **4a** amidation.

The benzyl esters **3k** and **3n** were converted into the corresponding acids **4a**,**b** by hydrogenolysis under mild conditions, which proceeded in quantitative yields (Scheme 4). It should be noted that this method of preparing β-lactam acids compares favorably with the alkaline hydrolysis of their methyl and ethyl esters, which does not always give high yields of the target compounds. When stored individually or in solution at room temperature, the acids **4** gradually decompose and undergo decarboxylation and other accompanying processes. The example of acid **4a** demonstrates the possibility of easy amidation to form new β-lactam derivatives **3s** and **3t** (Scheme 4).

## Conclusion

We have developed a straightforward access to 2-oxoazetidine-3-carboxylic acid derivatives based on the thermally promoted Wolff rearrangement of diazotetramic acids in the presence of different nucleophiles. The proposed method allows easy variation of the substituent at the exocyclic carbonyl group by preformed ring contraction and interaction of the intermediate ketene with the selected nucleophile. Various aromatic and aliphatic amines as well as alcohols and thiols can be used as nucleophiles. 5-Monosubstituted diazotetramic acids give exclusively *trans*-diastereomeric β-lactam products. The use of variously substituted diazotetramic acids, including their spirocyclic derivatives, provides access to a new structural diversity of medically relevant β-lactam derivatives. The possibility of transforming the obtained benzyl esters into 2-oxoazetidine-3-carboxylic acids and their subsequent amidation has been demonstrated.

## Supporting Information

Deposition Number CCDC 2323689 (for **3t**) contains the supplementary crystallographic data for this paper. These data are provided free of charge by the joint Cambridge Crystallographic Data Centre and Fachinformationszentrum Karlsruhe Access Structures service http://www.ccdc.cam.ac.uk/structures.

File 1 General experimental information, X-ray crystallographic data, synthetic procedures, analytical data and NMR spectra for the reported compounds.

## Data Availability

All data that supports the findings of this study is available in the published article and/or the supporting information to this article.
