# Supplementary material for: Access to 2-oxoazetidine-3-carboxylic acid derivatives via thermal microwave-assisted Wolff rearrangement of 3-diazotetramic acids in the presence of nucleophiles
Source: Beilstein J Org Chem. 2024 Aug 5;20:1894–9. doi: 10.3762/bjoc.20.164 (PMC11318632; doi:10.3762/bjoc.20.164)
Supplement: File 1 — General experimental information, X-ray crystallographic data, synthetic procedures, analytical data and NMR spectra for the reported compounds. [file Beilstein_J_Org_Chem-20-1894-s001.pdf]

BEILSTEIN JOURNAL OF ORGANIC CHEMISTRY

# Supporting Information

for

# Access to 2-oxoazetidine-3-carboxylic acid derivatives via thermal microwave-assisted Wolff rearrangement of 3-diazotetramic acids in the presence of nucleophiles

Ivan Lyutin, Vasilisa Krivovicheva, Grigory Kantin and Dmitry Dar'in

*Beilstein J. Org. Chem.* **2024,** *20,* 1894–1899. doi:10.3762/bjoc.20.164

# General experimental information, X-ray crystallographic data, synthetic procedures, analytical data and NMR spectra for the reported compounds

License and Terms: This is a supporting information file under the terms of the Creative Commons Attribution License (https://creativecommons.org/licenses/by/4.0). Please note that the reuse, redistribution and reproduction in particular requires that the author(s) and source are credited and that individual graphics may be subject to special legal provisions.
The license is subject to the *Beilstein Journal of Organic Chemistry* terms and conditions: (https://www.beilstein-journals.org/bjoc/terms)

**Table of contents**

1. General information ........ S2
2. Experimental procedures ........ S3
   2.1. Diazotetramic acids **1a–m** ........ S3
   2.2. List of source nucleophiles. ........ S4
   2.3. Preparation of β-lactams **3a–t** ........ S5
   2.5. Preparation of β-lactams **3u,v** from acid **4a** ........ S14
3. NMR data ........ S15
   3.1. NMR spectra of β-lactams **3a**–**v**. ........ S15
   3.1. NMR spectra of β-lactamic acids **4a** and **4b** ........ S41
4. Crystal structure data for compound **3t** ........ S43
5. References ........ S45

## 1. General information

**Solvents:** Solvents were obtained from commercial suppliers. Chlorobenzene (PhCl) was dried by distillation according to the standard protocol and stored over molecular sieves (4Å).

**Reagents:** All reagents were used as purchased from commercial suppliers.

**Nuclear magnetic resonance spectroscopy:** NMR spectroscopic data were recorded with a Bruker Avance III 400 MHz spectrometer (400.13 MHz for $^{1}H$ and NOESY 100.61 MHz for $^{13}C\{^{1}H\}$ and 376.50 MHz for $^{19}F\{^{1}H\}$) in $CDCl_3$ and DMSO-$d_6$ and were referenced to residual solvent proton signals ($\delta_H$ = 7.26 and 2.50, respectively) and solvent carbon signals ($\delta_C$ = 77.16 and 39.52, respectively).

**Melting points:** Melting points were determined with a melting point apparatus REACH Devices RD-MP in the open capillary tubes.

**Mass spectrometry:** HRMS were recorded using a microOTOF-Q spectrometer (Brucker); ionization by electrospray, positive detection.

**X-ray crystallography:** Single crystal X-ray data were obtained using an Agilent Technologies SuperNova Atlas and an Agilent Technologies Xcalibur Eos diffractometers at a temperature of 100 K.

**Thin layer chromatography:** Thin layer chromatography (TLC) was performed on aluminum-backed pre-coated plates with silica gel 60 $F_{254}$ with suitable solvent system and was visualized using UV fluorescence.

**Column chromatography:** Column chromatography was carried out with silica gel grade 60 (0.040–0.063 mm) 230–400 mesh using Biotage Isolera Prime instrument.

**Microwave reactor:** Microwave-assisted reactions were performed using Microwave Synthesizer CEM Discover® 2.0 (standard mode – irradiating the sample with the highest available power for the shortest period of time).

## 2. Experimental procedures

2.1. Diazotetramic acids **1a–m**

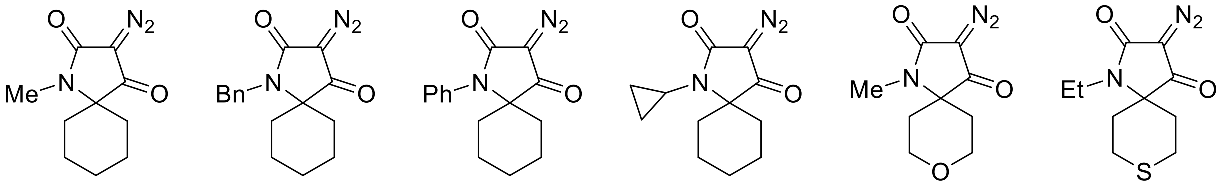

**1a** **1b** **1c** **1d** **1e** **1f**

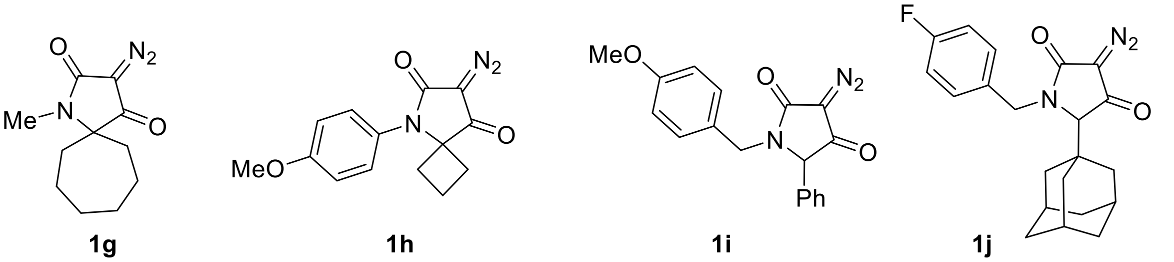

**1g** **1h** **1i** **1j**

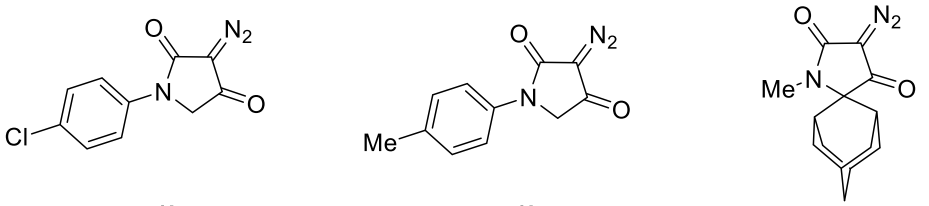

**1k** **1l** **1m**

The diazotetramic acids **1a–m** were obtained as described previously[1].

2.2. List of source nucleophiles.

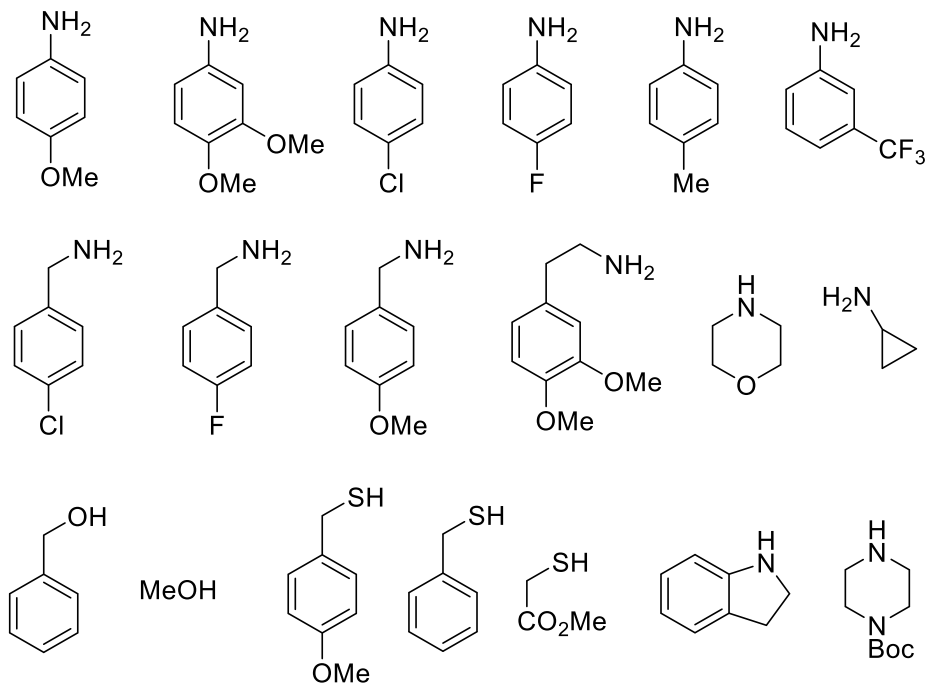

2.3. Preparation of β-lactams **3a–t**

**General procedure 1: preparation of β-lactams 3a–t**

A solution of the corresponding diazotetramic acid **1** (0.25 mmol, 1 equiv) and the corresponding nucleophile (0.275 mmol, 1.1 equiv for **3a–k,m–r** or 7.5 mmol, 30 equiv for **3l**) in dry PhCl (1 mL) was placed in a sealed vial (10 mL) equipped with a stirring bar. The sample was placed in the microwave reactor and irradiated using the "standard" mode at 200 °C for 1 hour. The solvent was evaporated under reduced pressure and the residue was purified by column chromatography (absorption at λ = 214 nm).

*N-(4-Methoxyphenyl)-1-methyl-2-oxo-1-azaspiro[3.5]nonane-3-carboxamide (**3a**).* Obtained according to GP1 from diazotetramic acid **1a** (52 mg, 0.25 mmol, 1 equiv) and *p*-anisidine (34 mg, 0.275 mmol, 1.1 equiv). Column chromatography was carried out on silica gel, eluent: *n*-hexane/acetone, from 3 to 45% of acetone. Yield: 63 mg (83%). White solid; mp 157.1–158.3 °C. $^1$H NMR (400 MHz, Chloroform-*d*) δ 8.30 (s, 1H), 7.62 – 7.36 (m, 2H), 6.97 – 6.61 (m, 2H), 3.79 (s, 3H), 3.65 (s, 1H), 2.84 (s, 3H), 2.12 – 1.97 (m, 1H), 1.98 – 1.84 (m, 1H), 1.84 – 1.61 (m, 4H), 1.37 – 1.17 (m, 2H). $^{13}$C NMR (101 MHz, Chloroform-*d*) δ 165.5, 163.4, 156.7, 130.7, 122.0, 114.2, 63.8, 63.6, 55.6, 35.9, 30.6, 24.8, 24.6, 23.9, 23.0. HRMS (ESI) *m/z*: $[M+Na]^+$ Calc. for $C_{17}H_{22}N_2NaO_3$ 325.1523; Found 325.1534.

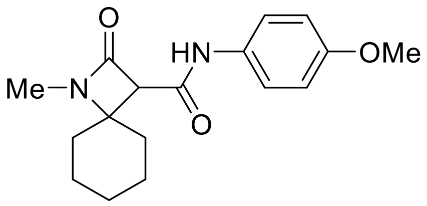


*1-Cyclopropyl-2-oxo-N-(p-tolyl)-1-azaspiro[3.5]nonane-3-carboxamide (**3b**). Obtained* according to GP1 from diazotetramic acid **1d** (58 mg, 0.25 mmol, 1 equiv) and *p*-toluidine (29 mg, 0.275 mmol, 1.1 equiv). Column chromatography was carried out on silica gel, eluent: *n*-hexane/acetone, from 5 to 25% of acetone. Yield: 58 mg (74%). Reddish amorphous solid. $^1$H NMR (400 MHz, Chloroform-*d*) δ 8.35 (s, 1H), 7.45 – 7.37 (m, 2H), 7.16 – 7.09 (m, 2H), 3.60 (s, 1H), 2.37 (tt, *J* = 7.3, 3.9 Hz, 1H), 2.31 (s, 3H), 2.09 – 1.80 (m, 5H), 1.80 – 1.60 (m, 4H), 1.36 – 1.22 (m, 1H), 1.07 – 0.97 (m, 1H), 0.97 – 0.88 (m, 1H), 0.86 – 0.70 (m, 2H). $^{13}$C NMR (126 MHz, Chloroform-*d*) δ 166.1, 163.4, 135.0, 134.4, 129.6, 120.3, 65.3, 64.0, 36.9, 31.5, 24.9, 23.9, 23.0, 21.4, 21.0, 5.7, 5.5. HRMS (ESI) *m/z*: $[M+Na]^+$ Calc. for $C_{19}H_{24}N_2NaO_2$ 335.1730; Found 335.1729.

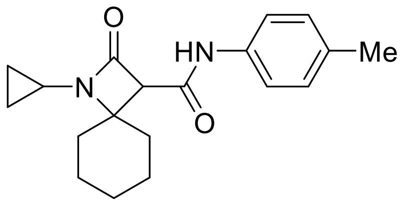

*1-Benzyl-N-cyclopropyl-2-oxo-1-azaspiro[3.5]nonane-3-carboxamide* (***3c***). Obtained according to GP1 from diazotetramic acid **1b** (71 mg, 0.25 mmol, 1 equiv) and cyclopropylamine (16 mg, 0.275 mmol, 1.1 equiv). Column chromatography was carried out on silica gel, eluent: *n*-pentane/acetone, from 5 to 40% of acetone. Yield: 62 mg (79%). Reddish oil. $^{1}$H NMR (400 MHz, Chloroform-*d*) δ 7.38 – 7.16 (m, 5H), 6.71 (s, 1H), 4.39 (d, $J$ = 15.7 Hz, 1H), 4.28 (d, $J$ = 15.7 Hz, 1H), 3.52 (s, 1H), 2.71 (tq, $J$ = 6.9, 3.5 Hz, 1H), 1.86 – 1.70 (m, 2H), 1.67 – 1.35 (m, 7H), 1.16 – 0.99 (m, 1H), 0.81 – 0.71 (m, 2H), 0.57 – 0.47 (m, 2H). $^{13}$C NMR (101 MHz, Chloroform-*d*) δ 166.9, 165.5, 136.9, 128.8, 127.9, 127.7, 64.7, 63.7, 43.0, 37.1, 31.2, 24.7, 23.8, 22.9, 22.6, 6.7, 6.5. HRMS (ESI) *m/z*: [M+H]$^+$ Calc. for $C_{19}H_{25}N_2O_2$ 313.1911; Found 313.1912.

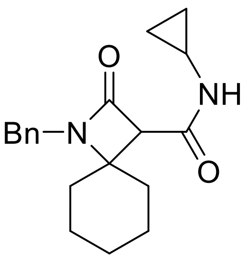


*N-(3,4-Dimethoxyphenyl)-1-methyl-2-oxo-7-oxa-1-azaspiro[3.5]nonane-3-carboxamide* (***3d***). Obtained according to GP1 from diazotetramic acid **1e** (52 mg, 0.25 mmol, 1 equiv) and 3,4-dimethoxyaniline (42 mg, 0.275 mmol, 1.1 equiv). Column chromatography was carried out on silica gel, eluent: *n*-hexane/acetone, from 3 to 25% of acetone. Yield: 81 mg (97%). Light brown solid; mp 217.5–219.3 °C. $^{1}$H NMR (400 MHz, Chloroform-*d*) δ 8.48 (s, 1H), 7.21 – 7.16 (m, 1H), 7.00 – 6.93 (m, 1H), 6.80 – 6.71 (m, 1H), 4.12 – 4.04 (m, 1H), 4.03 – 3.96 (m, 1H), 3.92 – 3.85 (m, 1H), 3.83 (s, 3H), 3.82 (s, 3H), 3.77 (s, 1H), 3.72 – 3.64 (m, 1H), 2.79 (s, 3H), 2.15 – 2.02 (m, 1H), 2.00 – 1.90 (m, 1H), 1.90 – 1.82 (m, 1H), 1.66 – 1.60 (m, 1H). $^{13}$C NMR (101 MHz, Chloroform-*d*) δ 165.0, 162.9, 149.1, 146.2, 130.9, 112.3, 111.3, 104.9, 65.3, 65.0, 63.7, 60.9, 56.1, 56.0, 35.3, 30.4, 24.1. HRMS (ESI) *m/z*: [M+Na]$^+$ Calc. for $C_{18}H_{24}N_2NaO_4$ 357.1421; Found 357.1418.

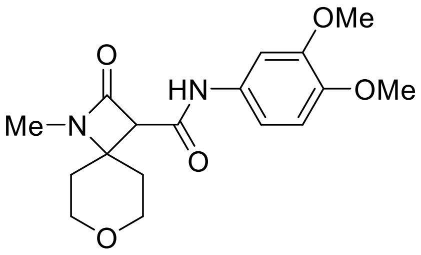


*1-Ethyl-N-(4-methoxybenzyl)-2-oxo-7-thia-1-azaspiro[3.5]nonane-3-carboxamide* (***3e***). Obtained according to GP1 from diazotetramic acid **1f** (60 mg, 0.25 mmol, 1 equiv) and *p*-methoxybenzylamine (38 mg, 0.275 mmol, 1.1 equiv). Column chromatography was carried out on silica gel, eluent: *n*-pentane/acetone, from 5 to 50% of acetone. Yield: 63 mg (68%). Orange solid; mp 153.0–154.0 °C. $^{1}$H NMR (400 MHz, Chloroform-*d*) δ 7.22 – 7.17 (m, 2H), 6.91 (s, 1H), 6.87 – 6.82 (m, 2H), 4.42 – 4.33 (m, 2H), 3.79 (s, 3H), 3.54 (s, 1H), 3.51 – 3.40 (m, 1H), 3.30 – 3.09 (m, 2H), 2.96 – 2.84 (m, 1H), 2.72 – 2.63 (m, 1H), 2.54

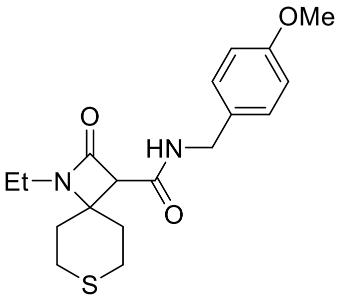

– 2.44 (m, 1H), 2.19 – 2.06 (m, 2H), 2.06 – 1.93 (m, 2H), 1.23 (t, *J* = 7.3 Hz, 3H). $^{13}C$ NMR (101 MHz, Chloroform-*d*) δ 167.0, 161.4, 135.1, 128.9, 128.8, 128.8, 67.5, 62.4, 62.0, 38.7, 34.2, 32.4, 27.0, 26.0, 15.0. HRMS (ESI) *m/z*: $[M+Na]^+$ Calc. for $C_{18}H_{24}N_2NaO_3S$ 371.1400; Found 371.1397.

*N-(3,4-Dimethoxyphenethyl)-2-oxo-1-phenyl-1-azaspiro[3.5]nonane-3-carboxamide* ***(3f)***.

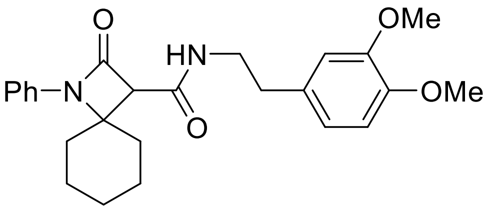


Obtained according to GP1 from diazotetramic acid **1c** (67 mg, 0.25 mmol, 1 equiv) and 3,4-dimethoxyphenethylamine (50 mg, 0.275 mmol, 1.1 equiv.). Column chromatography was carried out on silica gel, eluent: *n*-hexane/acetone, from 5 to 35% of acetone. Yield: 67 mg (54%). Yellowish oil. $^1H$ NMR (400 MHz, Chloroform-*d*) δ 7.52 – 7.47 (m, 2H), 7.37 – 7.29 (m, 2H), 7.18 – 7.10 (m, 1H), 6.79 – 6.72 (m, 3H), 6.65 – 6.57 (br.m, 1H), 3.84 (s, 3H), 3.82 (s, 3H), 3.64 (s, 1H), 3.61 – 3.45 (m, 2H), 2.88 – 2.72 (m, 2H), 2.22 – 2.15 (m, 1H), 2.07 – 2.01 (m, 1H), 1.96 – 1.50 (m, 7H), 1.23 – 1.13 (m, 1H). $^{13}C$ NMR (101 MHz, Chloroform-*d*) δ 165.0, 163.8, 149.2, 147.9, 136.5, 131.3, 129.3, 125.2, 120.8, 119.9, 112.0, 111.5, 67.0, 64.6, 56.0, 56.0, 41.0, 36.9, 35.4, 30.7, 24.9, 23.9, 23.0. HRMS (ESI) *m/z*: $[M+Na]^+$ Calc. for $C_{25}H_{30}N_2NaO_4$ 445.2100; Found 445.2104.

*N-(4-Chlorophenyl)-1-(4-methoxyphenyl)-2-oxo-1-azaspiro[3.3]heptane-3-carboxamide* ***(3g)***.

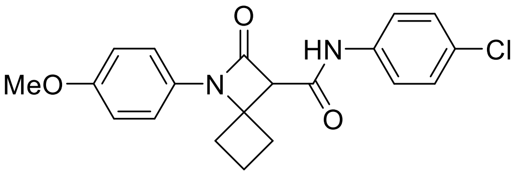


Obtained according to GP1 from diazotetramic acid **1h** (68 mg, 0.25 mmol, 1 equiv) and *p*-chloroaniline (35 mg, 0.275 mmol, 1.1 equiv). Column chromatography was carried out on silica gel, eluent: *n*-hexane/acetone, from 3 to 35% of acetone. Yield: 63 mg (68%). Brown solid; mp 167.0–167.9 °C. $^1H$ NMR (400 MHz, Chloroform-*d*) δ 8.49 (s, 1H), 7.60 – 7.46 (m, 4H), 7.37 – 7.21 (m, 2H), 6.98 – 6.87 (m, 2H), 4.09 (s, 1H), 3.82 (s, 3H), 2.98 – 2.88 (m, 1H), 2.78 – 2.59 (m, 2H), 2.51 – 2.40 (m, 1H), 2.24 – 2.10 (m, 1H), 2.02 – 1.87 (m, 1H). $^{13}C$ NMR (101 MHz, Chloroform-*d*) δ 163.4, 162.8, 157.4, 135.9, 129.9, 129.4, 129.2, 121.5, 120.8, 114.8, 65.8, 63.3, 55.6, 32.2, 28.1, 13.5. HRMS (ESI) *m/z*: $[M+Na]^+$ Calc. for $C_{20}H_{19}ClN_2NaO_3$ 393.0976; Found 393.0981.

*N-(4-Fluorobenzyl)-1-methyl-2-oxo-1-azaspiro[3.6]decane-3-carboxamide (**3h**).*

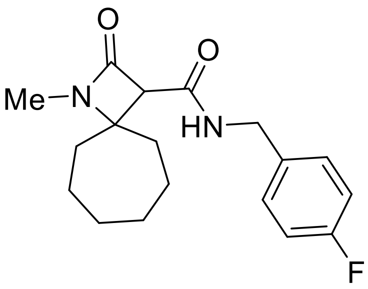

Obtained according to GP1 from diazotetramic acid **1g** (55 mg, 0.25 mmol, 1 equiv) and *p*-fluorobenzylamine (34 mg, 0.275 mmol, 1.1 equiv). Column chromatography was carried out on silica gel, eluent: *n*-hexane/acetone, from 5 to 30% of acetone. Yield: 68 mg (85%). Yellow oil. $^{1}$H NMR (400 MHz, Chloroform-*d*) δ 7.24 – 7.18 (m, 2H), 7.00 (s, 1H), 6.98 – 6.93 (m, 2H), 4.42 – 4.32 (m, 2H), 3.57 (s, 1H), 2.73 (s, 3H), 2.05 – 1.92 (m, 2H), 1.92 – 1.82 (m, 1H), 1.79 – 1.44 (m, 9H). $^{13}$C NMR (101 MHz, Chloroform-*d*) δ 165.2, 164.9, 161.9 (d, $^{1}J_{C\text{–}F}$ = 245.4 Hz), 133.7 (d, $^{4}J_{C\text{–}F}$ = 2.9 Hz), 129.2 (d, $^{3}J_{C\text{–}F}$ = 8.1 Hz), 115.2 (d, $^{2}J_{C\text{–}F}$ = 21.4 Hz), 66.0, 64.0, 42.2, 37.4, 33.6, 33.6, 29.7, 24.0, 22.8, 22.8. $^{19}$F NMR (376 MHz, Chloroform-*d*) δ -115.11. HRMS (ESI) *m/z*: [M+Na]$^{+}$ Calc. for $C_{18}H_{23}FN_2NaO_2$ 341.1636; Found 341.1632.

*1-(4-Chlorophenyl)-N-(4-fluorophenyl)-2-oxoazetidine-3-carboxamide (**3i**).* Obtained

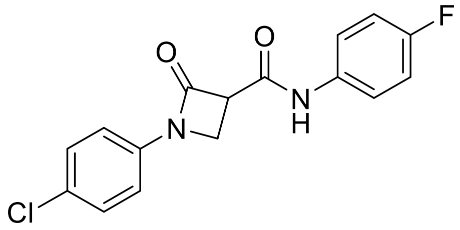

according to GP1 from diazotetramic acid **1k** (59 mg, 0.25 mmol, 1 equiv) and *p*-fluoroaniline (38 mg, 0.275 mmol, 1.1 equiv). Column chromatography was carried out on silica gel, eluent: *n*-pentane/ethyl acetate, from 5 to 35% of ethyl acetate. Yield: 32 mg (40%). Beige solid; mp 223.0–224.7 °C. $^{1}$H NMR (400 MHz, DMSO-$d_6$) δ 10.34 (s, 1H), 7.66 – 7.58 (m, 2H), 7.37 – 7.31 (m, 4H), 7.07 – 7.00 (m, 2H), 4.37 (dd, *J* = 5.4, 2.6 Hz, 1H), 3.91 (dd, *J* = 5.6, 2.6 Hz, 1H), 3.81 (t, *J* = 5.6 Hz, 1H). $^{13}$C NMR (101 MHz, DMSO-$d_6$) δ 163.8, 160.8, 158.2 (d, $^{1}J_{C\text{–}F}$ = 241.3 Hz), 136.4, 134.7 (d, $^{4}J_{C\text{–}F}$ = 2.5 Hz), 128.8, 127.7, 120.9 (d, $^{3}J_{C\text{–}F}$ = 7.7 Hz), 117.4, 115.0 (d, $^{2}J_{C\text{–}F}$ = 22.2 Hz), 54.6, 41.2. $^{19}$F NMR (376 MHz, DMSO-$d_6$) δ -118.40. HRMS (ESI) *m/z*: [M+Na]$^{+}$ Calc. for $C_{16}H_{12}ClFN_2NaO_2$ 341.0464; Found 341.0471.

*2-Oxo-1-(p-tolyl)-N-(3-(trifluoromethyl)phenyl)azetidine-3-carboxamide (**3j**). Obtained*

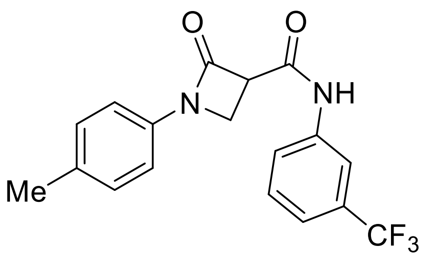

according to GP1 from diazotetramic acid **1l** (54 mg, 0.25 mmol, 1 equiv) and nucleophile **2h** (44 mg, 0.275 mmol, 1.1 equiv). The sample obtained after chromatographic isolation was triturated with *n*-hexane/diethyl ether (1:1, 2 × 2 mL). Yield: 23 mg (26%). White solid; mp 217.1–218.0 °C. $^{1}$H NMR (400 MHz, DMSO-$d_6$) δ 10.45 (s, 1H), 8.05 – 8.00 (m, 1H), 7.80 – 7.74 (m, 1H), 7.45 – 7.37 (m, 1H), 7.32 – 7.24 (m, 1H), 7.24

– 7.16 (m, 2H), 7.13 – 7.04 (m, 2H), 4.34 (dd, $J$ = 5.4, 2.6 Hz, 2H), 3.93 (dd, $J$ = 5.5, 2.6 Hz, 2H), 3.73 (t, $J$ = 5.6 Hz, 1H), 2.25 (s, 3H). $^{13}$C NMR (101 MHz, DMSO-$d_6$) δ 164.6, 160.0, 139.1, 134.1 (q, $^1J_{C–F}$ = 233.0 Hz), 129.9 (q, $^2J_{C–F}$ = 31.0 Hz), 129.2, 122.4, 119.64 (q, $^3J_{C–F}$ = 2.9 Hz), 115.8, 115.6 (q, $^3J_{C–F}$ = 3.4 Hz), 54.4, 40.7, 20.4. $^{19}$F NMR (376 MHz, DMSO-$d_6$) δ -62.06. HRMS (ESI) *m/z*: [M+Na]$^+$ Calc. for $C_{18}H_{15}F_3N_2NaO_2$ 371.0978; Found 371.0982.

*Benzyl 1-methyl-2-oxo-1-azaspiro[3.5]nonane-3-carboxylate (**3k**).* Obtained according to GP1 from diazotetramic acid **1a** (52 mg, 0.25 mmol, 1 equiv) and benzyl alcohol (30 mg, 0.275 mmol, 1.1 equiv). Column chromatography was carried out on silica gel, eluent: *n*-hexane/acetone, from 3 to 25% of acetone. Yield: 70 mg (96%). Yellow oil. $^1$H NMR (400 MHz, Chloroform-*d*) δ 7.38 – 7.29 (m, 5H), 5.22 (d, $J$ = 12.2 Hz, 1H), 5.11 (d, $J$ = 12.2 Hz, 1H), 3.64 (s, 1H), 2.71 (s, 3H), 1.99 – 1.91 (m, 1H), 1.84 – 1.76 (m, 1H), 1.73 – 1.67 (m, 1H), 1.63 – 1.47 (m, 4H), 1.45 – 1.34 (m, 1H), 1.17 – 0.99 (m, 2H). $^{13}$C NMR (101 MHz, Chloroform-*d*) δ 167.4, 162.2, 135.3, 128.7, 128.64, 128.5, 67.1, 62.8, 62.3, 35.7, 29.4, 24.8, 24.0, 23.9, 23.2. HRMS (ESI) *m/z*: [M+Na]$^+$ Calc. for $C_{17}H_{21}NNaO_3$ 310.1414; Found 310.1426.

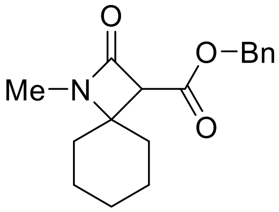


The synthesis of **3k** was additionally performed on a 6-fold scale (1.5 mmol) and yielded 336 mg (78%).

*Methyl 1-methyl-2-oxo-1-azaspiro[3.5]nonane-3-carboxylate (**3l**).* Obtained according to GP1 from diazotetramic acid **1a** (52 mg, 0.25 mmol, 1 equiv) and methanol (240 mg, 7.5 mmol, 30 equiv). Column chromatography was carried out on silica gel, eluent: *n*-pentane/acetone, from 5 to 40% of acetone. Yield: 69 mg (64%). Light brown oil. $^1$H NMR (400 MHz, Chloroform-*d*) δ 3.71 (s, 3H), 3.59 (s, 1H), 2.71 (s, 3H), 2.04 – 1.91 (m, 1H), 1.87 – 1.51 (m, 6H), 1.48 – 1.34 (m, 1H), 1.27 – 1.08 (m, 2H). $^{13}$C NMR (101 MHz, Chloroform-*d*) δ 167.9, 162.3, 62.7, 62.1, 52.2, 35.7, 29.5, 24.8, 23.9, 23.3. HRMS (ESI) *m/z*: [M+Na]$^+$ Calc. for $C_{11}H_{17}NNaO_3$ 234.1101; Found 234.1109.

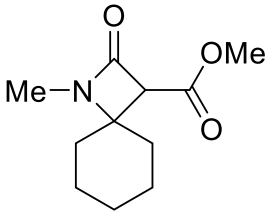

*Benzyl 1-ethyl-2-oxo-7-thia-1-azaspiro[3.5]nonane-3-carboxylate (**3m**).* Obtained according to GP1 from diazotetramic acid **1f** (60 mg, 0.25 mmol, 1 equiv) and benzyl alcohol (30 mg, 0.275 mmol, 1.1 equiv). Column chromatography was carried out on silica gel, eluent: *n*-hexane/acetone, from 3 to 25% of acetone. Yield: 65 mg (81%). Red oil. $^{1}$H NMR (400 MHz, Chloroform-*d*) δ 7.48 – 7.32 (m, 5H), 5.24 (d, *J* = 12.1 Hz, 1H), 5.12 (d, *J* = 12.1 Hz, 1H), 3.65 (s, 1H), 3.21 (q, *J* = 7.3 Hz, 2H), 2.82 – 2.63 (m, 2H), 2.42 – 2.32 (m, 3H), 2.19 – 2.08 (m, 1H), 2.02 – 1.87 (m, 2H), 1.23 (t, *J* = 7.3 Hz, 3H). $^{13}$C NMR (101 MHz, Chloroform-*d*) δ 167.0, 161.4, 135.1, 128.9, 128.8, 128.8, 67.5, 62.4, 62.0, 38.7, 34.2, 32.4, 27.0, 26.0, 15.0. HRMS (ESI) *m/z*: [M+Na]$^+$ Calc. for $C_{17}H_{21}NNaO_3S$ 342.1134; Found 342.1148.

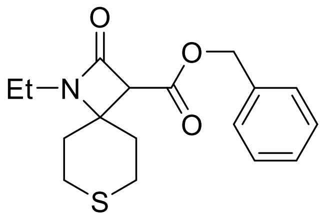


*Benzyl 1-(4-chlorophenyl)-2-oxoazetidine-3-carboxylate (**3n**).* Obtained according to GP1 from diazotetramic acid **1k** (59 mg, 0.25 mmol, 1 equiv) and benzyl alcohol (30 mg, 0.275 mmol, 1.1 equiv). Column chromatography was carried out on silica gel, eluent: *n*-pentane/acetone, from 3 to 20% of acetone. Yield: 42 mg (41%). Yellowish solid; mp 122.1–123.8 °C. $^{1}$H NMR (400 MHz, Chloroform-*d*) δ 7.45 – 7.22 (m, 9H), 5.27 (d, *J* = 12.3 Hz, 1H), 5.23 (d, *J* = 12.3 Hz, 1H), 4.25 (dd, *J* = 5.7, 2.9 Hz, 1H), 3.95 (dd, *J* = 5.9, 2.9 Hz, 1H), 3.75 (t, *J* = 5.8 Hz, 1H). $^{13}$C NMR (101 MHz, Chloroform-*d*) δ 166.6, 158.7, 136.4, 135.2, 129.6, 129.4, 128.8, 128.6, 128.4, 117.8, 67.7, 53.5, 41.6. HRMS (ESI) *m/z*: [M+Na]$^+$ Calc. for $C_{17}H_{14}ClNNaO_3$ 338.0554; Found 338.0561.

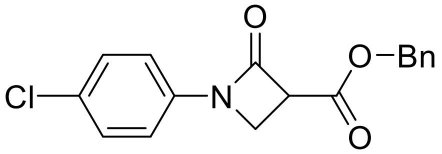


*S-(4-Methoxybenzyl) 1-methyl-2-oxo-1-azaspiro[3.5]nonane-3-carbothioate (**3o**).* Obtained according to GP1 from diazo tetramicacid **1a** (52 mg, 0.25 mmol, 1 equiv) and (*p*-methoxybenzyl)mercaptan (42 mg, 0.275 mmol, 1.1 equiv). Column chromatography was carried out on silica gel, eluent: *n*-hexane/acetone, from 3 to 20% of acetone. Yield: 40 mg (48%). Yellow oil. $^{1}$H NMR (400 MHz, Chloroform-*d*) δ 7.23 – 7.12 (m, 2H), 6.88 – 6.70 (m, 2H), 4.15 (d, *J* = 13.7 Hz, 1H), 4.09 (d, *J* = 13.7 Hz, 1H), 3.81 (s, 1H), 3.76 (s, 3H), 2.71 (s, 3H), 2.11 – 1.97 (m, 1H), 1.88 – 1.74 (m, 1H), 1.76 – 1.67 (m, 1H), 1.68 – 1.36 (m, 5H), 1.28 – 1.05 (m, 2H). $^{13}$C NMR (101 MHz, Chloroform-*d*) δ 192.1, 162.2, 159.0, 130.2, 128.8, 114.1 69.8, 63.3, 55.3, 35.9, 33.4, 29.2, 24.8, 24.0, 23.9, 23.3. HRMS (ESI) *m/z*: [M+Na]$^+$ Calc. for $C_{18}H_{23}NNaO_3S$ 356.1291; Found 356.1294.

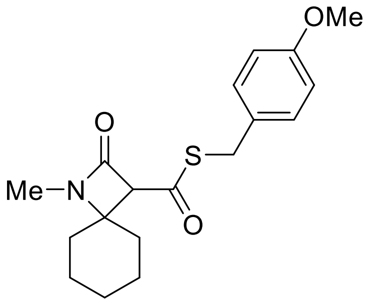

*S-Benzyl 1-ethyl-2-oxo-7-thia-1-azaspiro[3.5]nonane-3-carbothioate (**3p**).* Obtained

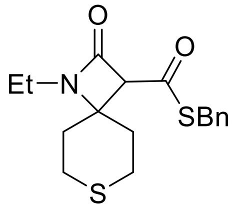


according to GP1 from diazotetramic acid **1f** (60 mg, 0.25 mmol, 1 equiv) and benzyl mercaptan (34 mg, 0.275 mmol, 1.1 equiv). Column chromatography was carried out on silica gel, eluent: *n*-hexane/acetone, from 5 to 25% of acetone. Yield: 56 mg (67%). Yellow oil. $^1$H NMR (400 MHz, Chloroform-*d*) δ 7.36 – 7.21 (m, 5H), 4.23 (d, *J* = 13.8 Hz, 1H), 4.16 (d, *J* = 13.7 Hz, 1H), 3.85 (s, 1H), 3.21 (q, *J* = 7.3 Hz, 2H), 2.83 (ddd, *J* = 14.5, 12.4, 2.5 Hz, 1H), 2.70 (dtd, *J* = 14.0, 3.8, 2.4 Hz, 1H), 2.58 – 2.43 (m, 2H), 2.41 – 2.32 (m, 1H), 2.25 – 2.09 (m, 1H), 2.00 (ddd, *J* = 13.2, 4.2, 2.1 Hz, 1H), 1.97 – 1.88 (m, 1H), 1.24 (t, *J* = 7.3 Hz, 3H). $^{13}$C NMR (101 MHz, Chloroform-*d*) δ 191.6, 161.3, 136.5, 129.0, 128.7, 127.5, 69.3, 62.9, 38.6, 34.1, 34.1, 32.1, 26.8, 26.0, 14.9. HRMS (ESI) *m/z*: $[M+H]^+$ Calc. for $C_{17}H_{22}NO_2S_2$ 336.1086; Found 336.1085.

*Methyl 2-((1-benzyl-2-oxo-1-azaspiro[3.5]nonane-3-carbonyl)thio)acetate (**3q**).* Obtained

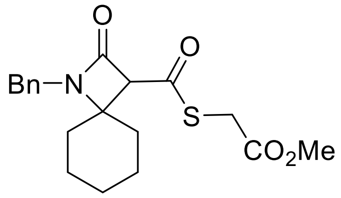


according to GP1 from diazotetramic acid **1b** (71 mg, 0.25 mmol, 1 equiv) and methyl thioglycolate (29 mg, 0.275 mmol, 1.1 equiv). Column chromatography was carried out on silica gel, eluent: *n*-hexane/acetone, from 5 to 25% of acetone. Yield: 50 mg (55%). Yellow oil. $^1$H NMR (400 MHz, Chloroform-*d*) δ $^1$H NMR (400 MHz, Chloroform-*d*) δ 7.40 – 7.25 (m, 5H), 4.42 (d, *J* = 15.7 Hz, 1H), 4.33 (d, *J* = 15.7 Hz, 1H), 3.97 (s, 1H), 3.88 (d, *J* = 16.1 Hz, 1H), 3.75 (s, 3H), 3.70 (d, *J* = 16.1 Hz, 1H), 2.10 – 2.01 (m, 1H), 1.84 – 1.72 (m, 1H), 1.68 – 1.55 (m, 4H), 1.49 – 1.34 (m, 2H), 1.28 (tdd, *J* = 13.0, 7.5, 3.1 Hz, 1H), 1.07 (qt, *J* = 12.2, 3.6 Hz, 1H). $^{13}$C NMR (101 MHz, Chloroform-*d*) δ 190.8, 168.5, 161.9, 136.7, 128.7, 127.9, 127.7, 69.7, 65.2, 52.8, 42.9, 37.1, 31.6, 30.2, 24.6, 24.1, 23.2. HRMS (ESI) *m/z*: $[M+H]^+$ Calc. for $C_{19}H_{24}NO_4S$ 362.1421; Found 362.1415.

*Benzyl (±)-trans-1-(4-methoxybenzyl)-2-oxo-4-phenylazetidine-3-carboxylate (**3r**).*

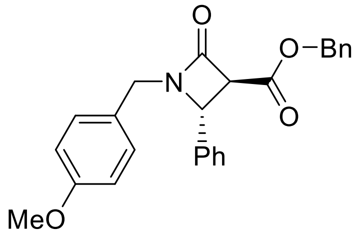


Obtained according to GP1 from diazotetramic acid **1i** (80 mg, 0.25 mmol, 1 equiv) and benzyl alcohol (30 mg, 0.275 mmol, 1.1 equiv). Column chromatography was carried out on silica gel, eluent: *n*-pentane/acetone, from 3 to 20% of acetone. Yield: 46 mg (46%). Yellowish oil. $^1$H NMR (400 MHz, Chloroform-*d*) δ 7.41 – 7.32 (m, 8H), 7.26 – 7.21 (m, 2H), 7.07 – 7.02 (m, 2H), 6.79 – 6.74 (m, 2H), 5.26 (d, *J* = 12.4 Hz, 1H), 5.18 (d, *J* = 12.4 Hz, 1H), 4.81 (d, *J* = 15.1 Hz, 1H), 4.68 (d, *J* = 2.2 Hz, 1H), 3.95 (d, *J* = 1.7 Hz, 1H), 3.78 (s, 3H), 3.76 (d, *J* = 15.0 Hz,

1H),. $^{13}$C NMR (101 MHz, Chloroform-*d*) δ 166.7, 162.1, 159.3, 136.1, 135.3, 129.7, 129.2, 129.1, 128.7, 128.5, 128.3, 126.9, 126.7, 114.2, 67.5, 63.4, 56.9, 55.3, 44.4. HRMS (ESI) *m/z*: $[M+H]^+$ Calc. for $C_{25}H_{24}NO_4$ 402.1699; Found 402.1687.

*(±)-trans-1-(4-Methoxybenzyl)-3-(morpholine-4-carbonyl)-4-phenylazetidin-2-one* ***(3s)****.*

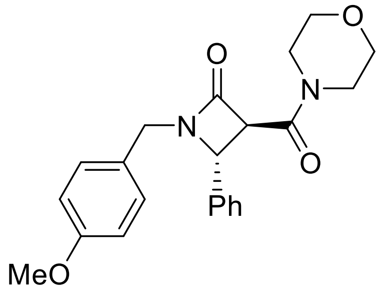


Obtained according to GP1 from diazotetramic acid **1i** (80 mg, 0.25 mmol, 1 equiv) and morpholine (22 mg, 0.275 mmol, 1.1 equiv). Column chromatography was carried out on silica gel, eluent: *n*-hexane/acetone, from 5 to 20% of acetone. Yield: 42 mg (44%). Red oil. $^{1}$H NMR (400 MHz, Chloroform-*d*) δ 7.40 – 7.31 (m, 4H), 7.30 – 7.25 (m, 1H), 7.08 – 6.99 (m, 2H), 6.83 – 6.73 (m, 2H), 5.11 (d, *J* = 1.9 Hz, 1H), 4.68 (d, *J* = 15.0 Hz, 1H), 4.05 (d, *J* = 1.8 Hz, 1H), 3.94 – 3.81 (m, 3H), 3.77 (s, 3H), 3.77 – 3.68 (m, 4H), 3.66 – 3.58 (m, 1H), 3.46 – 3.36 (m, 1H), 3.35 – 3.27 (m, 1H). $^{13}$C NMR (101 MHz, Chloroform-*d*) δ 163.7, 163.6, 136.9, 129.8, 129.1, 128.8, 126.8, 126.8, 114.3, 67.1, 66.7, 63.2, 56.8, 55.3, 46.1, 44.6, 42.5. HRMS (ESI) *m/z*: $[M+H]^+$ Calc. for $C_{22}H_{25}N_2O_4$ 381.1809; Found 381.1818.

*(±)-trans-2-(Adamantan-1-yl)-N-(4-chlorobenzyl)-1-(4-fluorobenzyl)-4-oxoazetidine-3-carboxamide* ***(3t)****.* Obtained according to GP1 from diazotetramic acid **1j** (92 mg,

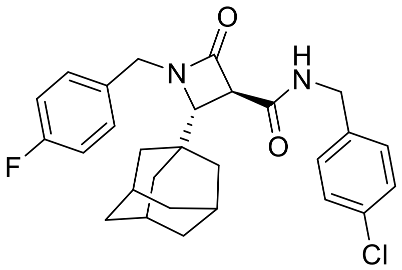


0.25 mmol, 1 equiv) and (*p*-chlorobenzyl)amine (39 mg, 0.275 mmol, 1.1 equiv). Column chromatography was carried out on silica gel, eluent: *n*-pentane/acetone, from 5 to 30% of acetone. Yield: 60 mg (50%). Reddish solid; mp 124.8–126.0 °C. $^{1}$H NMR (400 MHz, Chloroform-*d*) δ 7.32 – 7.23 (m, 3H), 7.22 – 7.14 (m, 3H), 7.05 – 6.95 (m, 2H), 6.88 (s, 1H), 4.73 (d, *J* = 15.3 Hz, 1H), 4.44 – 4.36 (m, 2H), 4.12 (d, *J* = 15.3 Hz, 1H), 3.76 (d, *J* = 1.7, 0.2 Hz, 1H), 3.48 (d, *J* = 1.6 Hz, 1H), 1.97 (s, 3H), 1.71 – 1.66 (m, 3H), 1.58 – 1.52 (m, 6H), 1.46 – 1.40 (m, 3H). $^{13}$C NMR (101 MHz, Chloroform-*d*) δ 166.5, 166.1, 162.4 (d, $^{1}J_{C-F}$ = 246.6 Hz), 136.6, 133.4, 131.3 (d, $^{4}J_{C-F}$ = 3.2 Hz), 129.9 (d, $^{3}J_{C-F}$ = 8.2 Hz), 129.1, 128.9, 115.9 (d, $^{2}J_{C-F}$ = 21.6 Hz), 65.0, 53.4, 46.6, 42.9, 38.7, 36.8, 34.3, 27.9. $^{19}$F NMR (376 MHz, Chloroform-*d*) δ -114.09. HRMS (ESI) *m/z*: $[M+Na]^+$ Calc. for $C_{28}H_{30}ClFN_2NaO_2$ 503.1872; Found 503.1871.

2.4. Preparation of β-lactamic acids **4a** and **4b**

**General procedure 2: preparation of β-lactamic acids 4a and 4b**

5% Pd/C (10 mol %) was added to the solution of corresponding benzyl ester in THF (for **3k**) or ethyl acetate (for **3o**). The inside air was replaced with $H_2$ (balloon) by three vacuum/$H_2$ cycles. The reaction mixture was stirred overnight at room temperature in an atmosphere of hydrogen. The reaction mixture was filtered through a syringe filter and the solvent was evaporated under reduced pressure.

*1-Methyl-2-oxo-1-azaspiro[3.5]nonane-3-carboxylic acid (**4a**).* Obtained according to GP2 from β-lactam **3k** (0.91 mmol, 261 mg). Yield: 179 mg (100%). White solid. $^1H$ NMR (400 MHz, Chloroform-*d*) δ 3.68 (s, 1H), 2.76 (s, 3H), 2.14 – 2.05 (m, 1H), 1.89 – 1.58 (m, 6H), 1.54 – 1.34 (m, 2H), 1.27 – 1.12 (m, 1H). $^{13}C$ NMR (101 MHz, Chloroform-*d*) δ 170.9, 163.7, 62.9, 62.6, 35.7, 29.5, 24.9, 24.2, 23.9, 23.3. HRMS (ESI) *m/z*: $[M+Na]^+$ Calc. for $C_{10}H_{15}NNaO_3$ 220.0944; Found 220.0946. The substance decomposes slowly at room temperature and should be stored at reduced temperatures.

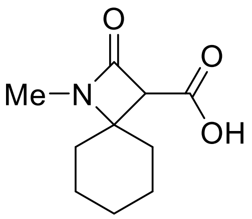


*1-(4-Chlorophenyl)-2-oxoazetidine-3-carboxylic acid (**4b**).* Obtained according to GP2 from β-lactam **3o** (0.3 mmol, 100 mg). Yield: 68 mg (100%). $^1H$ NMR (400 MHz, Chloroform-*d*) δ 10.53 (br.s, 1H), 7.36 – 7.27 (m, 4H), 4.27 (dd, *J* = 5.3, 2.7 Hz, 1H), 3.97 (dd, *J* = 5.5, 2.3 Hz, 1H), 3.81 (t, *J* = 5.6 Hz, 1H). $^{13}C$ NMR (101 MHz, Chloroform-*d*) δ 171.2, 158.6, 136.2, 130.01, 129.5, 117.9, 53.3, 41.7. HRMS (ESI) *m/z*: $[M+H]^+$ Calc. for $C_{10}H_9ClNO_3$ 226.0265; Found 226.0263. The substance decomposes slowly at room temperature and should be stored at reduced temperatures.

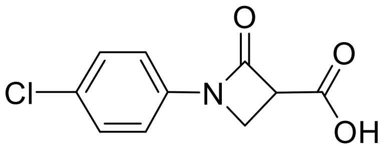

2.5. Preparation of β-lactams **3u,v** from acid **4a**

**General procedure 3: preparation of β-lactams 3u and 3v from 4a**

A solution of the β-lactamic acid **4a** (0.3 mmol, 30 mg, 1 equiv) and the corresponding nucleophile (0.32 mmol, 1.05 equiv) in dry DMF (5 mL) was added to the solution of HATU (0.33, 1.1 equiv, 127 mg) and DIPEA (0.33 mmol, 1.1 equiv, 43 mg) in dry DMF (5 mL). The reaction mixture was stirred overnight at room temperature. The solution was diluted with sat. aq. $NaHCO_3$ (15 mL), extracted with ethyl acetate, and dried over $Na_2SO_4$. The solvent was evaporated under reduced pressure and the residue was purified by column chromatography (absorption at λ = 214 nm).

*3-(Indoline-1-carbonyl)-1-methyl-1-azaspiro[3.5]nonan-2-one (**3u**).* Obtained according to GP3 from acid **4a** and indoline (38 mg, 0.32 mmol, 1.05 equiv). Column

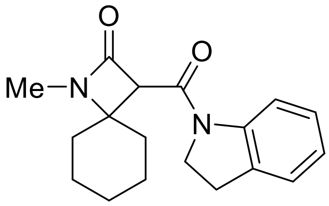


chromatography was carried out on silica gel, eluent: *n*-hexane/acetone, from 3 to 50% of acetone. Yield: 78 mg (86%). Beige solid, mp 156.3–157.8 °C. $^{1}$H NMR (400 MHz, Chloroform-*d*) δ 8.36 – 8.20 (m, 1H), 7.22 – 7.13 (m, 2H), 7.09 – 6.94 (m, 1H), 4.35 – 4.22 (m, 1H), 4.18 – 4.02 (m, 1H), 3.85 (s, 1H), 3.33 – 3.12 (m, 2H), 2.80 (s, 3H), 2.49 – 2.33 (m, 1H), 2.03 – 1.77 (m, 2H), 1.80 – 1.55 (m, 4H), 1.49 – 1.36 (m, 1H), 1.36 – 1.09 (m, 2H). $^{13}$C NMR (101 MHz, Chloroform-*d*) δ 164.6, 163.4, 142.8, 131.4, 127.8, 124.6, 124.3, 117.9, 62.9, 62.76, 49.0, 36.0, 29.1, 28.2, 24.9, 24.2, 24.1. HRMS (ESI) *m/z*: [M+Na]$^+$ Calc. for $C_{18}H_{22}N_2NaO_2$ 321.1573; Found 321.1574.

*tert-Butyl 4-(1-methyl-2-oxo-1-azaspiro[3.5]nonane-3-carbonyl)piperazine-1-carboxylate*

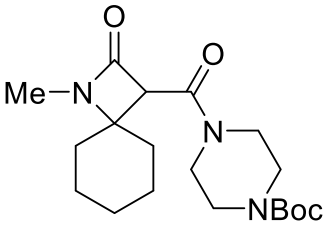


*(**3v**).* Obtained according to GP3 from acid **4a** and *N*-Boc-piperazine (59 mg, 0.32 mmol, 1.05 equiv). Column chromatography was carried out on silica gel, eluent: *n*-hexane/acetone, from 3 to 50% of acetone. Yield: 105 mg (95%). White solid, mp 123.4–124.2 °C. $^{1}$H NMR (400 MHz, DMSO-*d*$_6$) δ 4.22 (s, 1H), 3.77 – 3.56 (m, 2H), 3.51 – 3.17 (m, 5H), 2.63 (s, 3H), 2.02 – 1.91 (m, 1H), 1.82 – 1.45 (m, 6H), 1.41 (s, 9H), 1.36 – 1.21 (m, 3H), 1.10 – 0.92 (m, 1H). $^{13}$C NMR (101 MHz, DMSO-*d*$_6$) δ 165.1, 163.1, 153.7, 130.3, 128.4, 127.0, 79.2, 61.4, 58.4, 45.5, 41.0, 34.3, 28.5, 28.0, 24.1, 23.9, 23.6, 23.2. HRMS (ESI) *m/z*: [M+Na]$^+$ Calc. for $C_{19}H_{31}N_3NaO_4$ 388.2207; Found 388.2212.

## 3. NMR data

3.1. NMR spectra of β-lactams **3a–v**.

Copies of $^{1}H$ (400.13 MHz, $CDCl_3$) and $^{13}C\{^{1}H\}$ (100.61 MHz, $CDCl_3$) spectra of **3a**

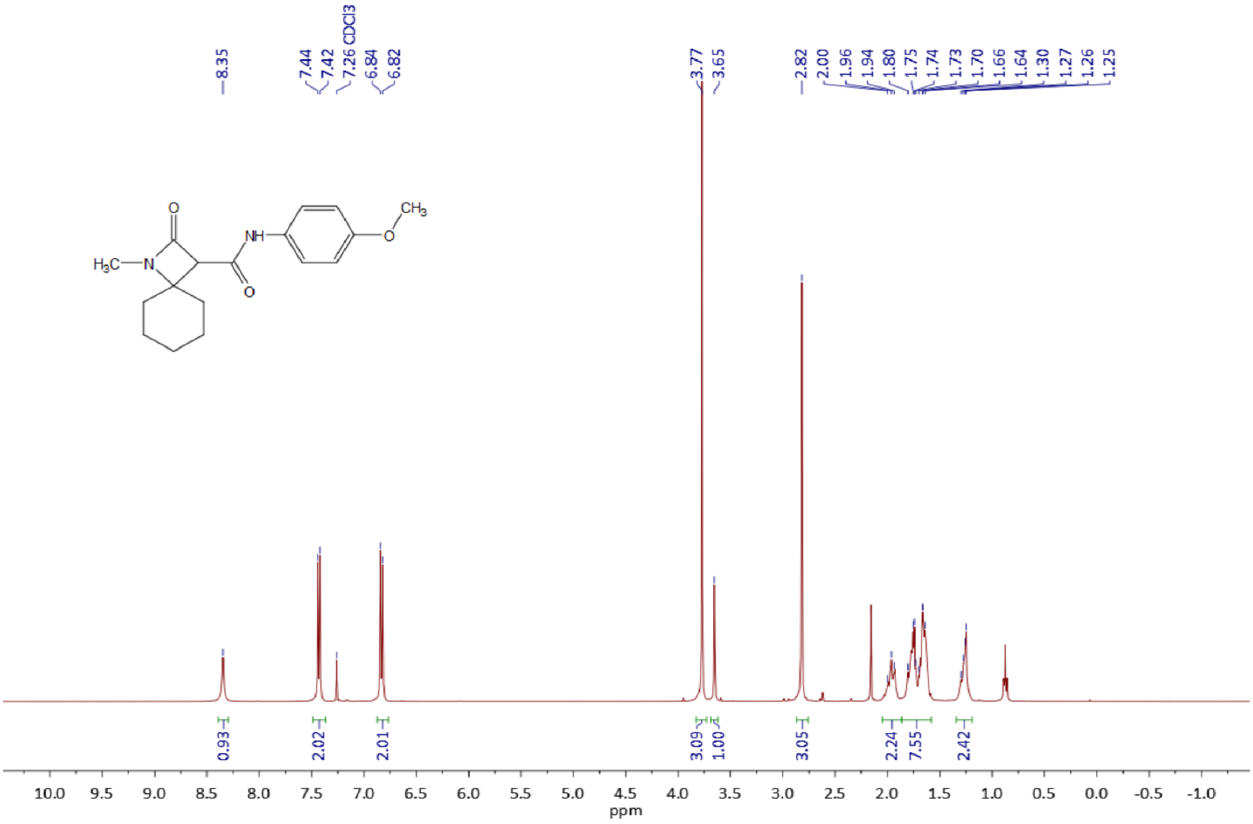


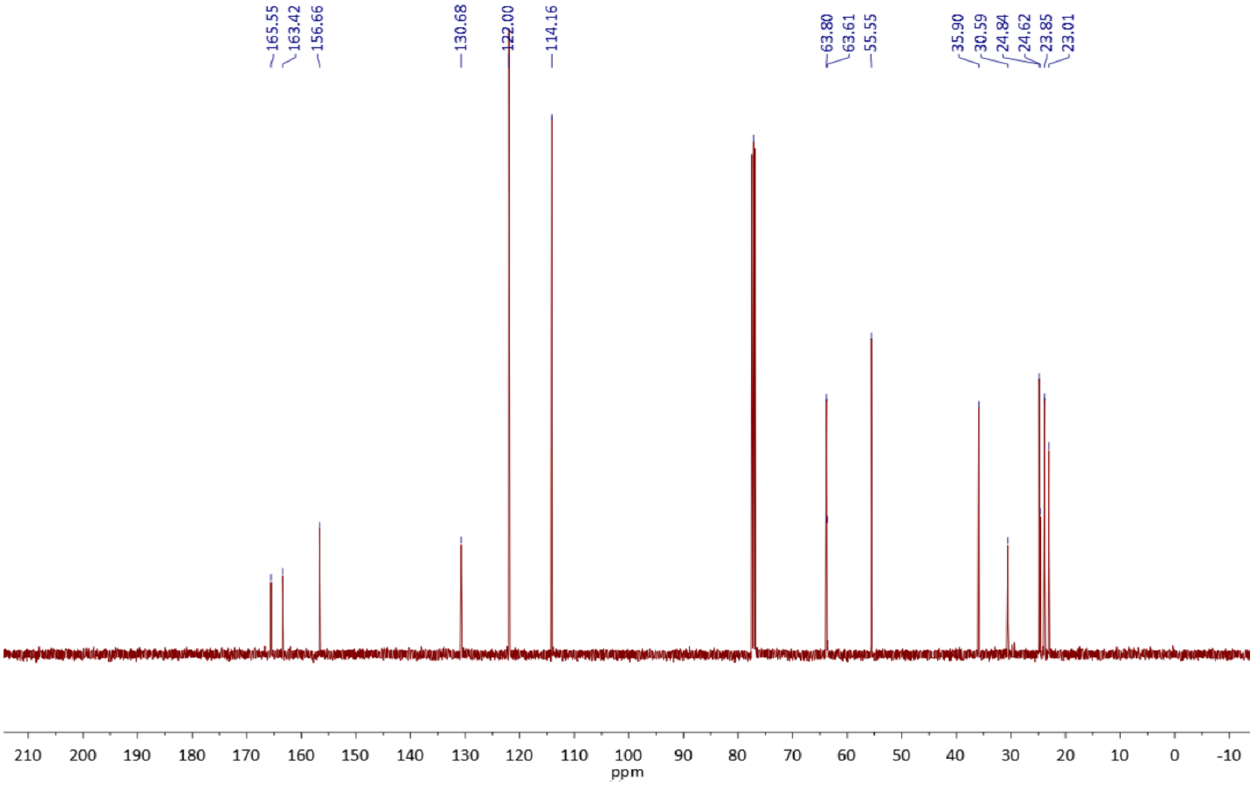

Copies of $^{1}H$ (400.13 MHz, $CDCl_3$) and $^{13}C\{^{1}H\}$ (100.61 MHz, $CDCl_3$) spectra of **3b**

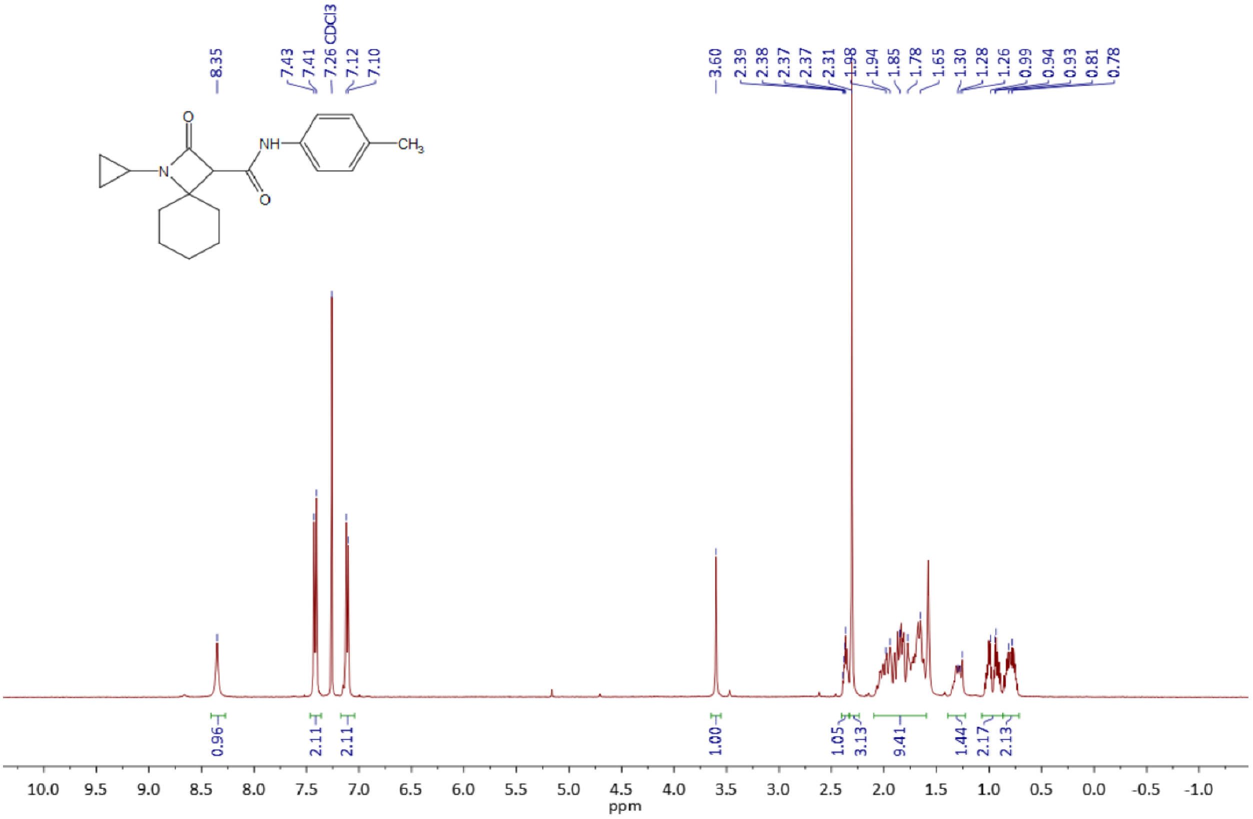


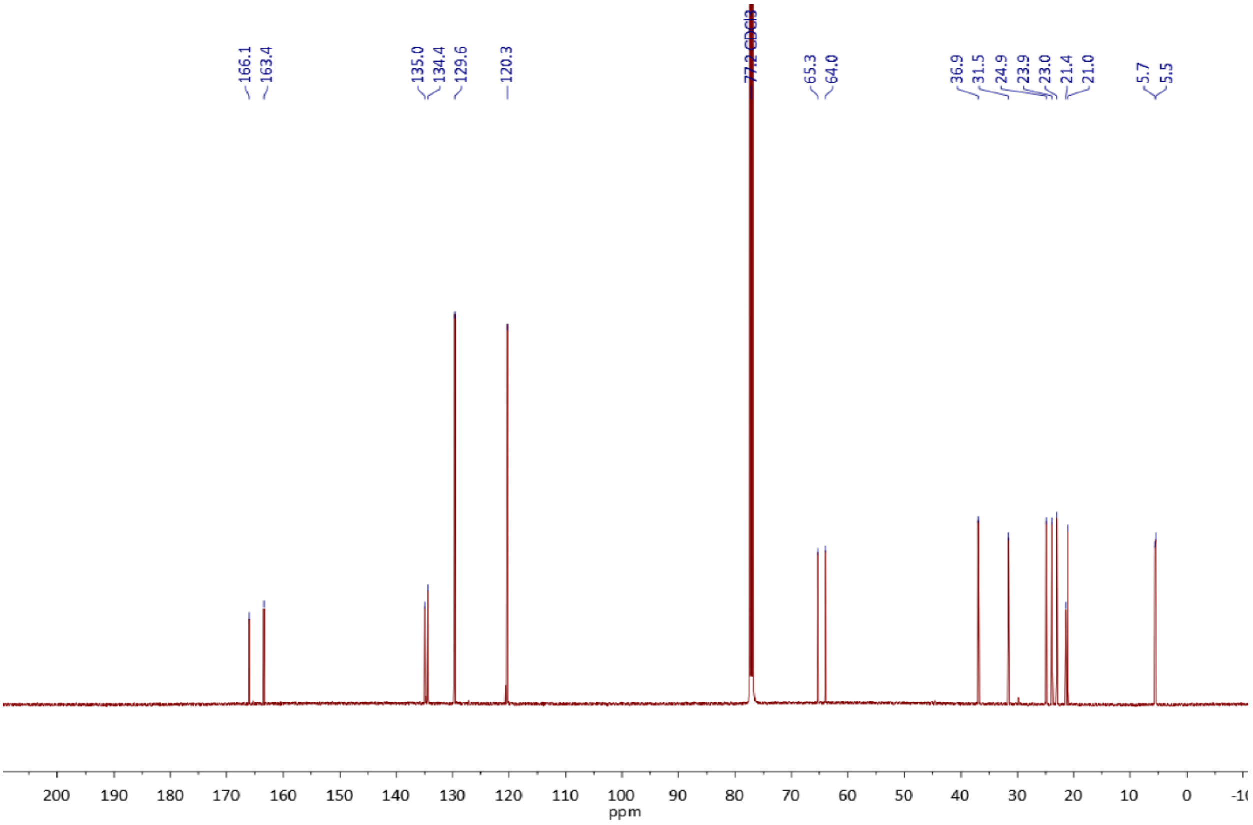

Copies of $^1$H (400.13 MHz, $CDCl_3$) and $^{13}C\{^1H\}$ (100.61 MHz, $CDCl_3$) spectra of **3c**

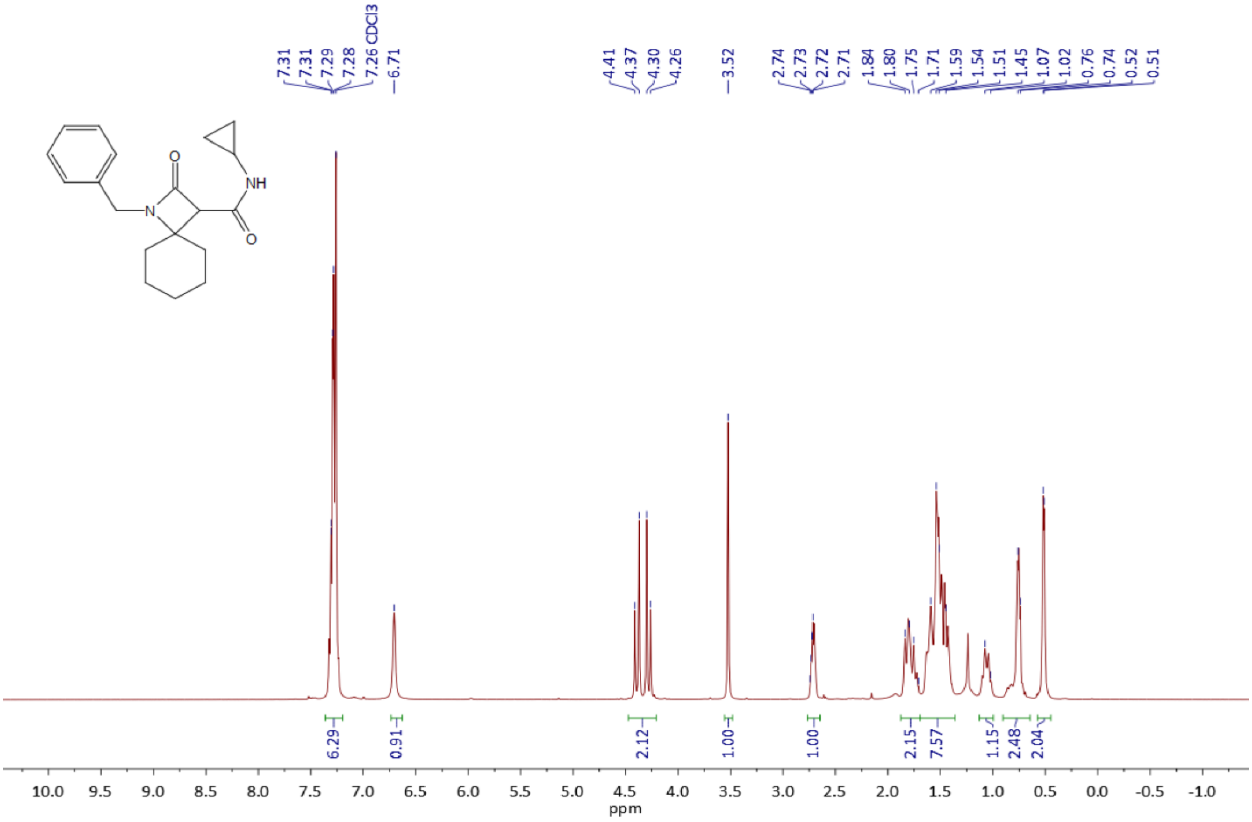


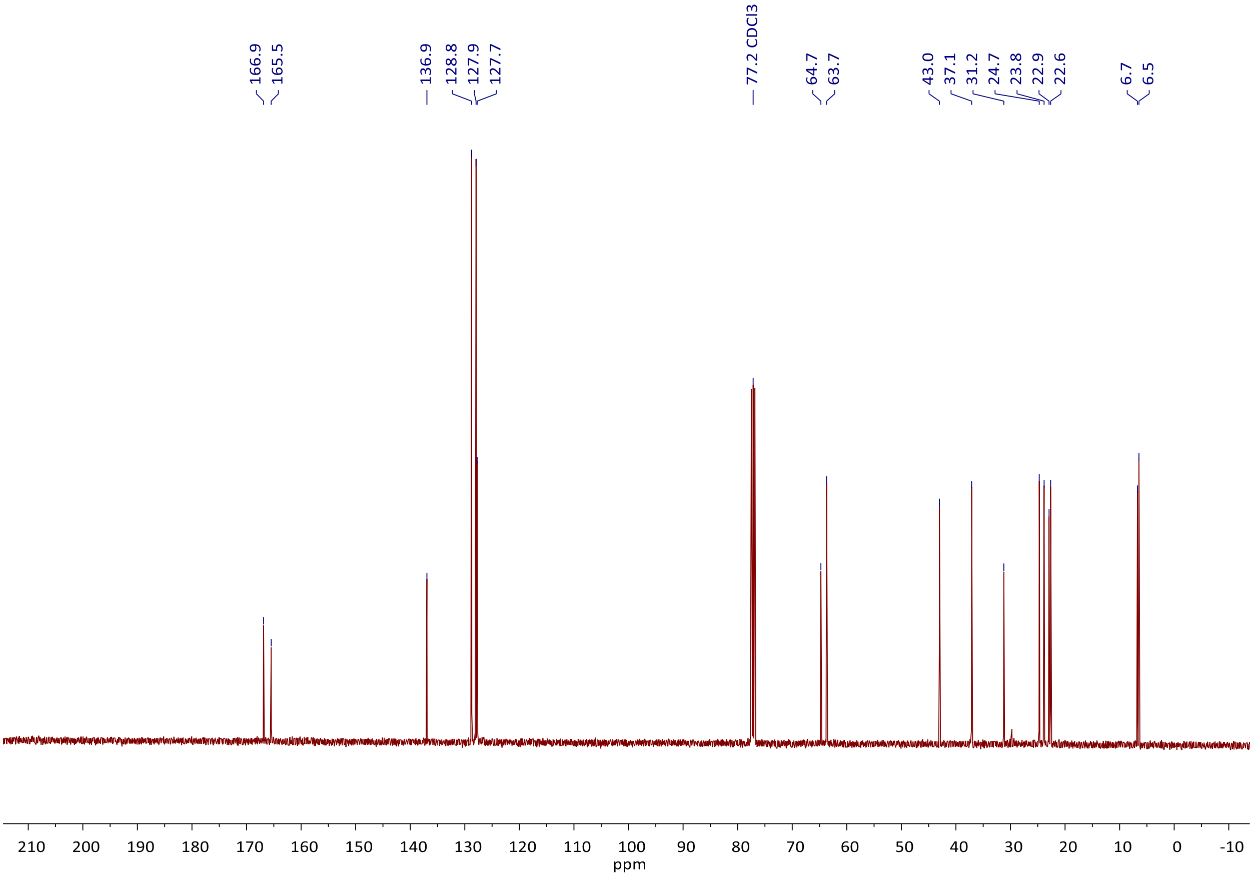

Copies of $^{1}H$ (400.13 MHz, $CDCl_3$) and $^{13}C\{^{1}H\}$ (100.61 MHz, $CDCl_3$) spectra of **3d**

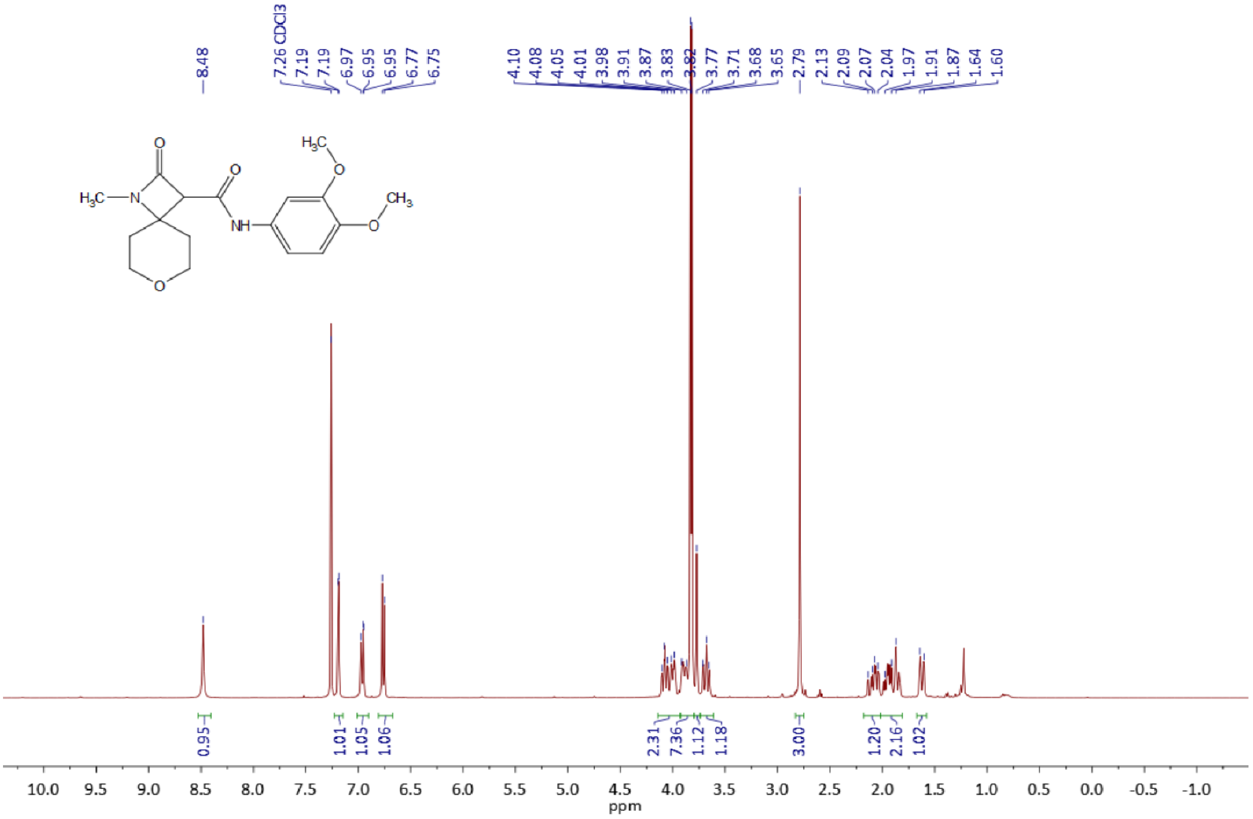


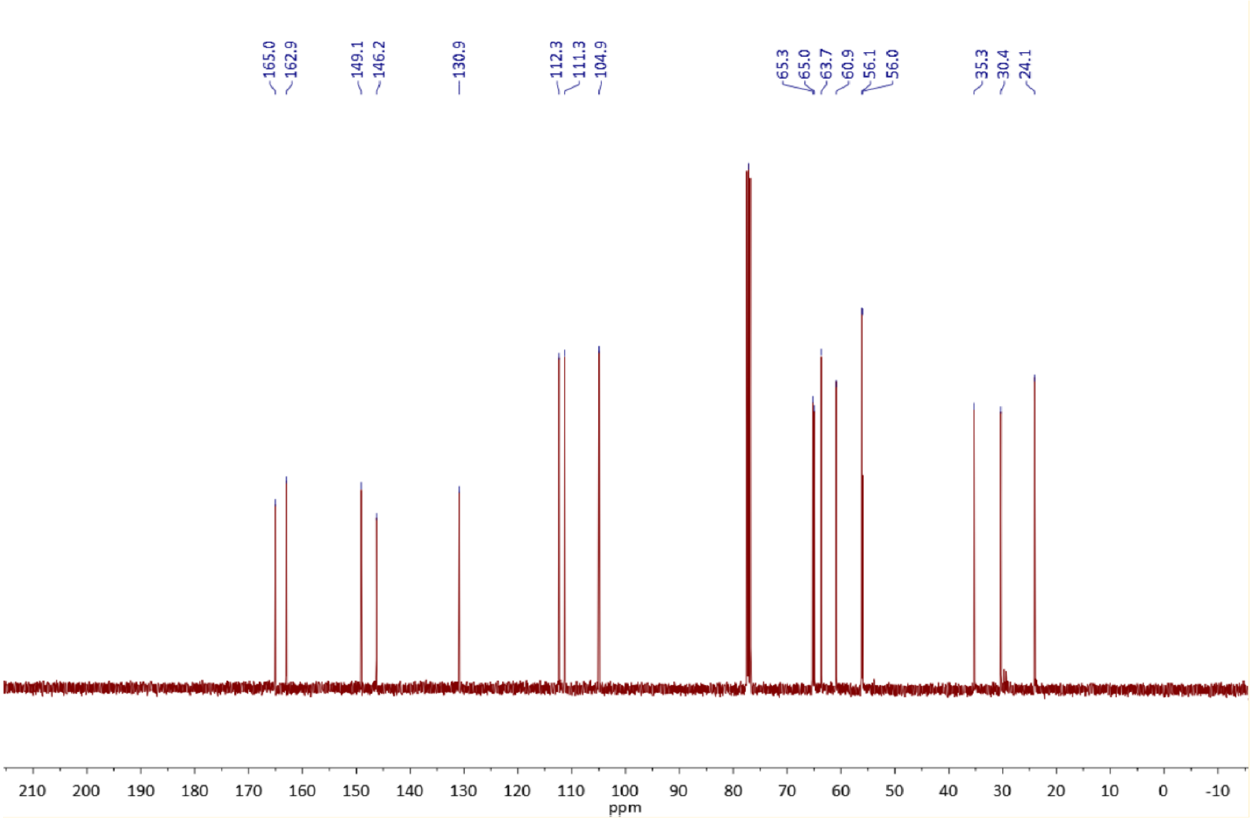

Copies of $^{1}H$ (400.13 MHz, $CDCl_3$) and $^{13}C\{^{1}H\}$ (100.61 MHz, $CDCl_3$) spectra of **3e**

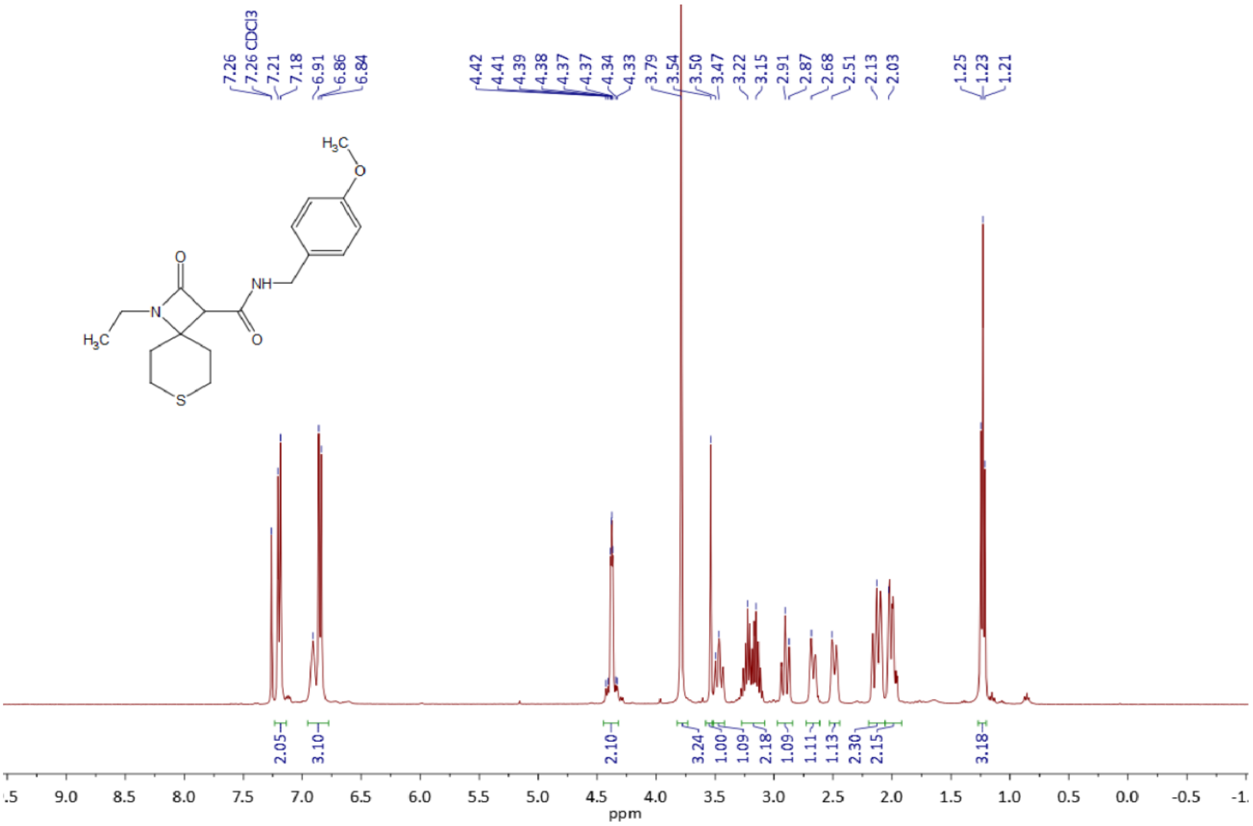


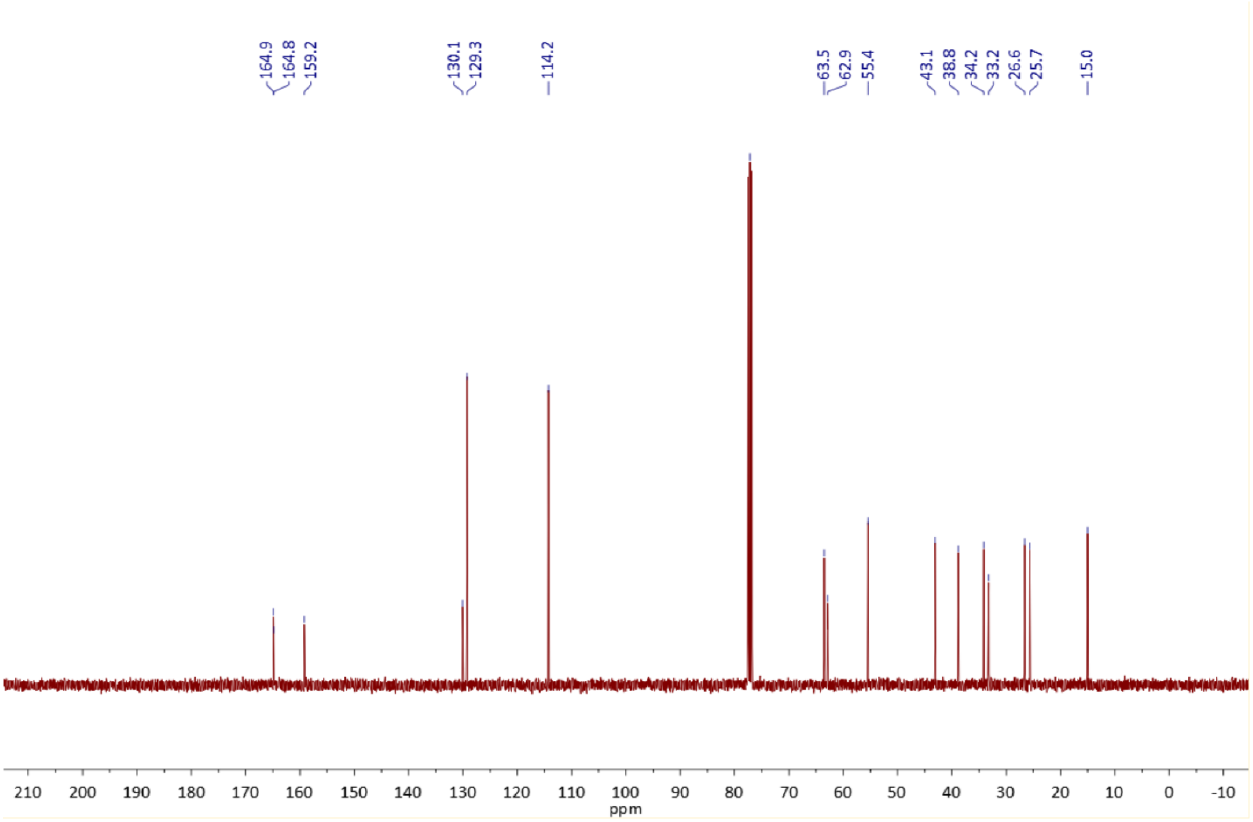

Copies of $^{1}H$ (400.13 MHz, $CDCl_3$) and $^{13}C\{^{1}H\}$ (100.61 MHz, $CDCl_3$) spectra of **3f**

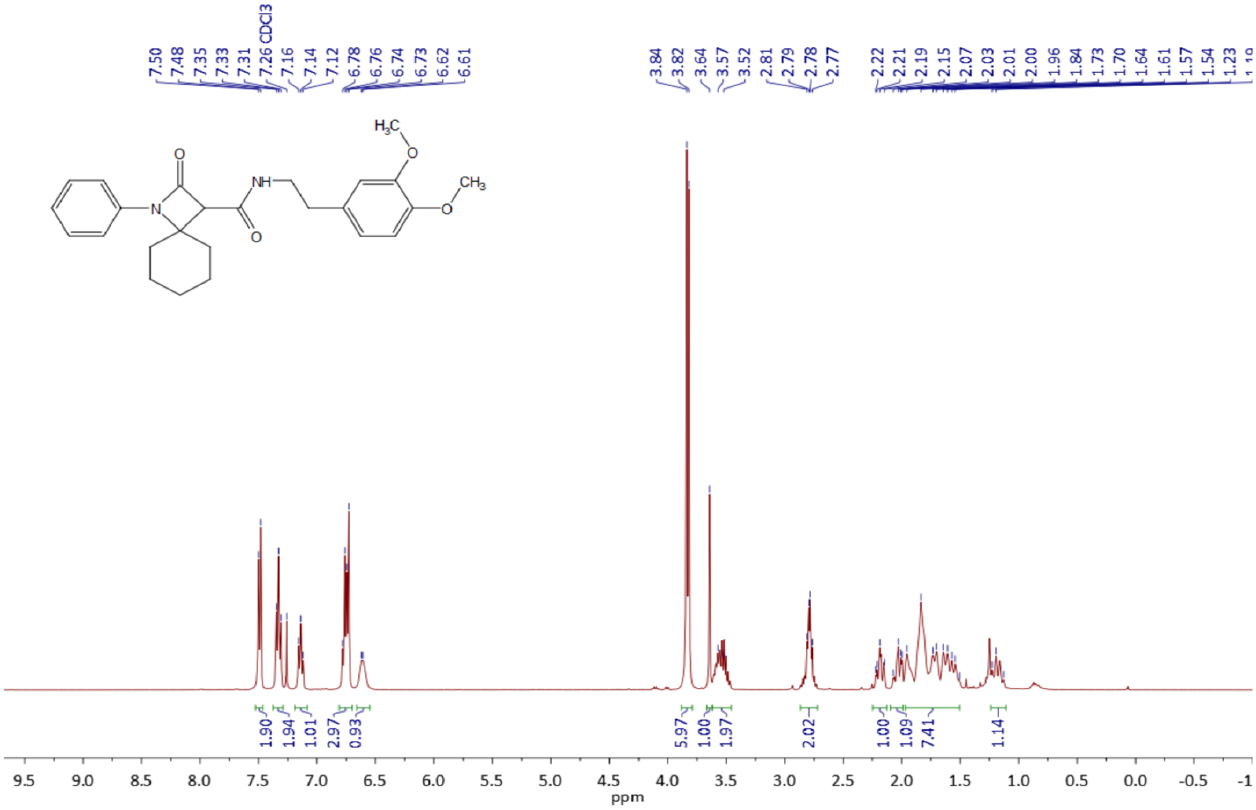


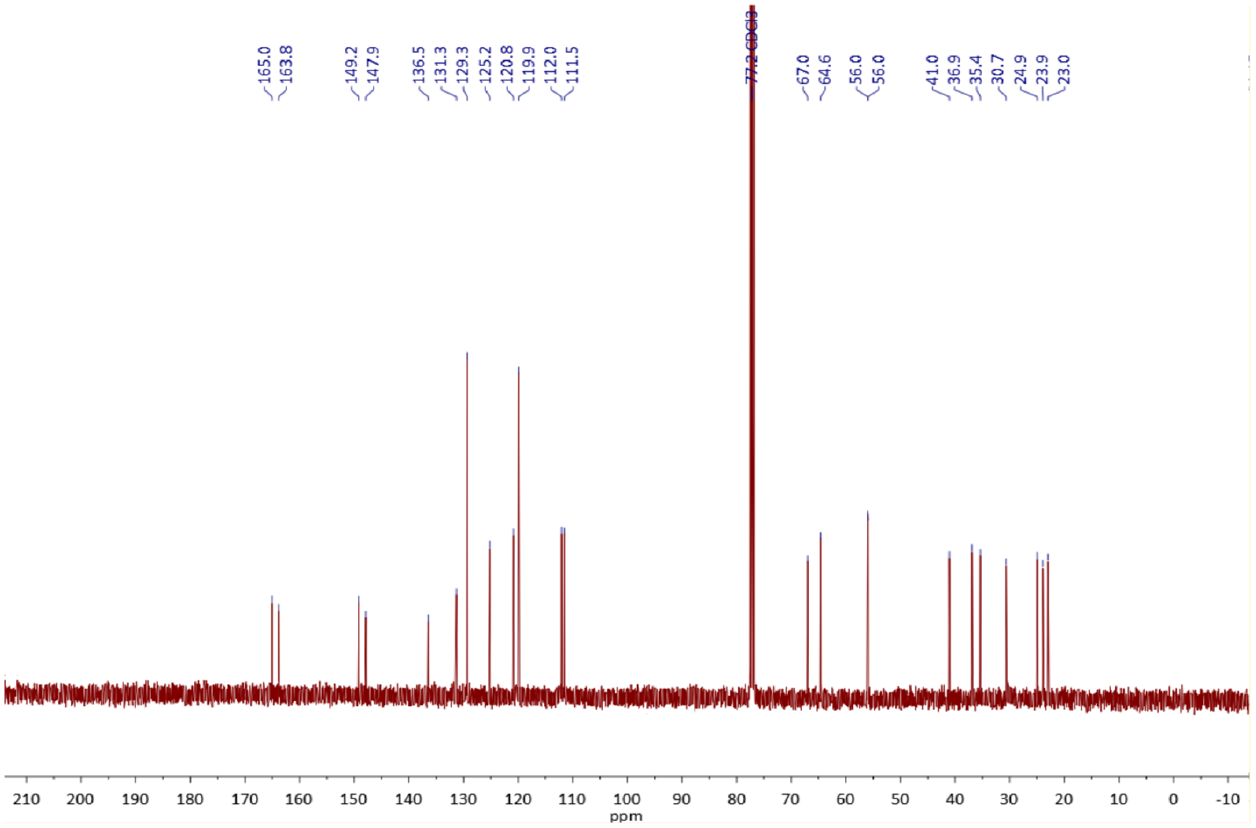

Copies of $^{1}$H (400.13 MHz, $CDCl_3$) and $^{13}$C{$^{1}$H} (100.61 MHz, $CDCl_3$) spectra of **3g**

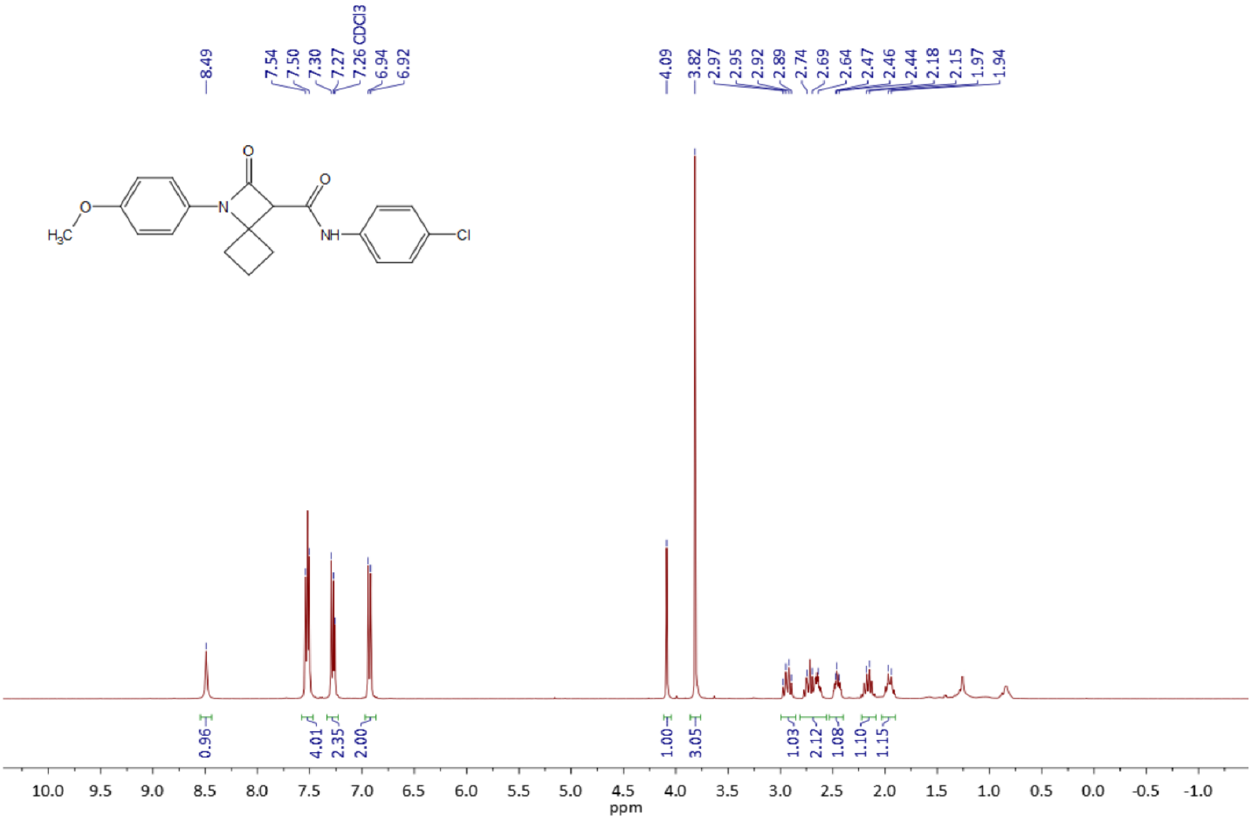


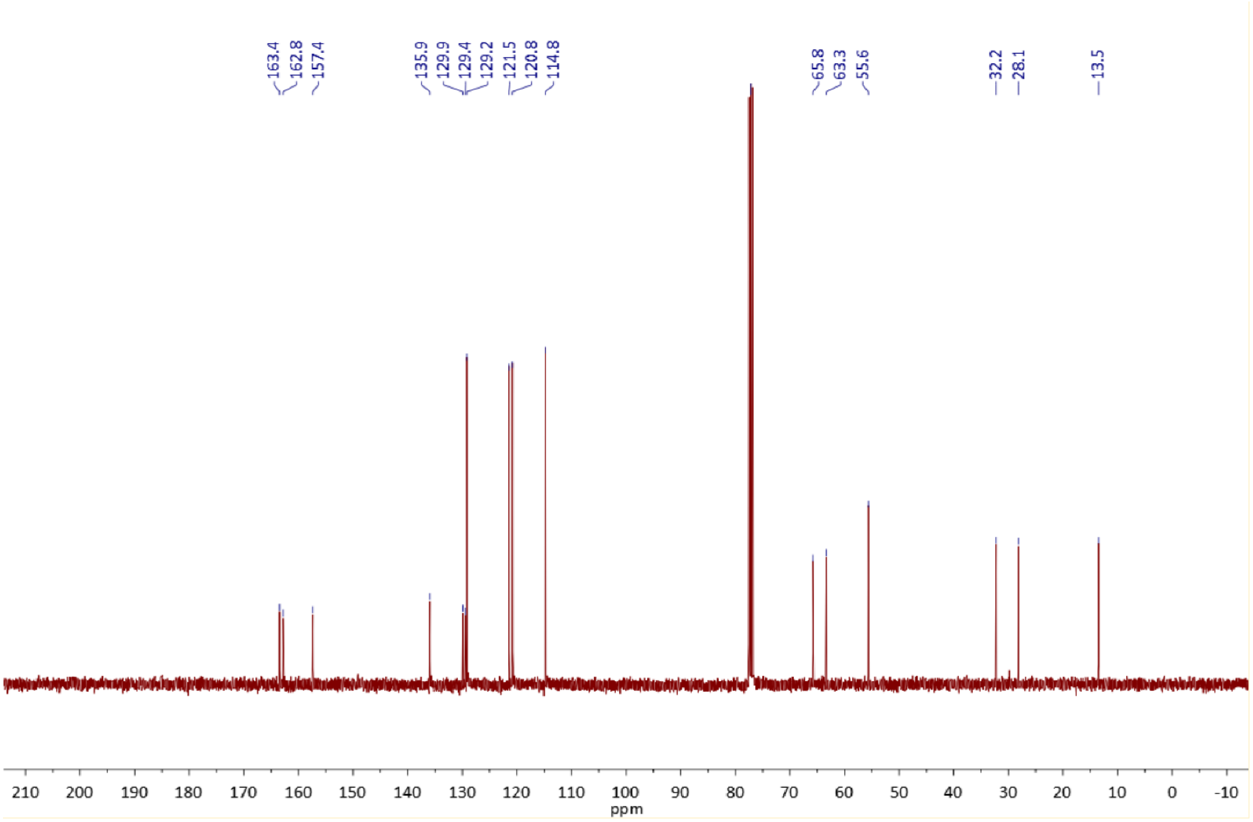

Copies of $^{1}H$ (400.13 MHz, $CDCl_3$), $^{13}C\{^{1}H\}$ (100.61 MHz, $CDCl_3$) and $^{19}F\{^{1}H\}$ (376.50 MHz, $CDCl_3$) spectra of **3h**

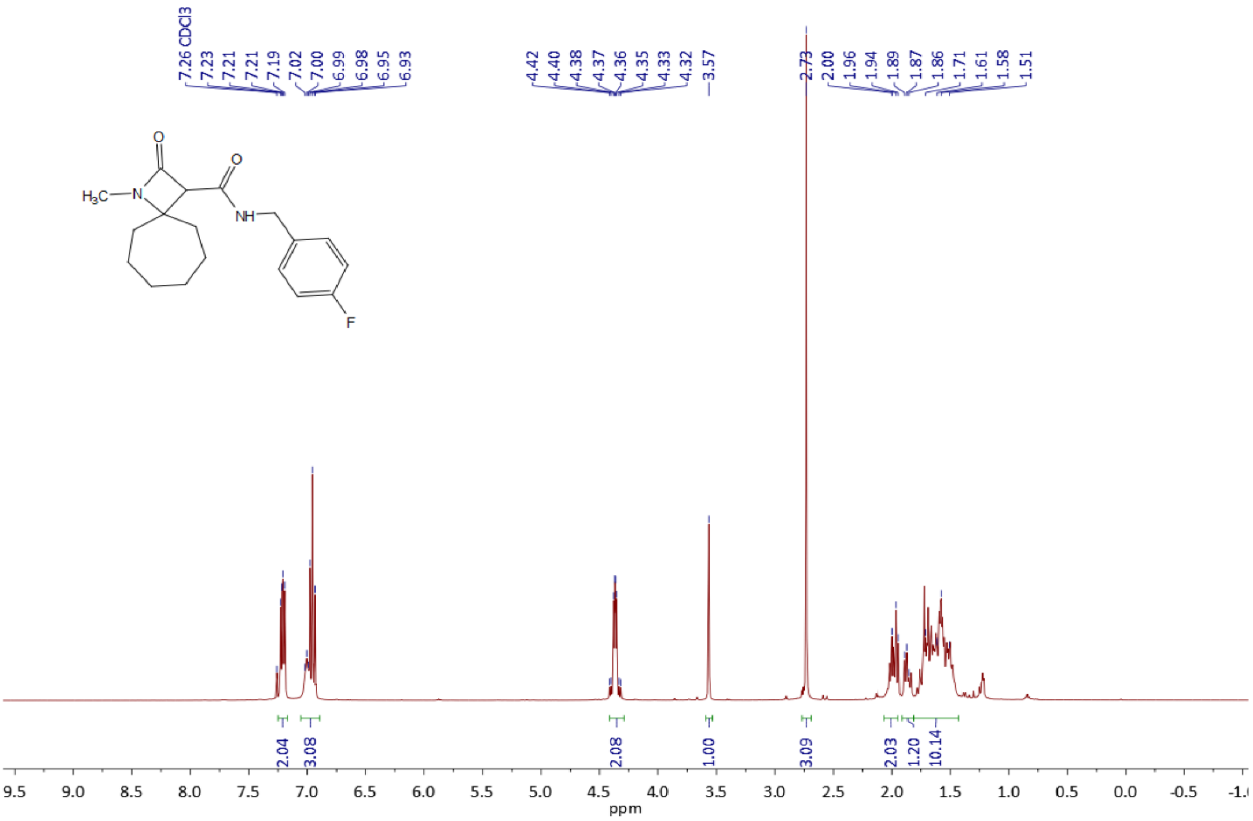


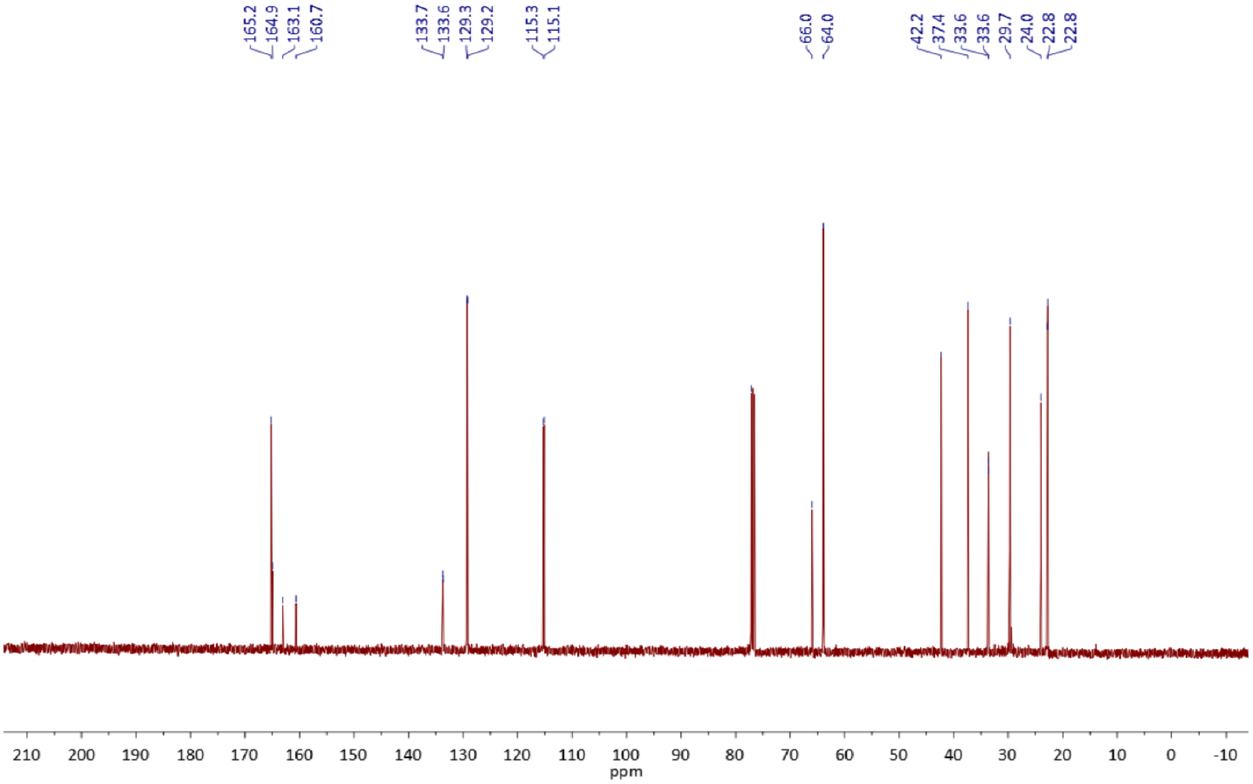

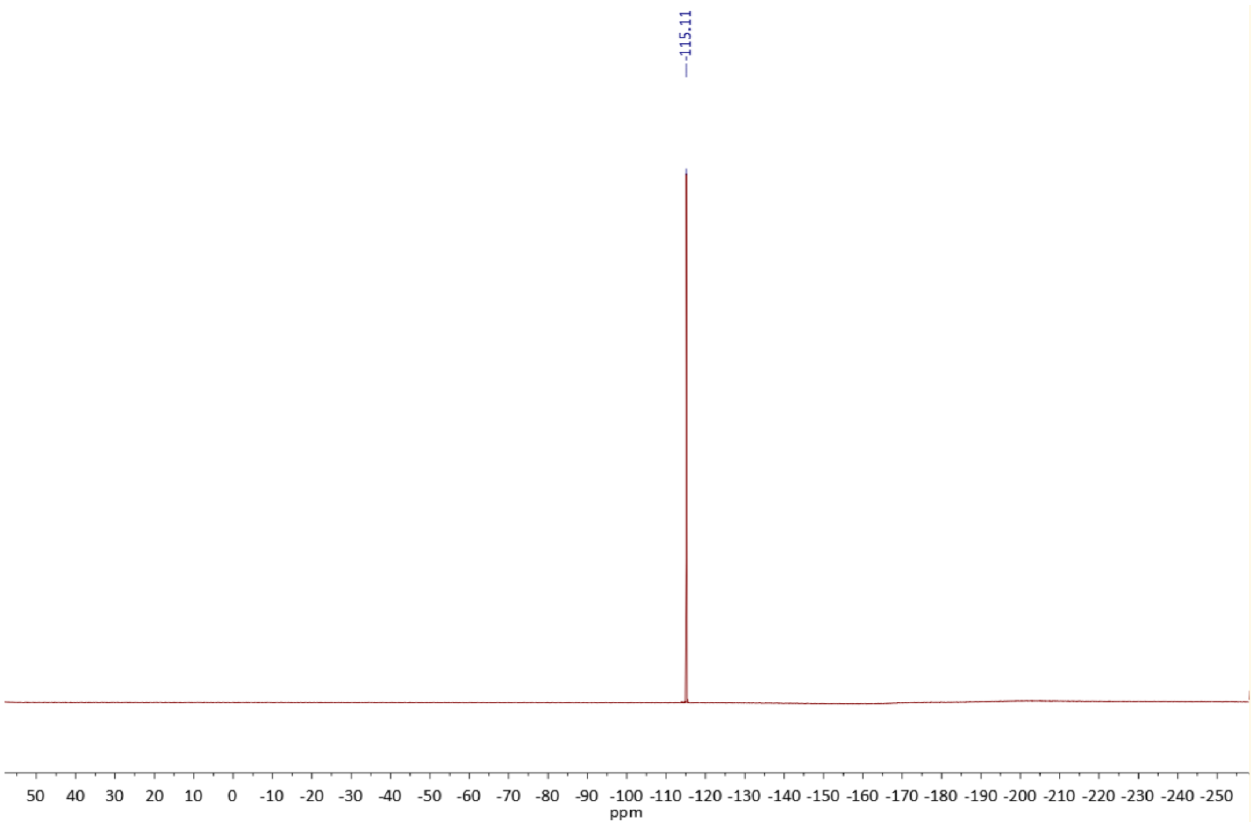
-115.11
50
40
30
20
10
0
-10
-20
-30
-40
-50
-60
-70
-80
-90
-100
-110
-120
-130
-140
-150
-160
-170
-180
-190
-200
-210
-220
-230
-240
-250
ppm

Copies of $^1$H (400.13 MHz, DMSO-$d_6$), $^{13}$C{$^1$H} (100.61 MHz, DMSO-$d_6$), and $^{19}$F{$^1$H} (376.50 MHz, DMSO-$d_6$) spectra of **3i**

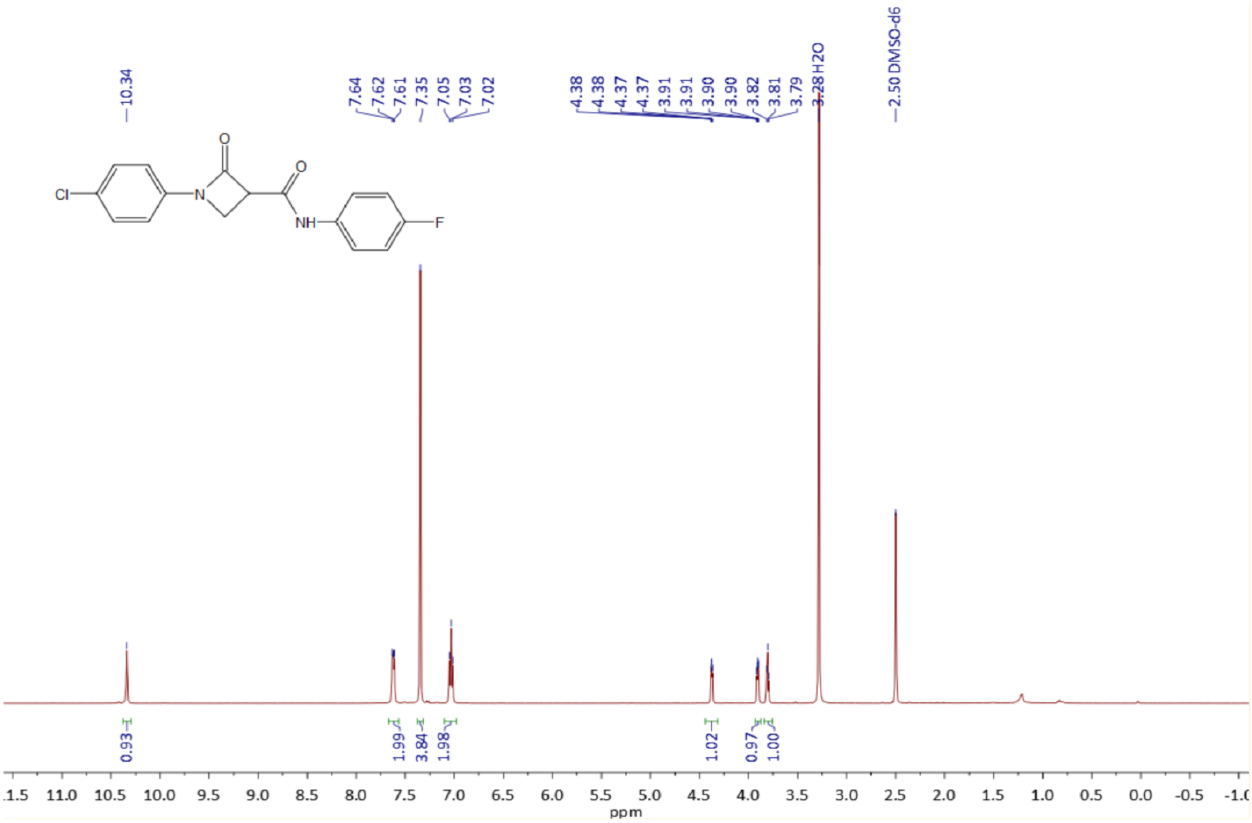


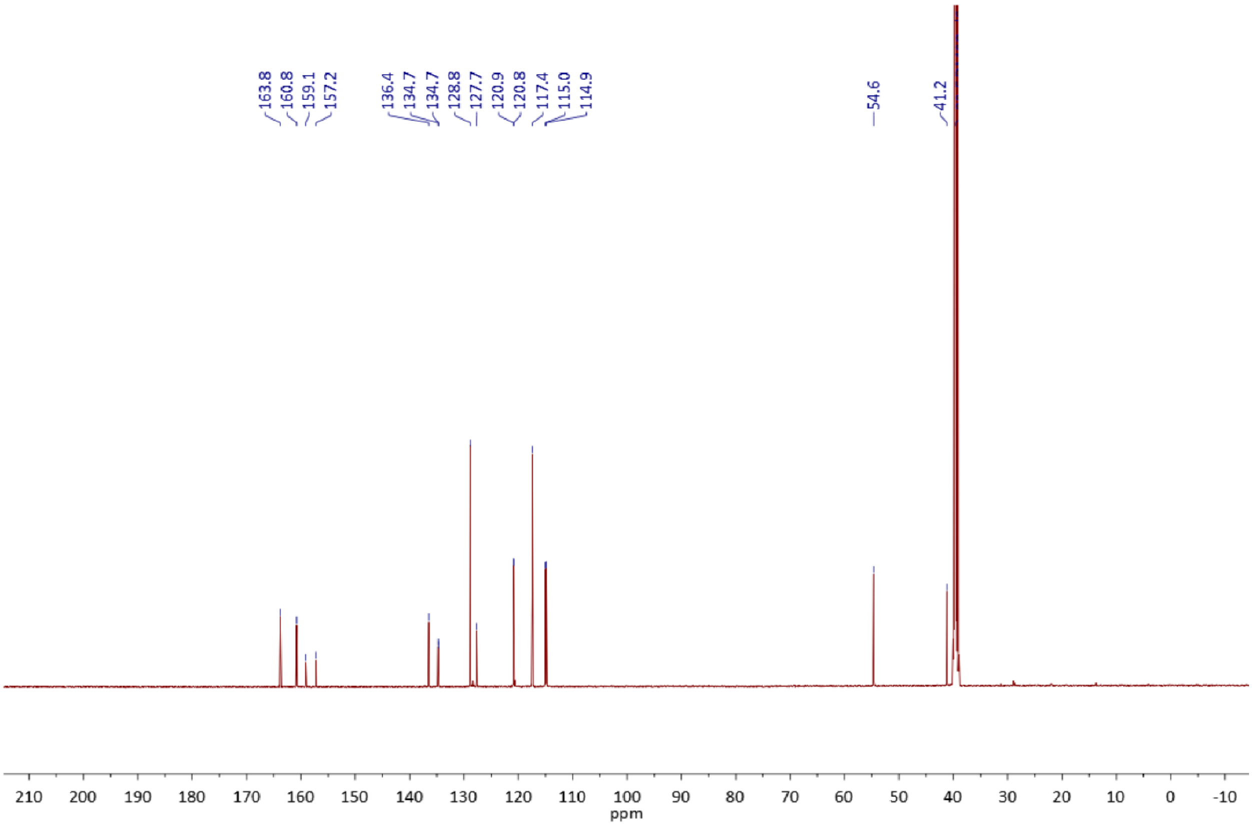

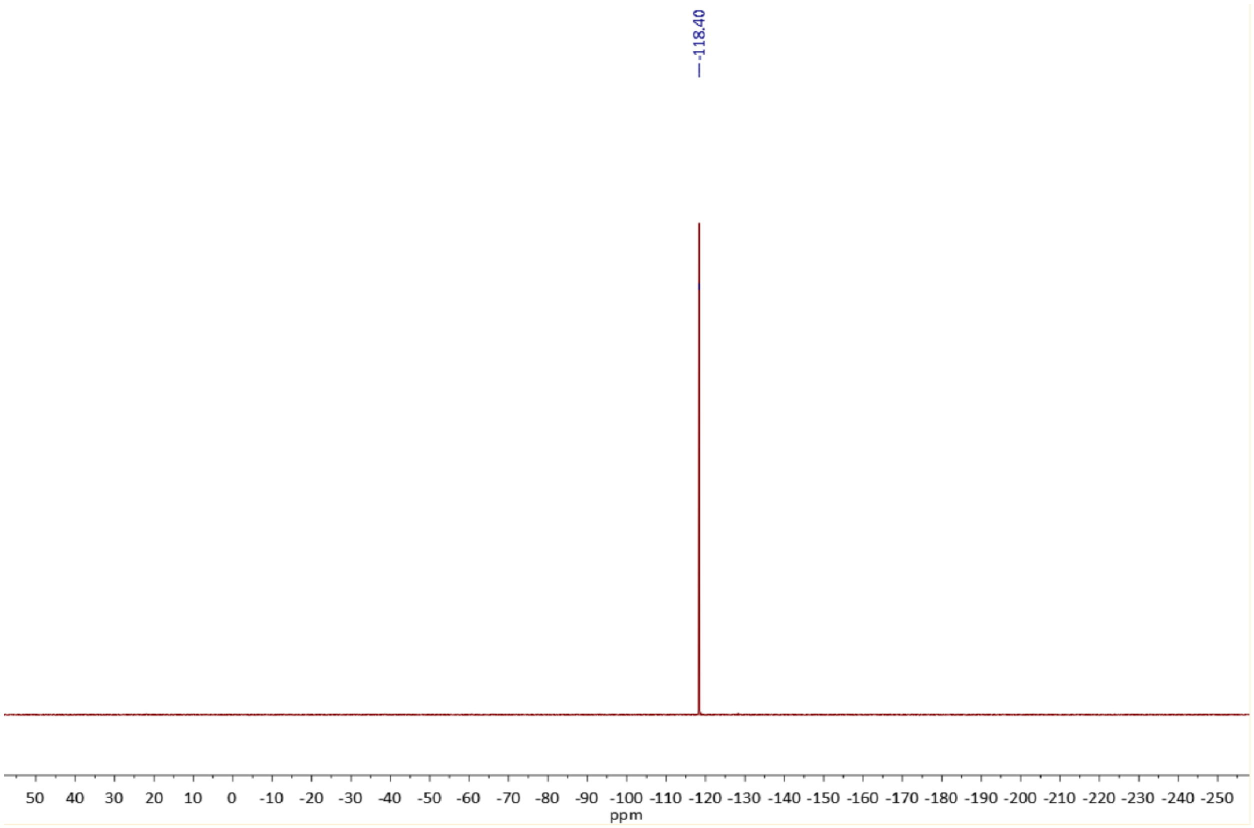
-118.40
50 40 30 20 10 0 -10 -20 -30 -40 -50 -60 -70 -80 -90 -100 -110 -120 -130 -140 -150 -160 -170 -180 -190 -200 -210 -220 -230 -240 -250
ppm

Copies of $^{1}H$ (400.13 MHz, DMSO-$d_6$), $^{13}C\{^{1}H\}$ (100.61 MHz, DMSO-$d_6$), and $^{19}F\{^{1}H\}$ (376.50 MHz, DMSO-$d_6$) spectra of **3j**

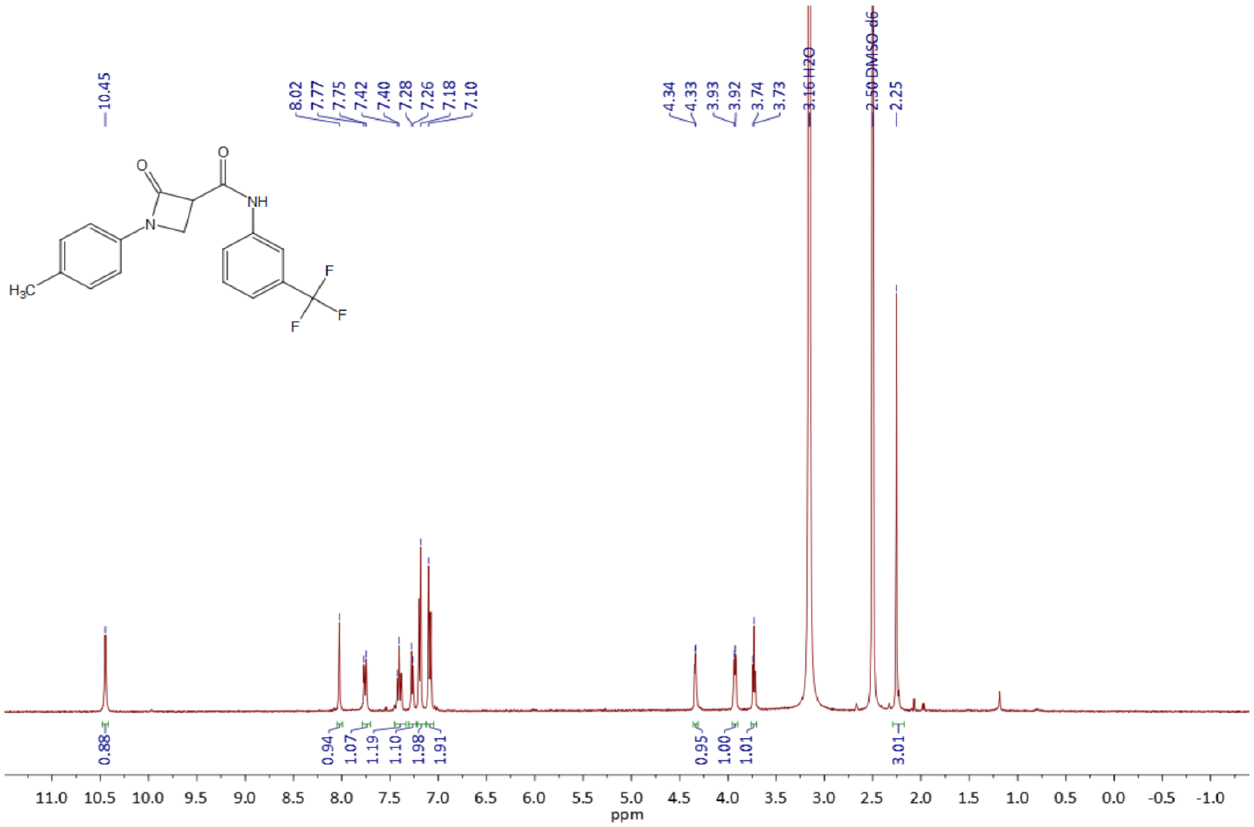


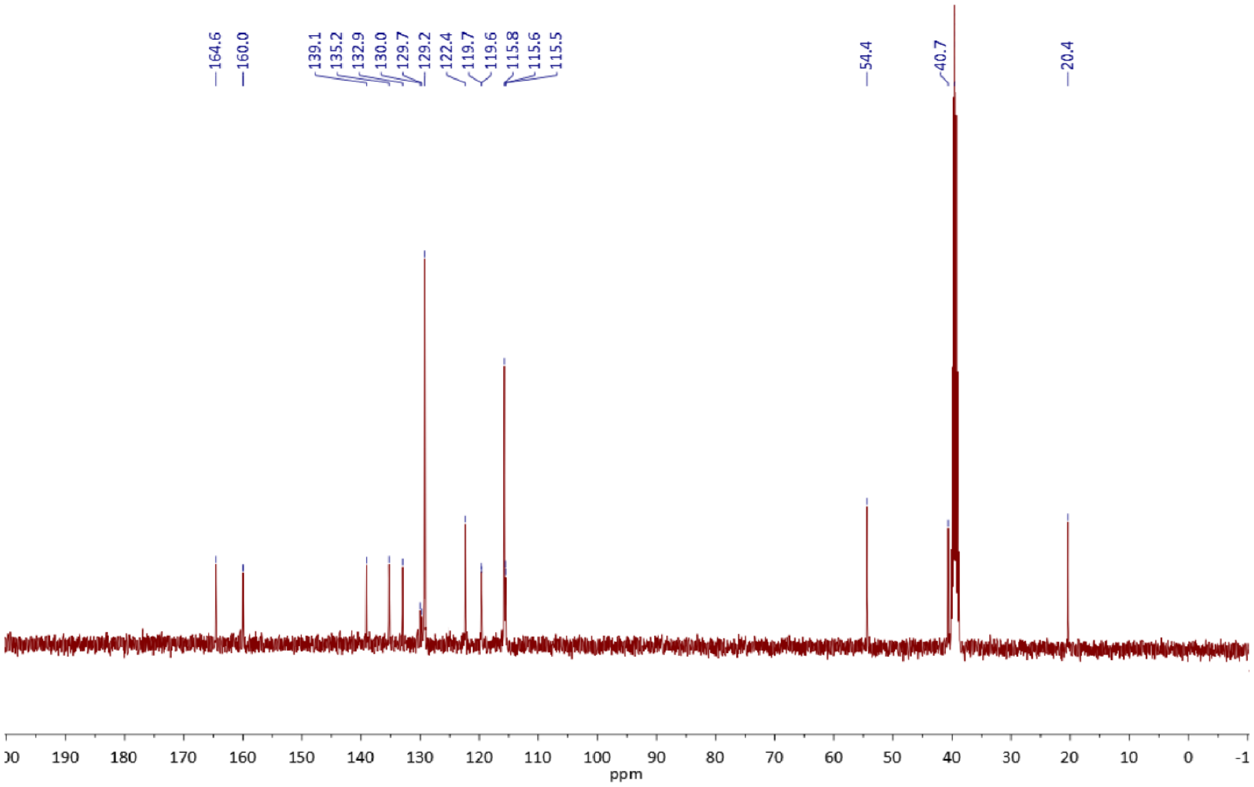

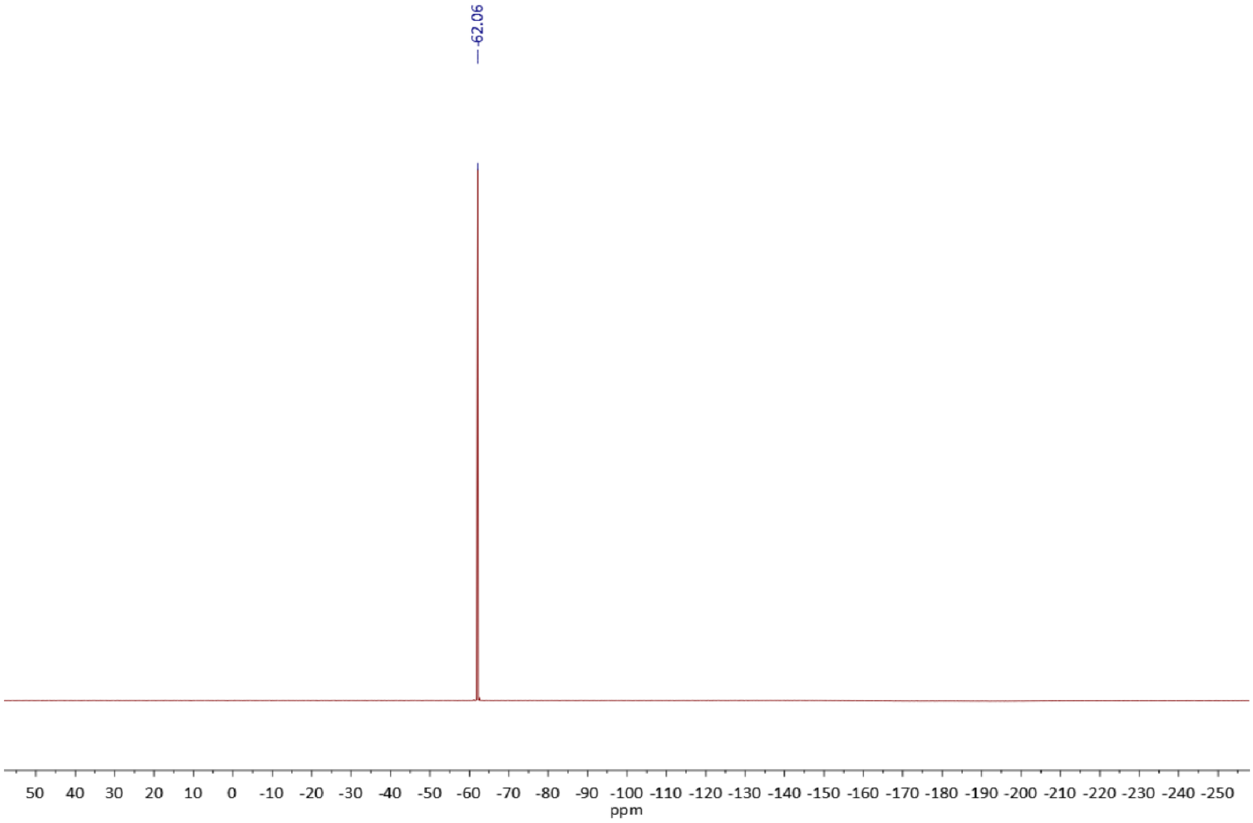
-62.06
50 40 30 20 10 0 -10 -20 -30 -40 -50 -60 -70 -80 -90 -100 -110 -120 -130 -140 -150 -160 -170 -180 -190 -200 -210 -220 -230 -240 -250
ppm

Copies of $^1$H (400.13 MHz, $CDCl_3$) and $^{13}C\{^1H\}$ (100.61 MHz, $CDCl_3$) spectra of **3k**

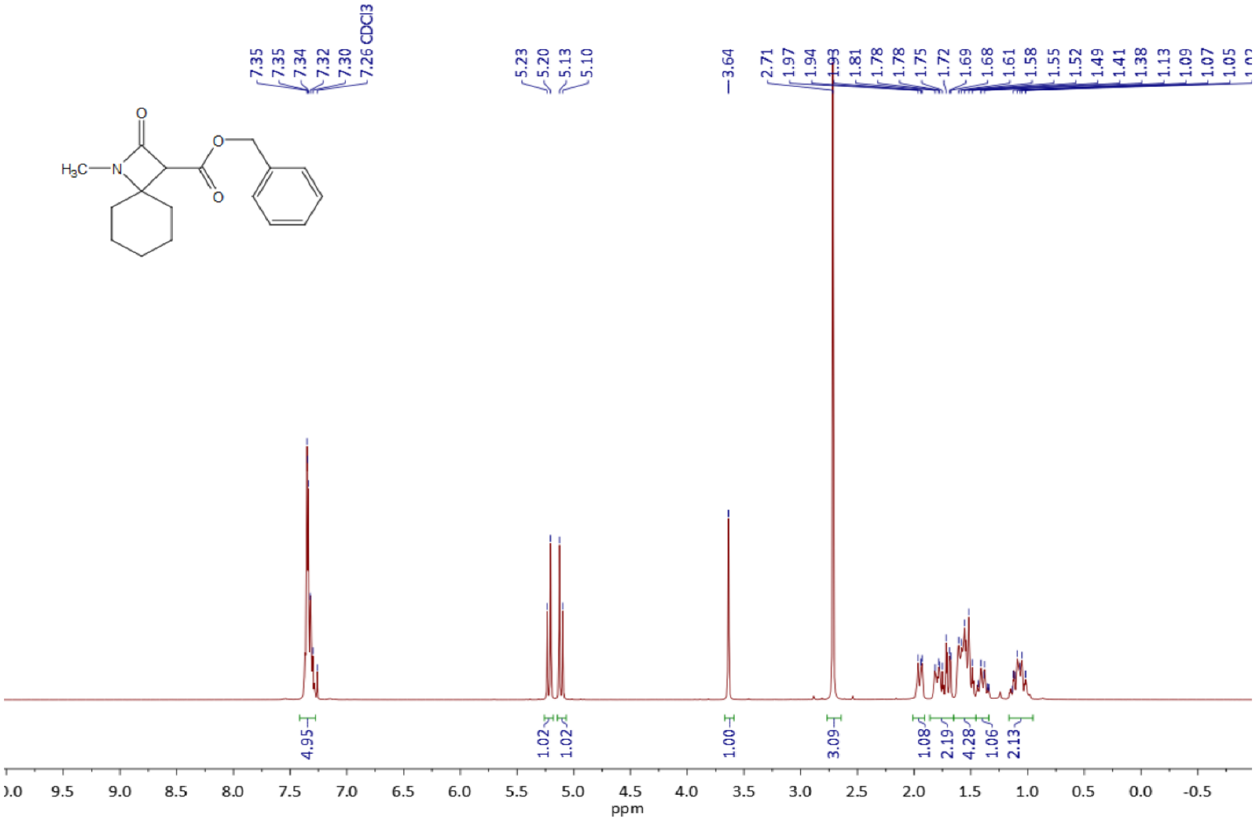

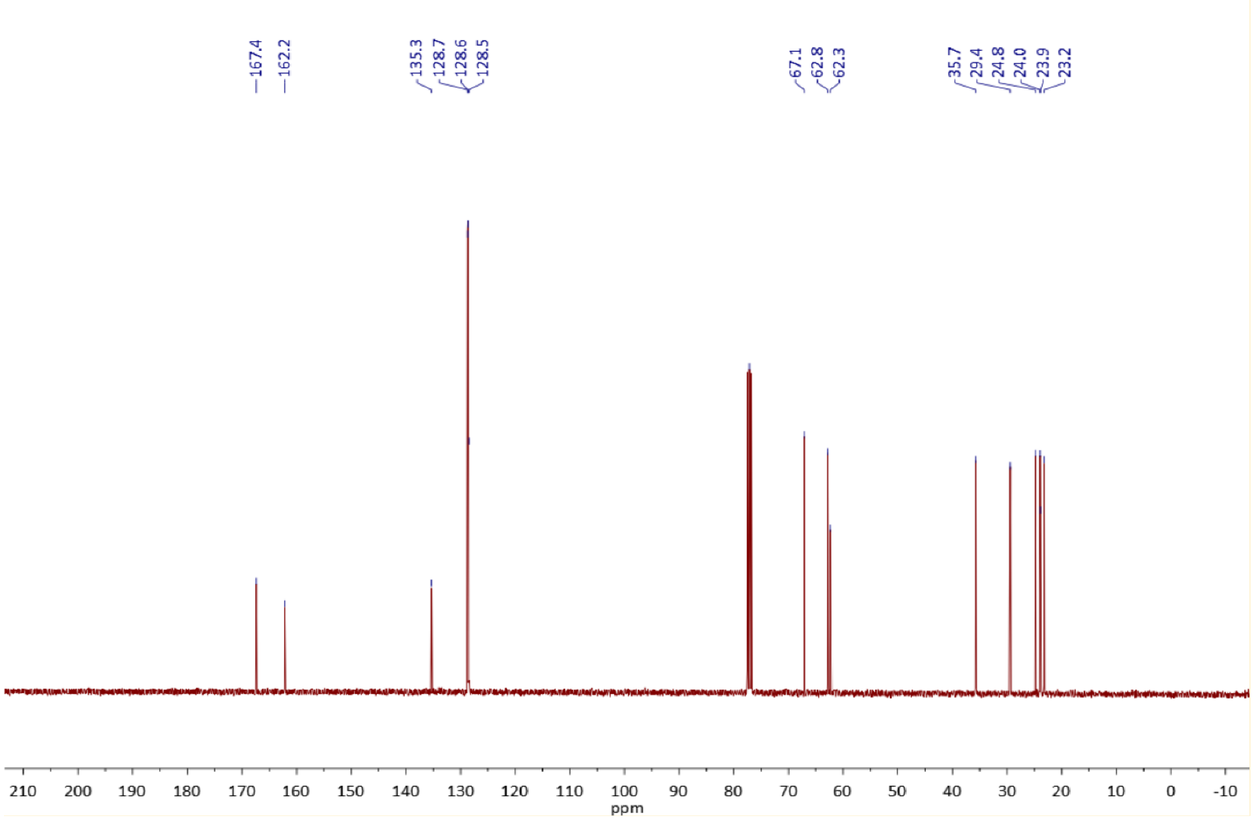

Copies of $^{1}H$ (400.13 MHz, $CDCl_3$) and $^{13}C\{^{1}H\}$ (100.61 MHz, $CDCl_3$) spectra of **3l**

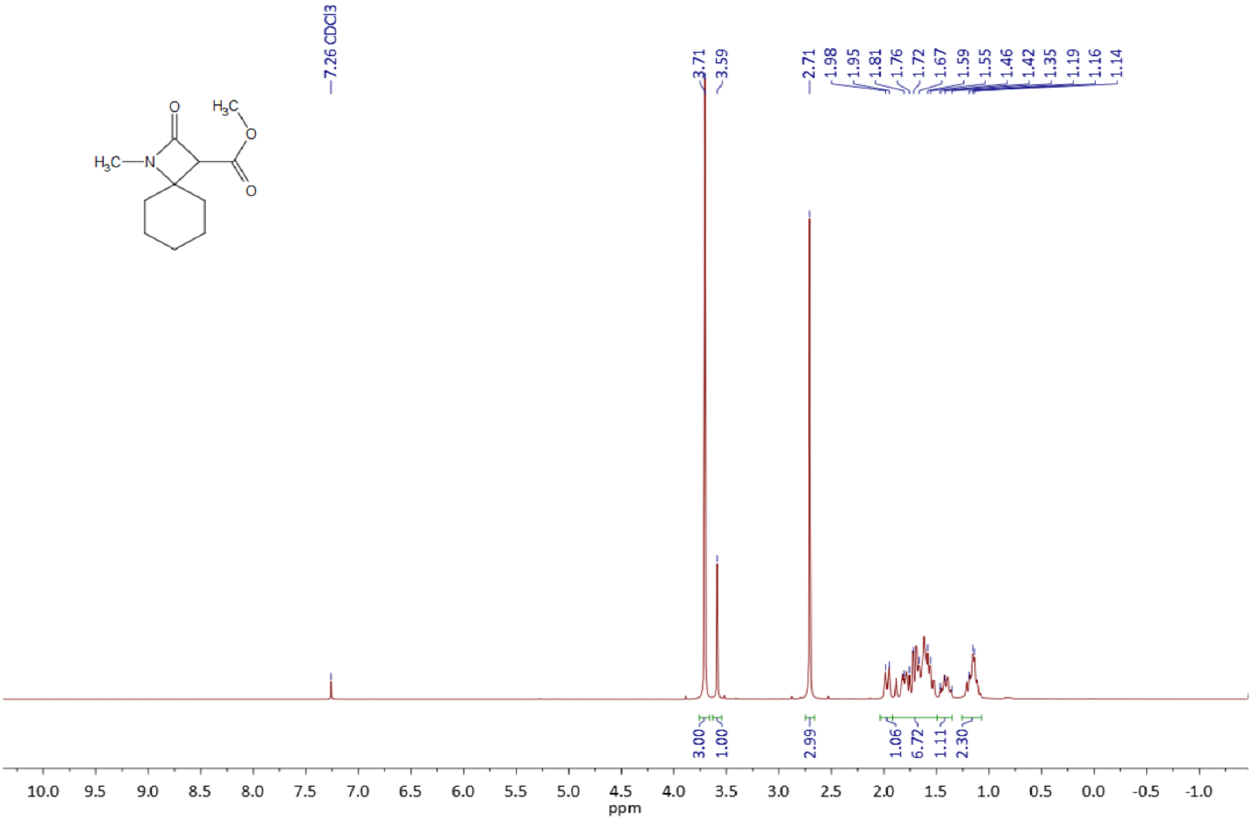


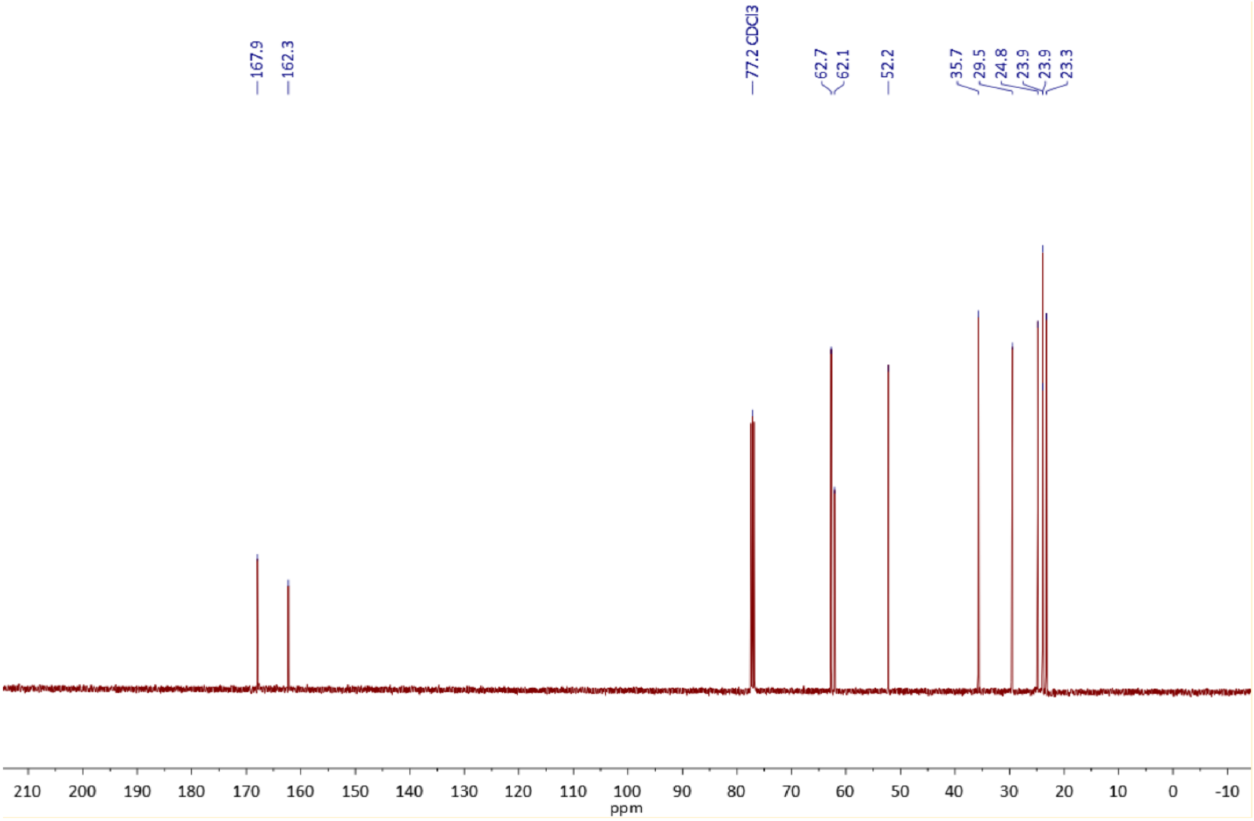

Copies of $^{1}H$ (400.13 MHz, $CDCl_3$) and $^{13}C\{^{1}H\}$ (100.61 MHz, $CDCl_3$) spectra of **3m**

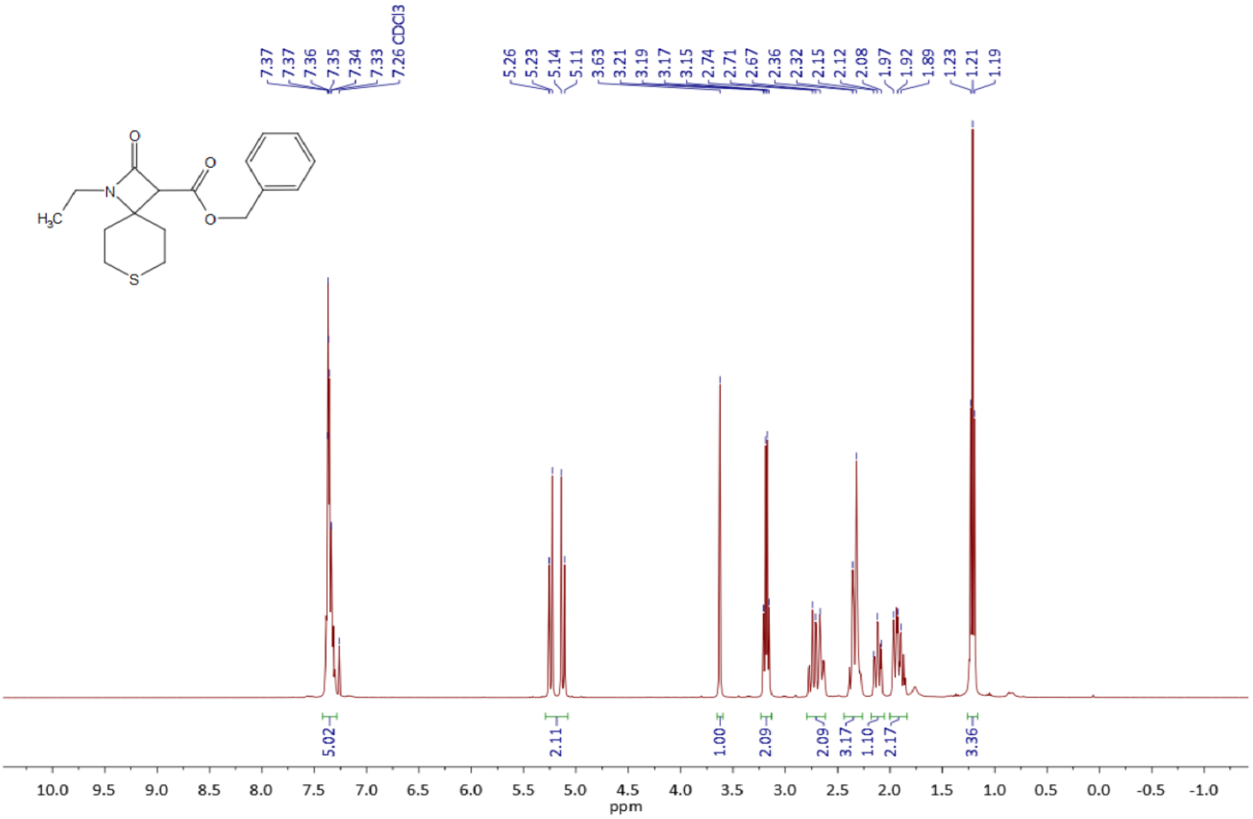


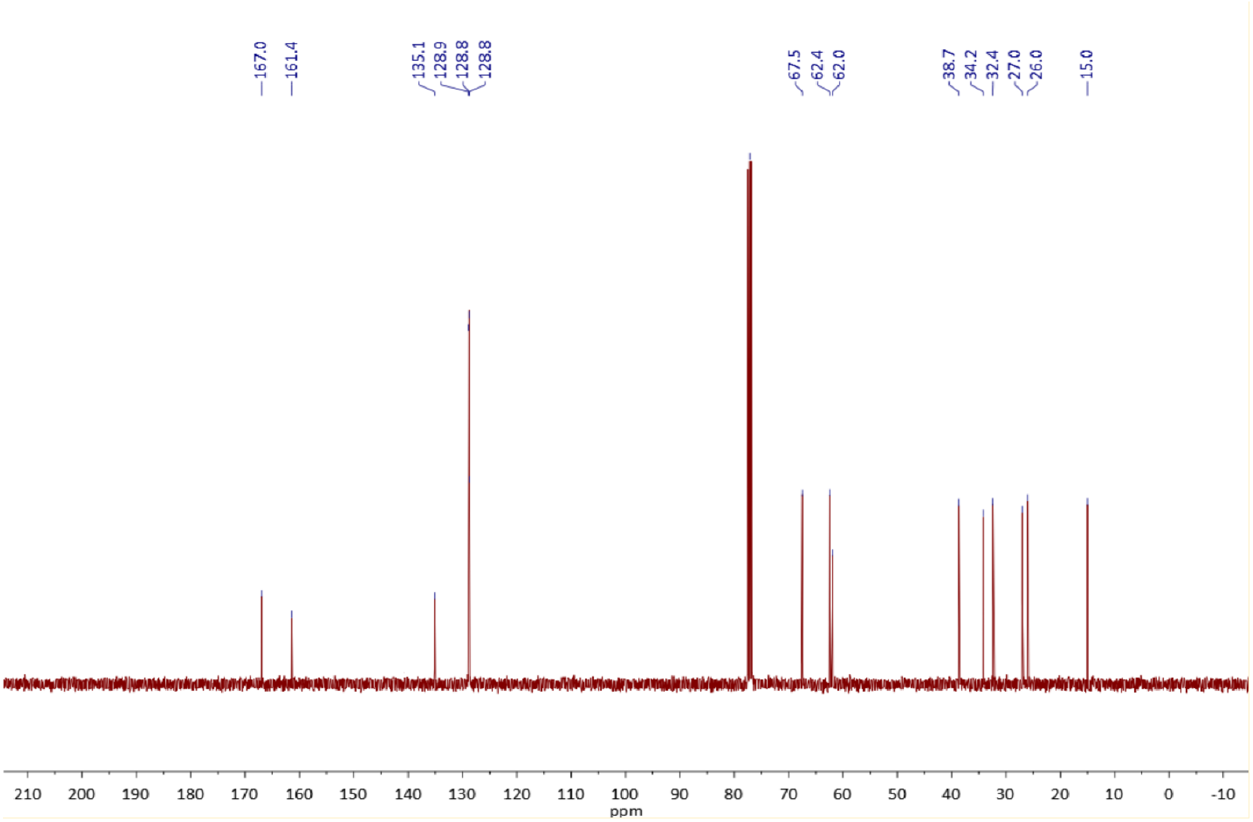

Copies of $^{1}H$ (400.13 MHz, $CDCl_3$) and $^{13}C\{^{1}H\}$ (100.61 MHz, $CDCl_3$) spectra of **3n**

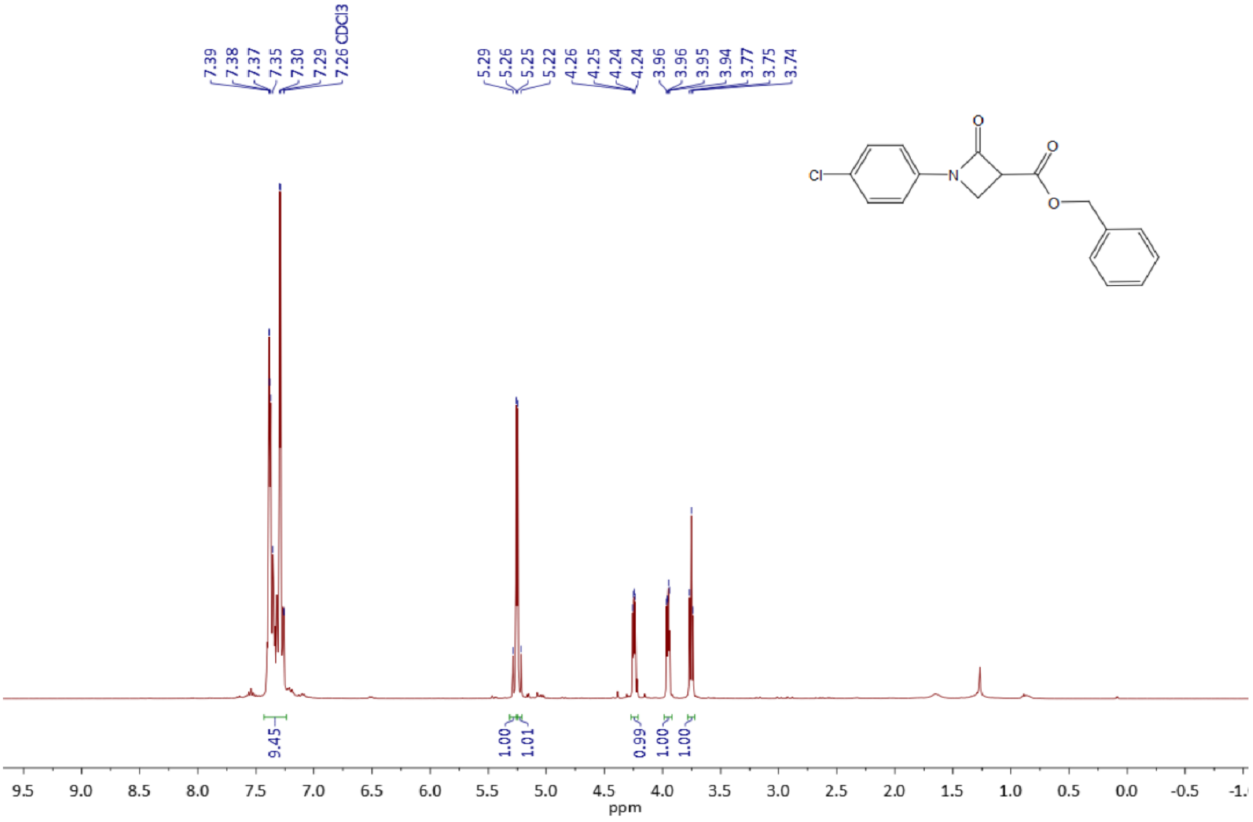


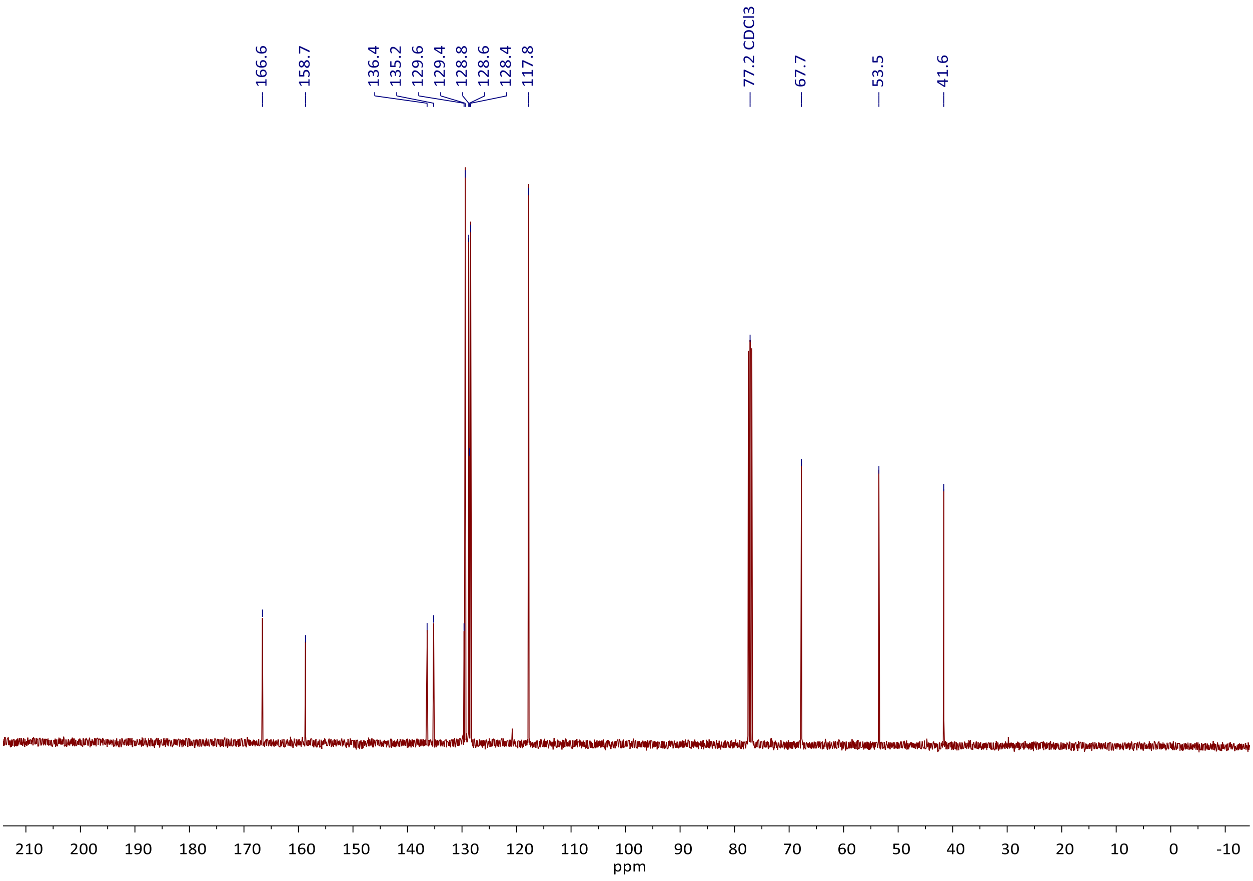

Copies of $^{1}H$ (400.13 MHz, $CDCl_3$) and $^{13}C\{^{1}H\}$ (100.61 MHz, $CDCl_3$) spectra of **3o**

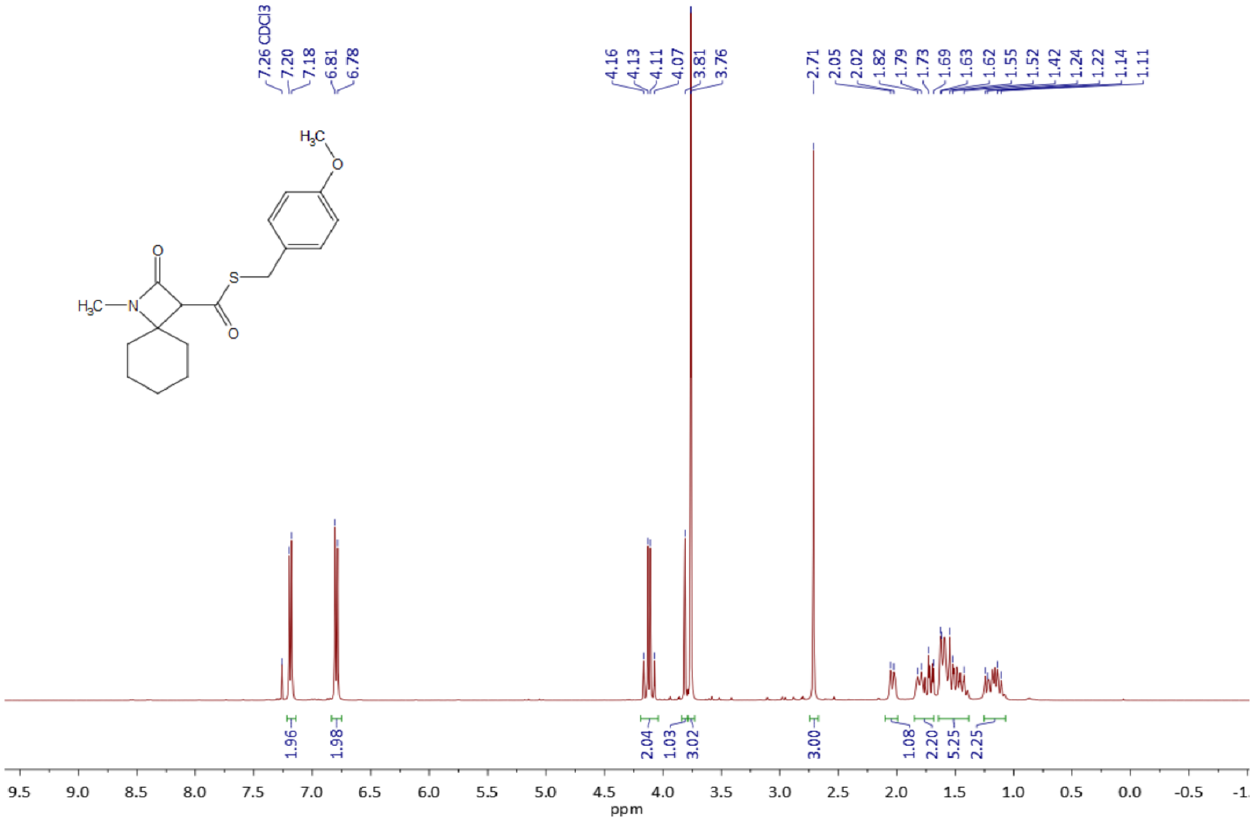

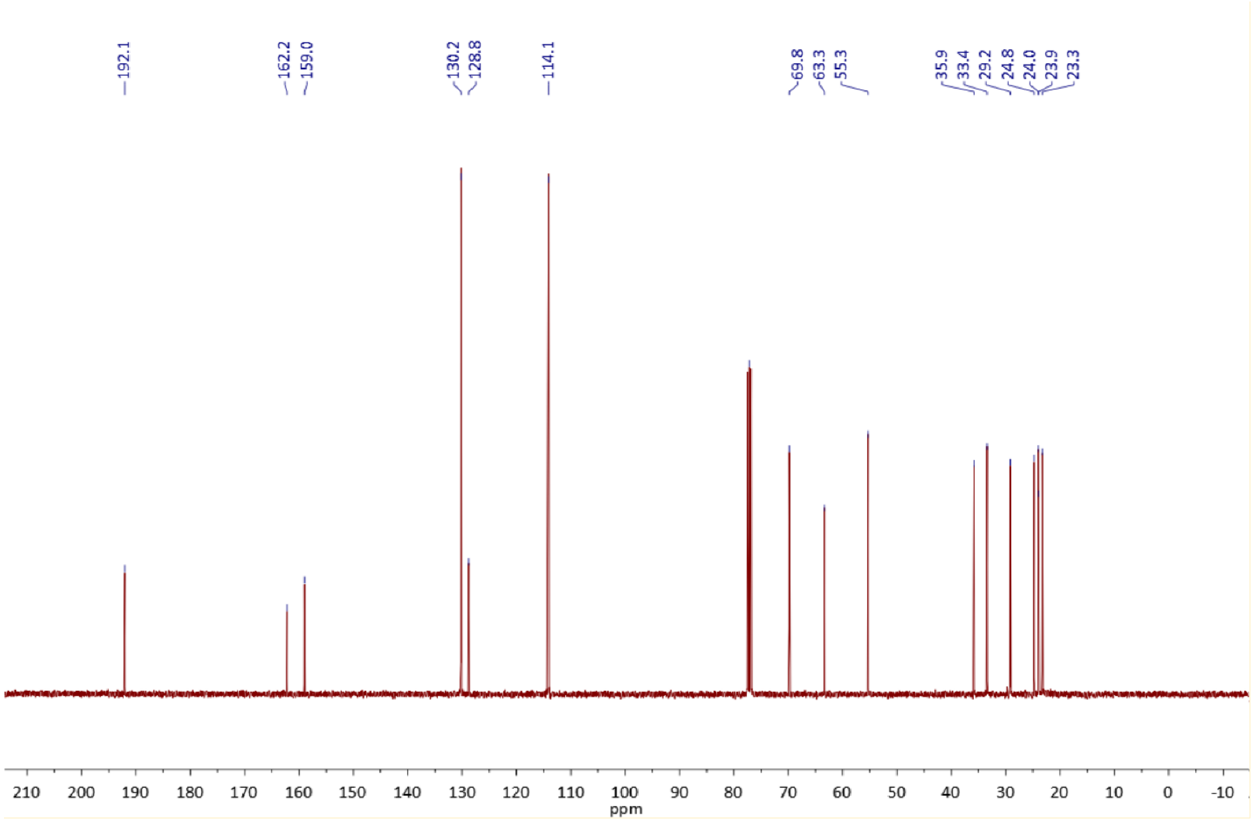

Copies of $^{1}H$ (400.13 MHz, $CDCl_3$) and $^{13}C\{^{1}H\}$ (100.61 MHz, $CDCl_3$) spectra of **3p**

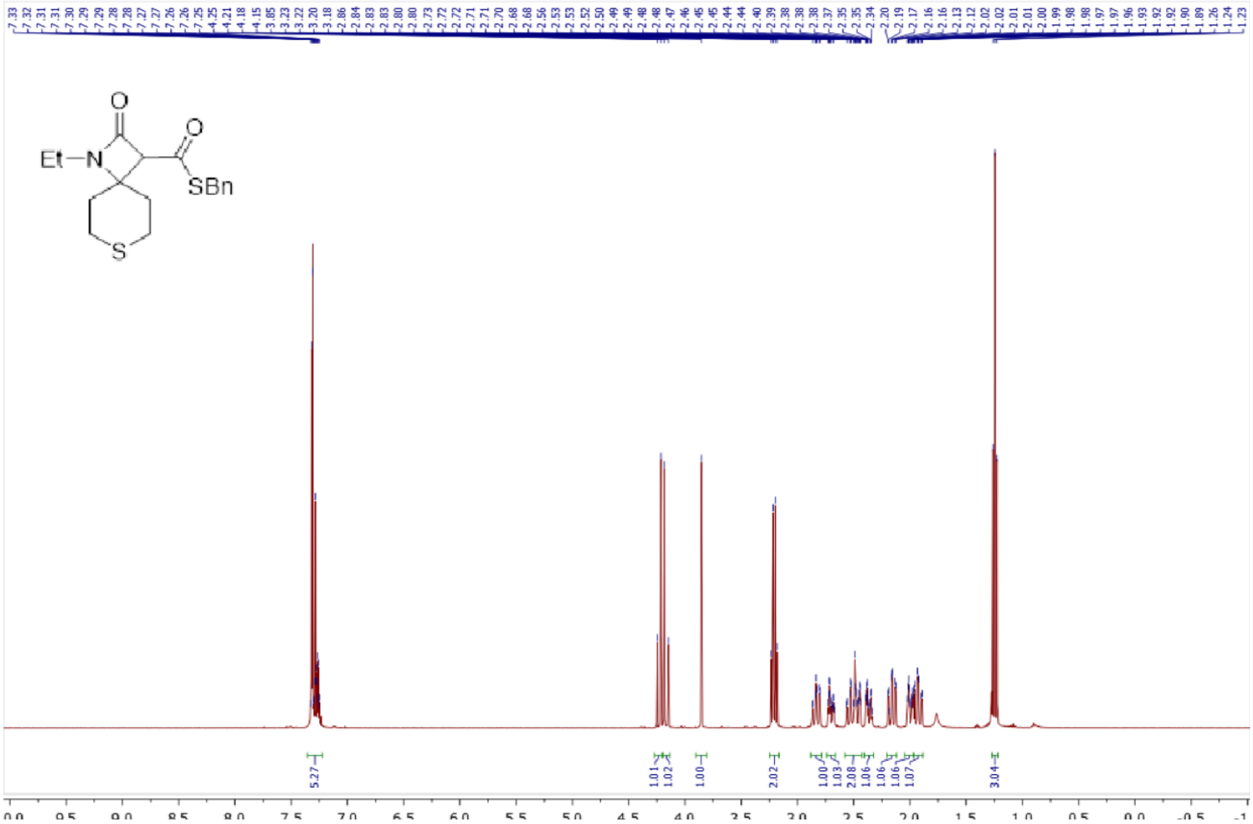

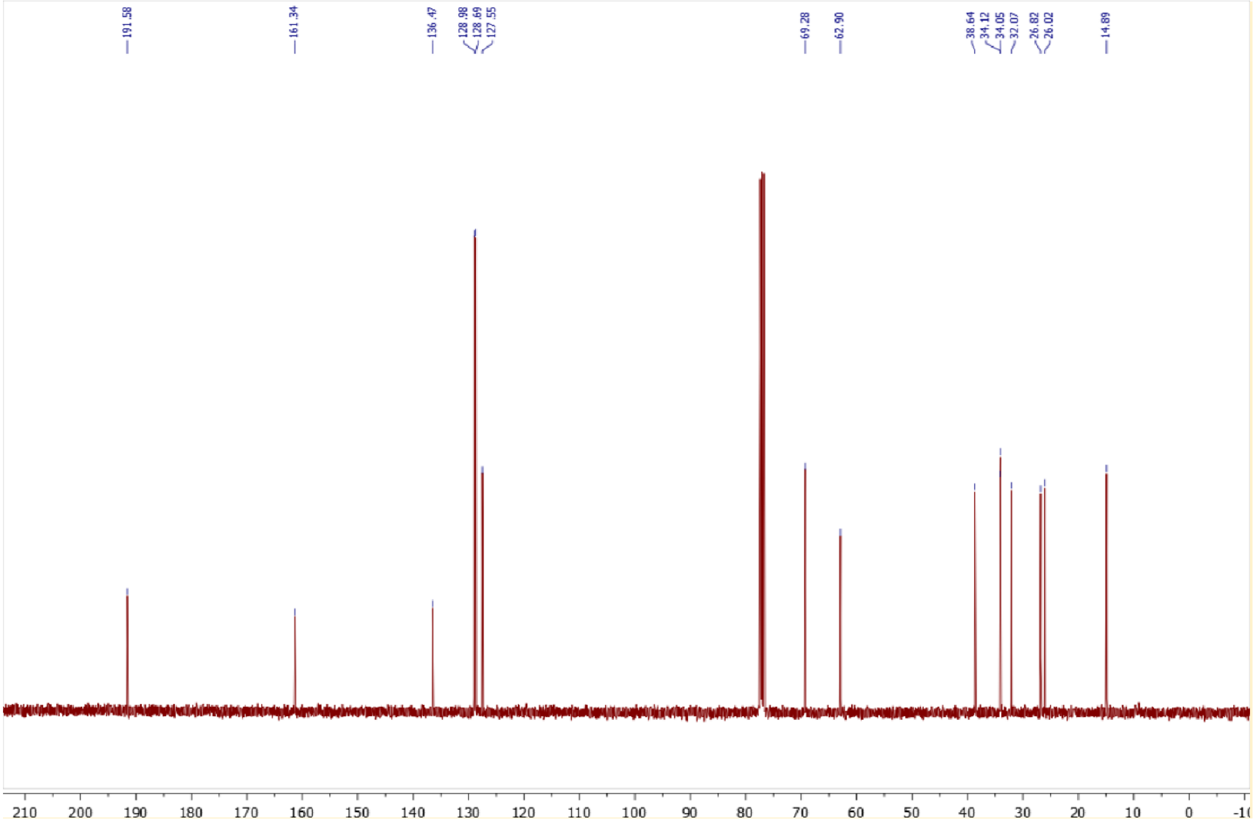

Copies of $^{1}H$ (400.13 MHz, $CDCl_3$) and $^{13}C\{^{1}H\}$ (100.61 MHz, $CDCl_3$) spectra of **3q**

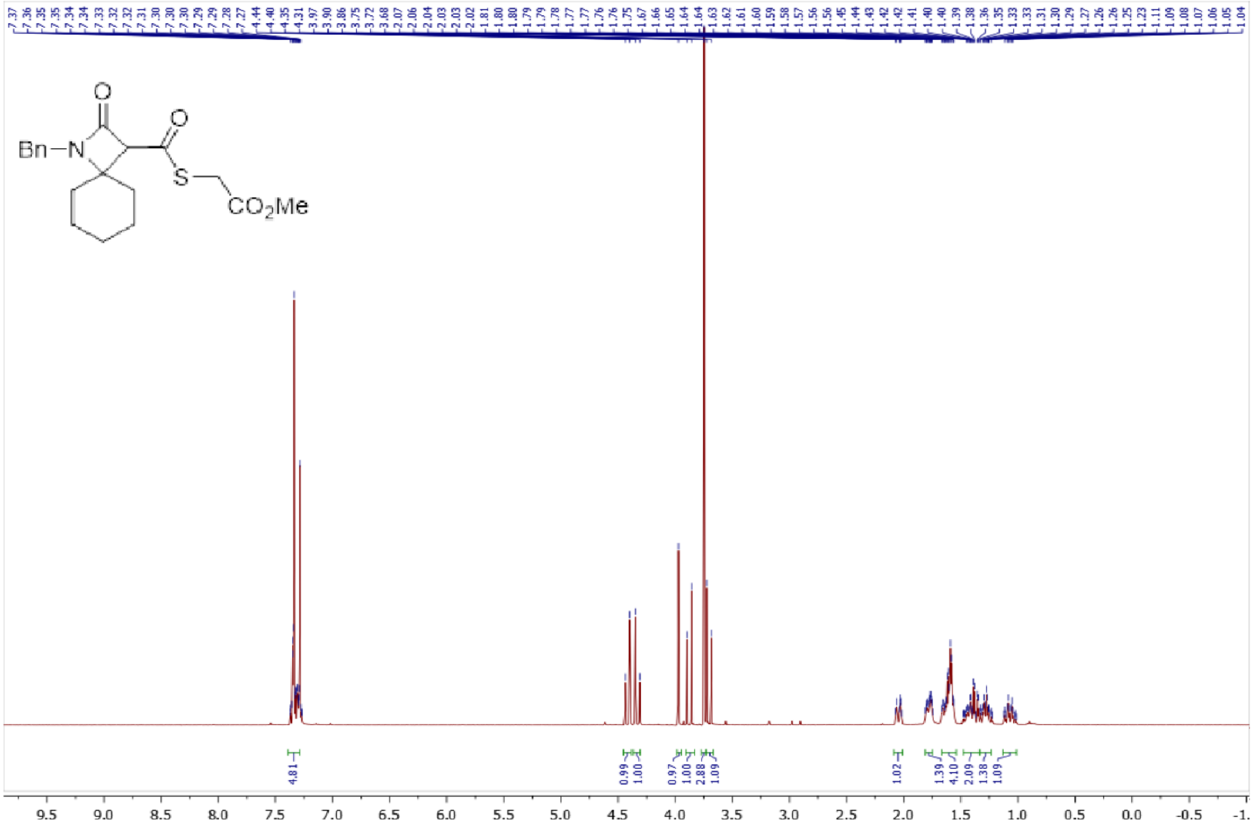


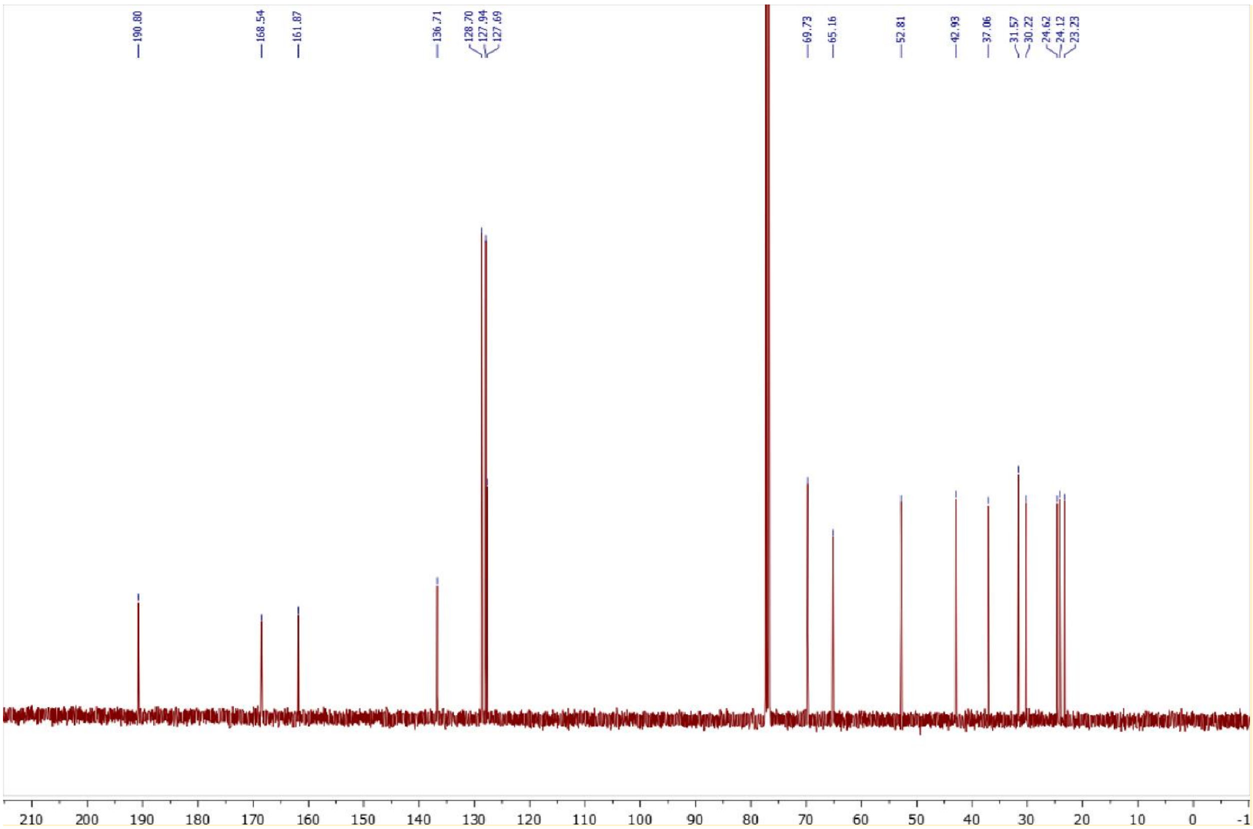

Copies of $^{1}H$ (400.13 MHz, $CDCl_3$) and $^{13}C\{^{1}H\}$ (100.61 MHz, $CDCl_3$) spectra of **3r**

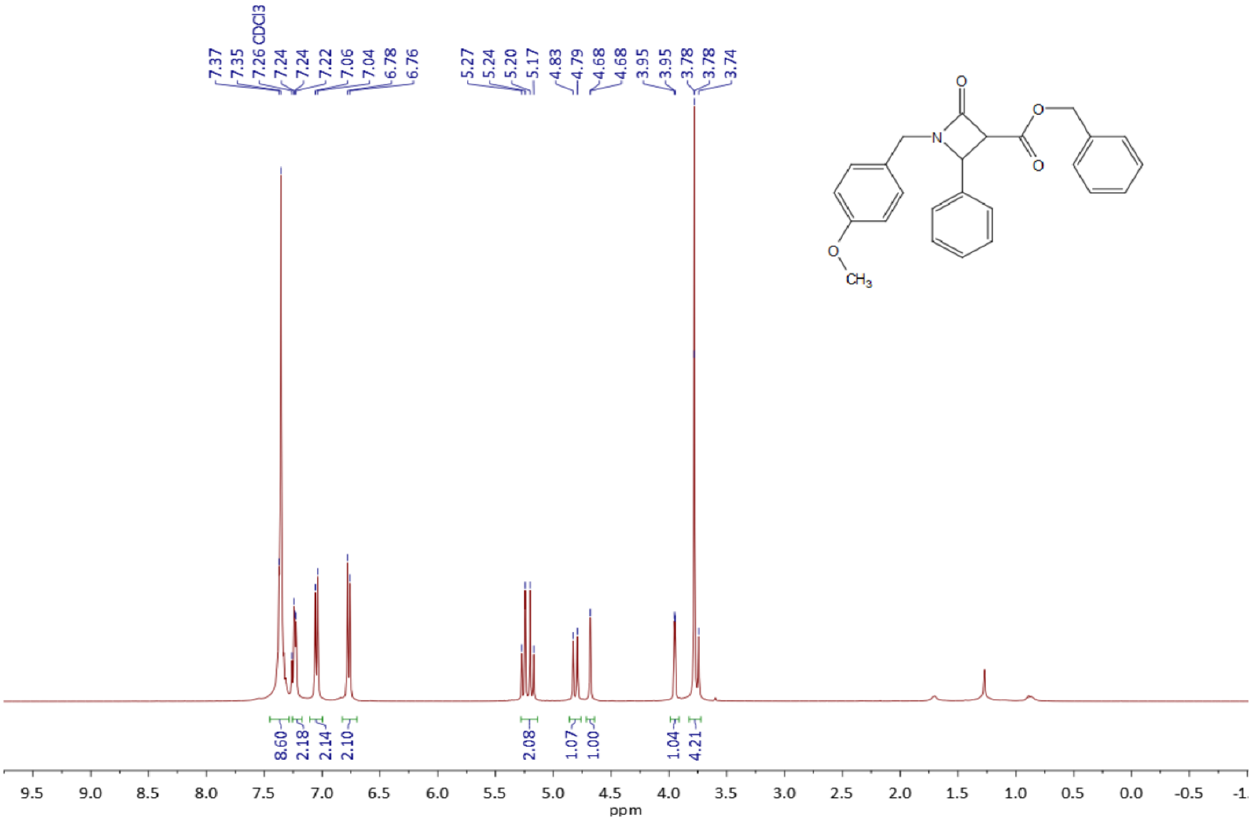


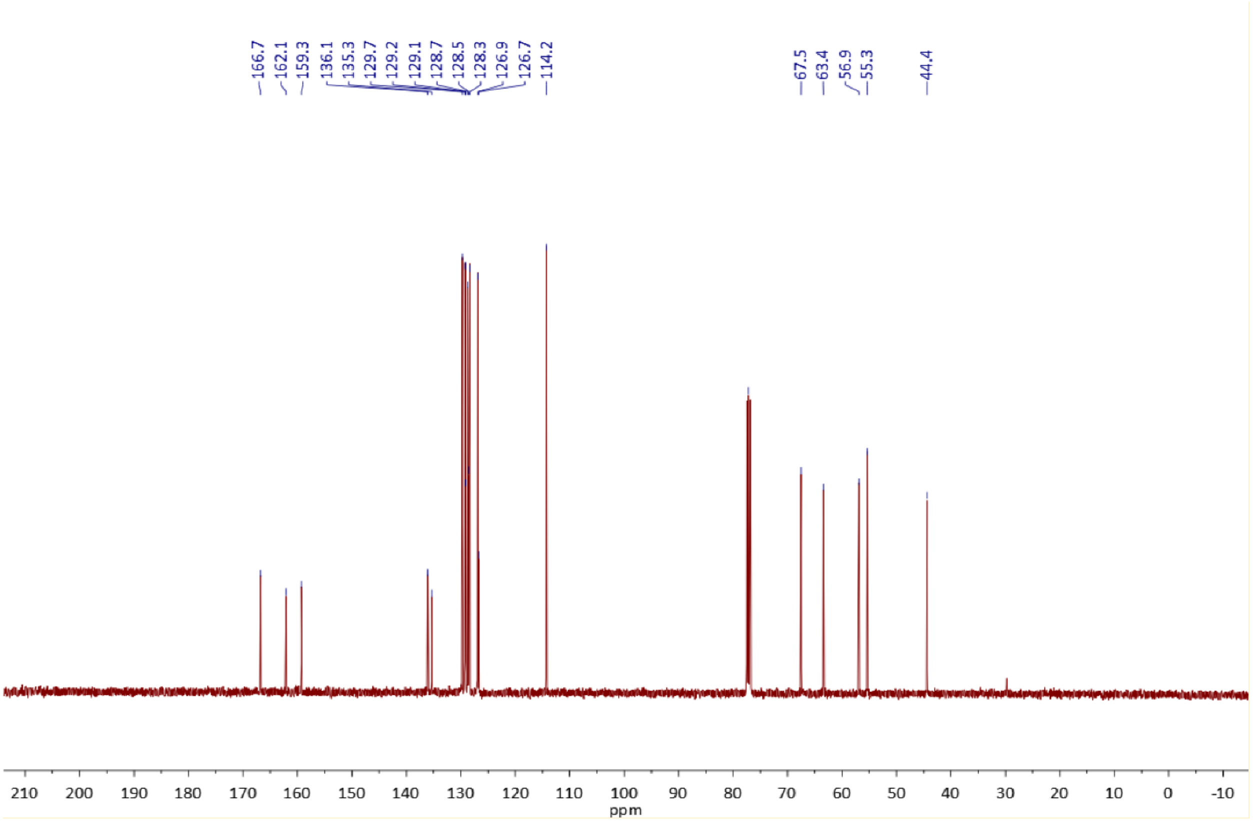

Copies of $^{1}H$ (400.13 MHz, $CDCl_3$) and $^{13}C\{^{1}H\}$ (100.61 MHz, $CDCl_3$) spectra of **3s**

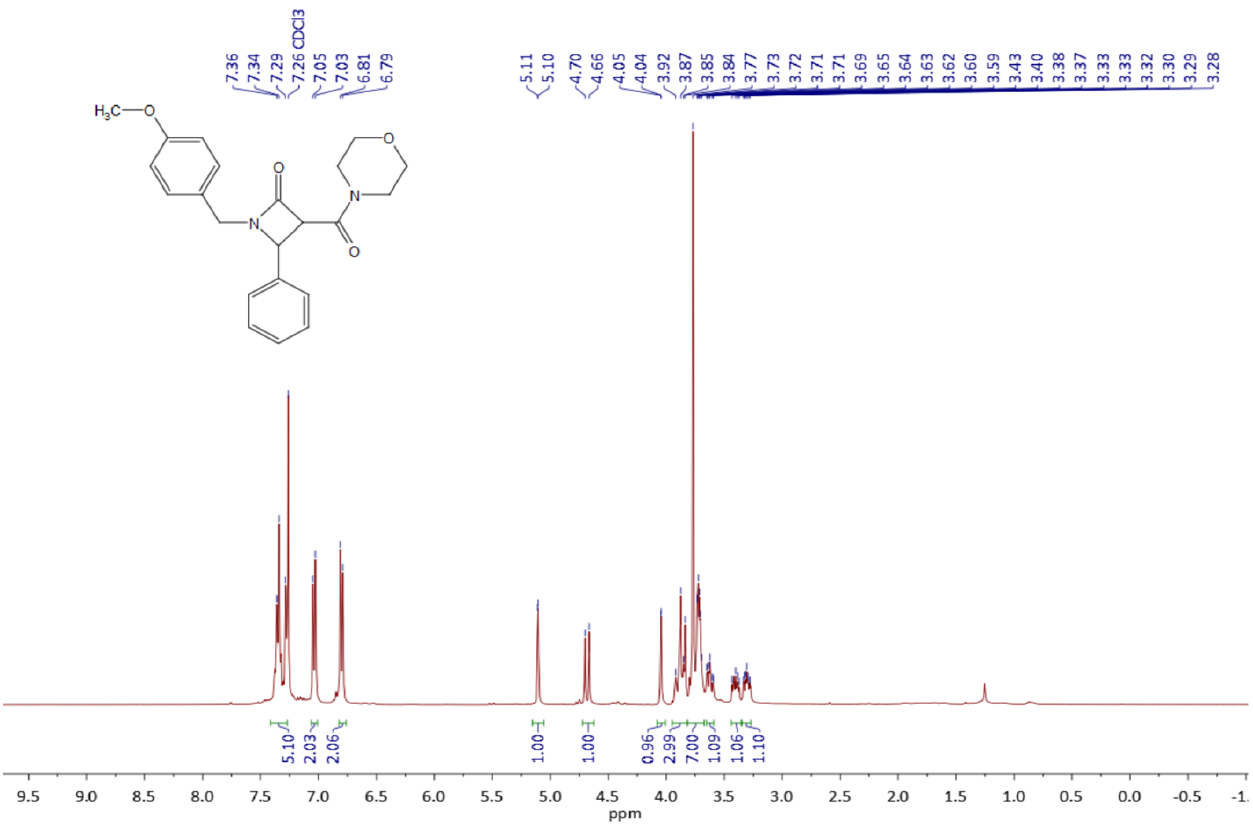


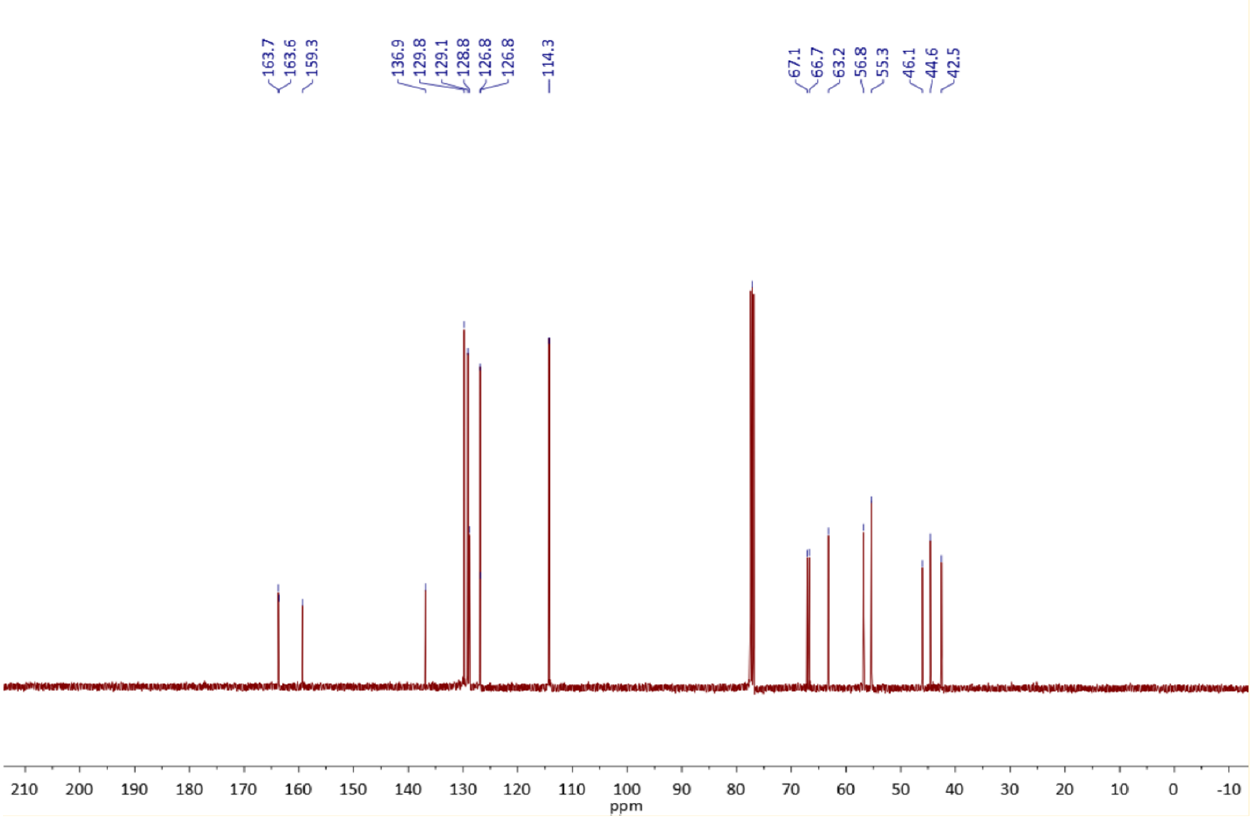

Copies of $^{1}H$ (400.13 MHz, $CDCl_3$), $^{13}C\{^{1}H\}$ (100.61 MHz, $CDCl_3$), and $^{19}F\{^{1}H\}$ (376.50 MHz, $CDCl_3$) spectra of **3t**

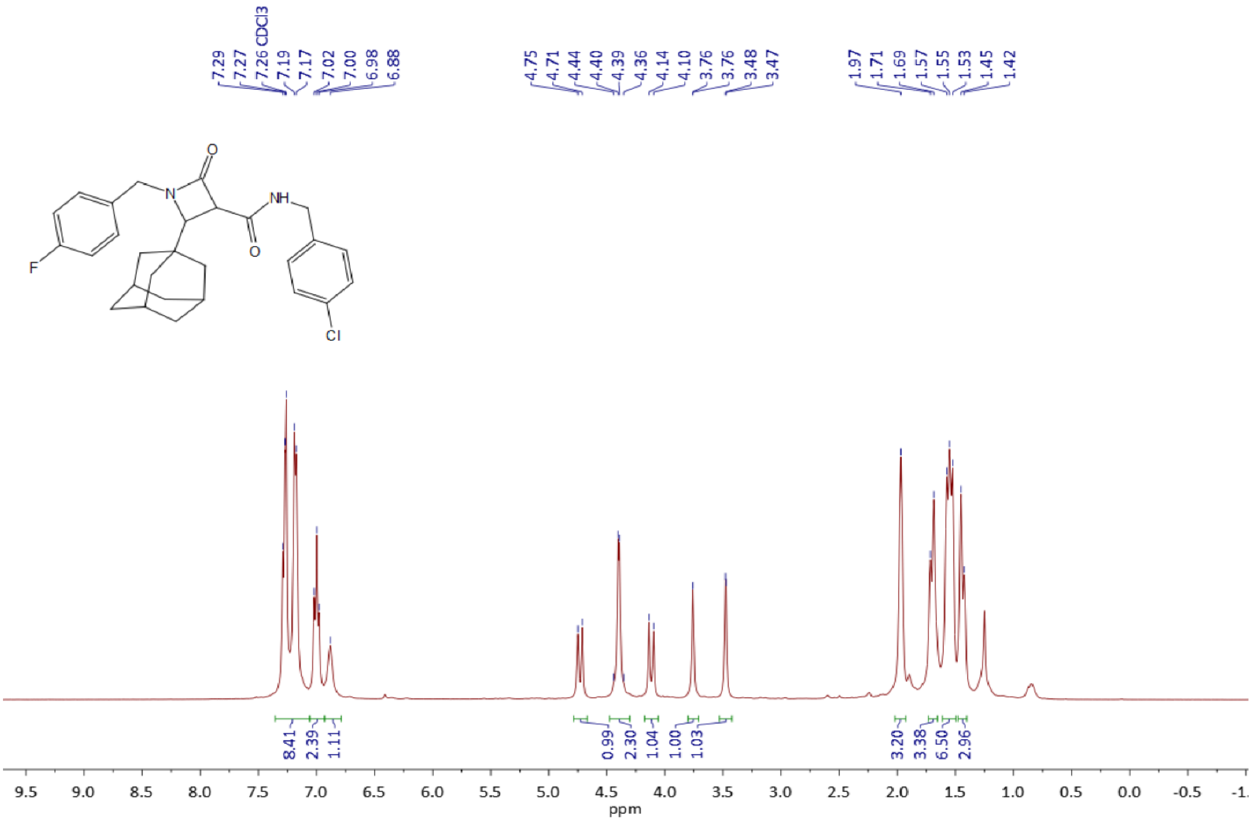


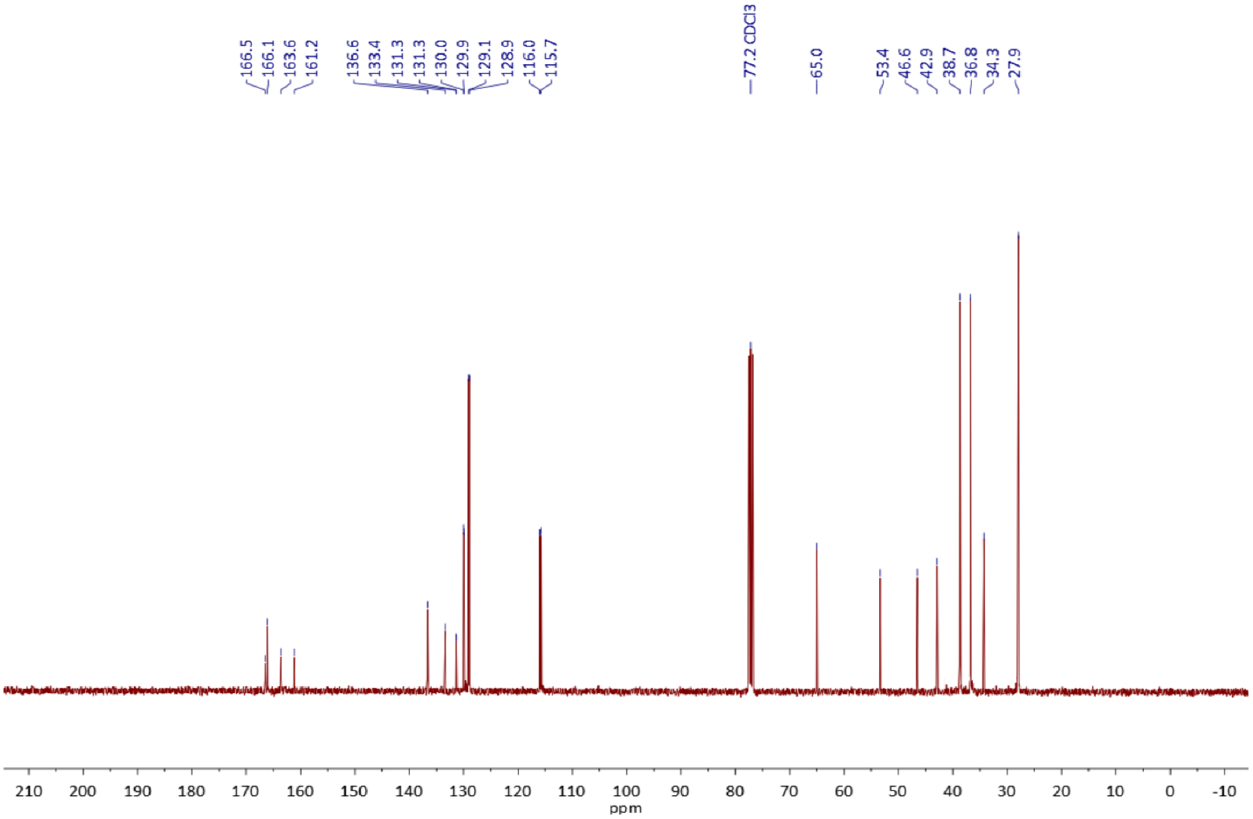

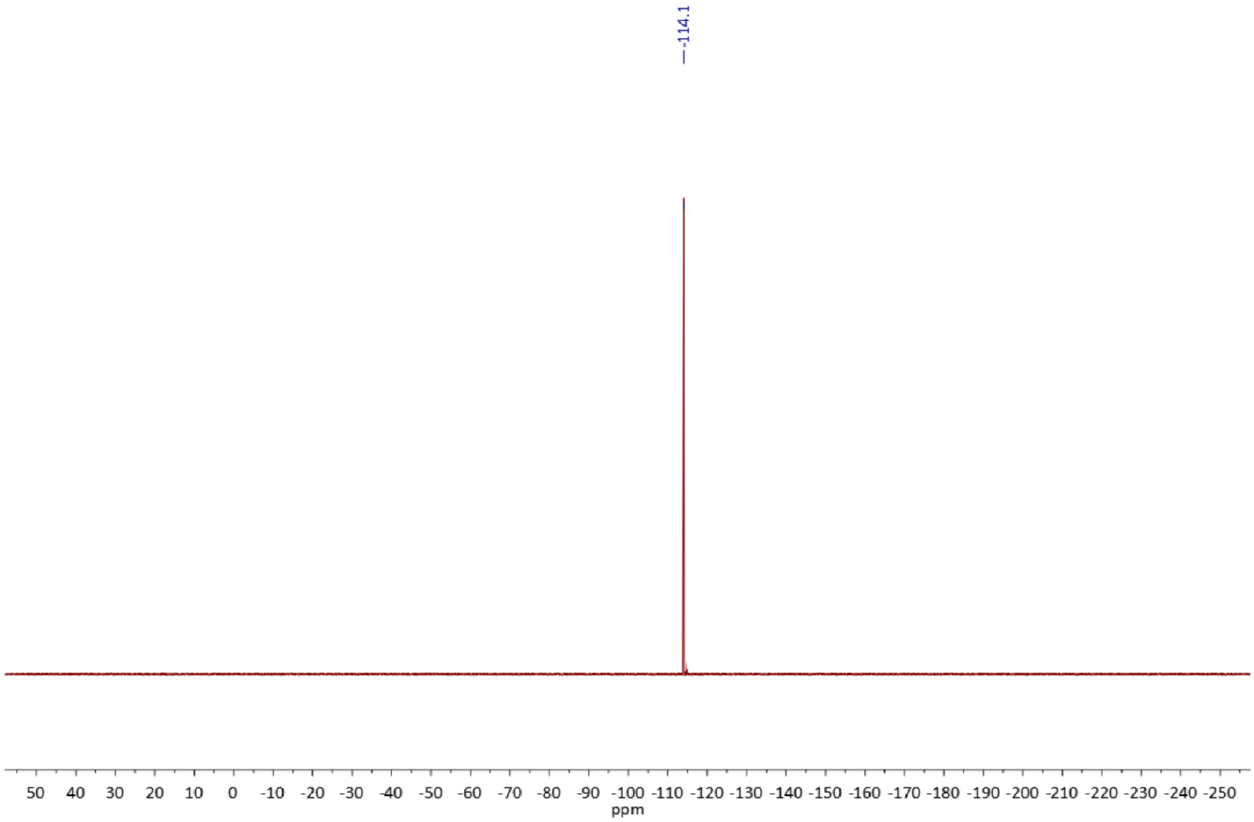
-114.1
50 40 30 20 10 0 -10 -20 -30 -40 -50 -60 -70 -80 -90 -100 -110 -120 -130 -140 -150 -160 -170 -180 -190 -200 -210 -220 -230 -240 -250
ppm

Copies of $^{1}H$ (400.13 MHz, $CDCl_3$) and $^{13}C\{^{1}H\}$ (100.61 MHz, $CDCl_3$) spectra of **3u**

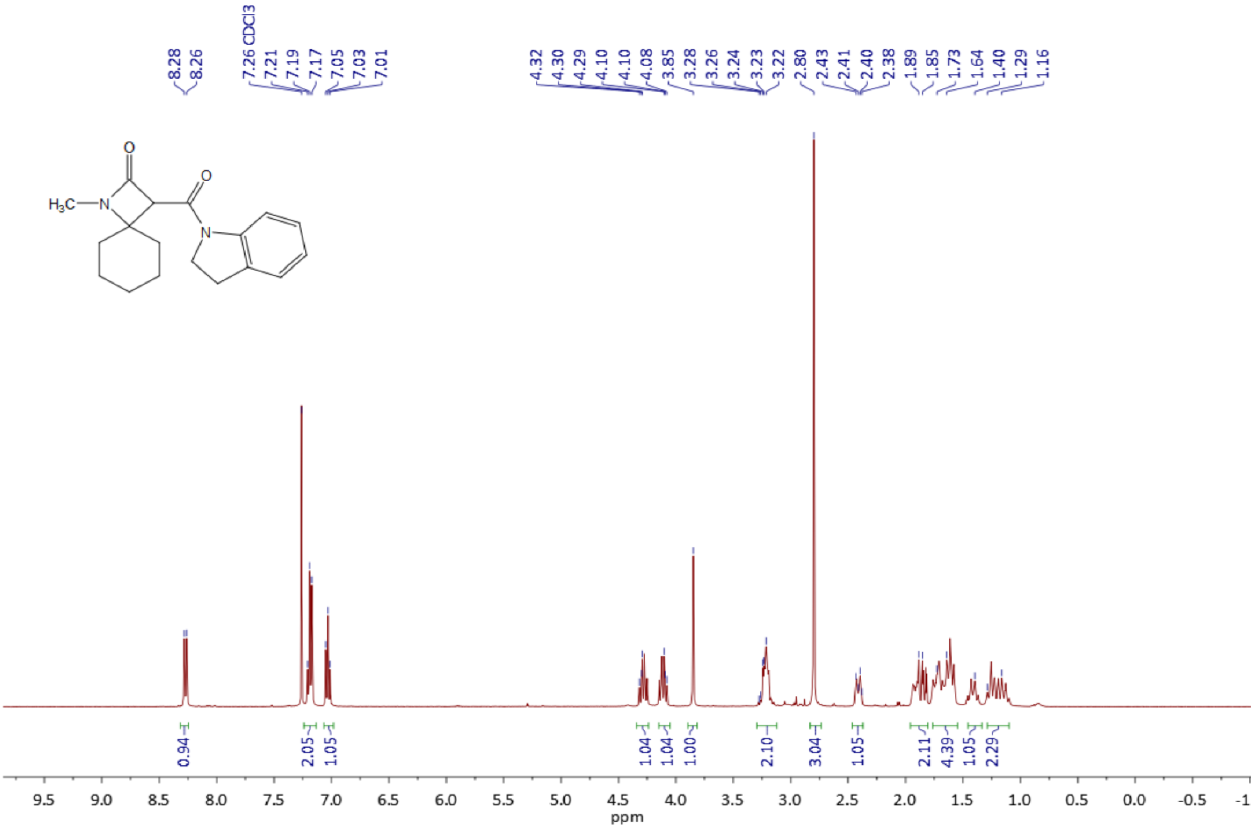


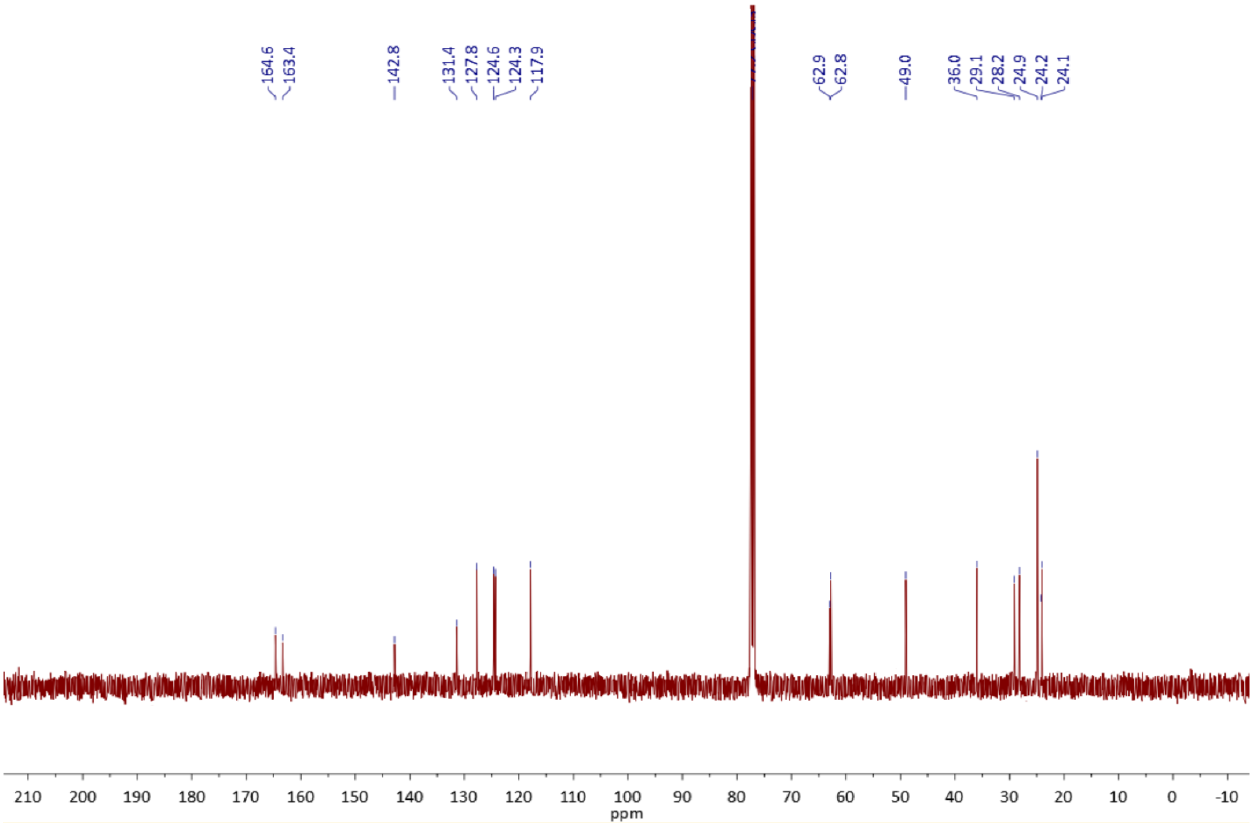

Copies of $^{1}$H (400.13 MHz, DMSO-$d_6$) and $^{13}$C{$^{1}$H} (100.61 MHz, DMSO-$d_6$) spectra of **3v**.

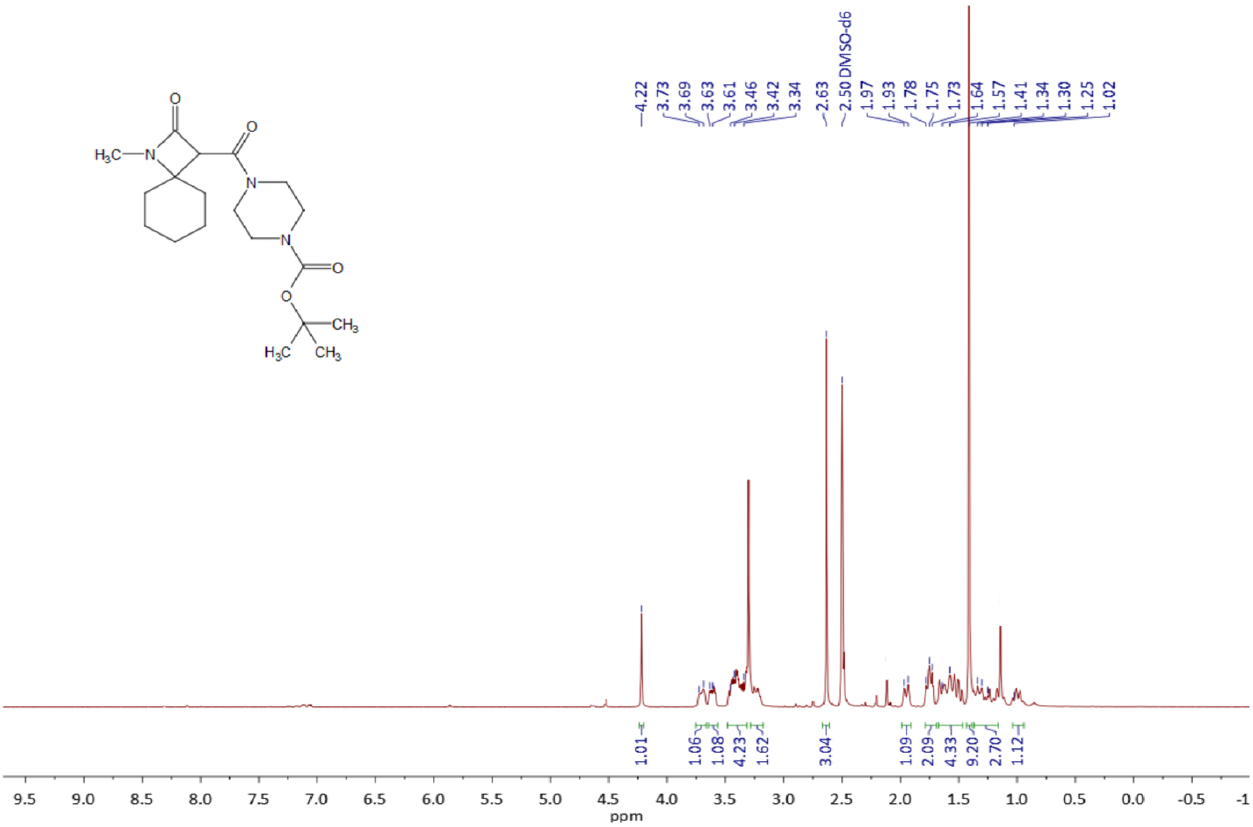


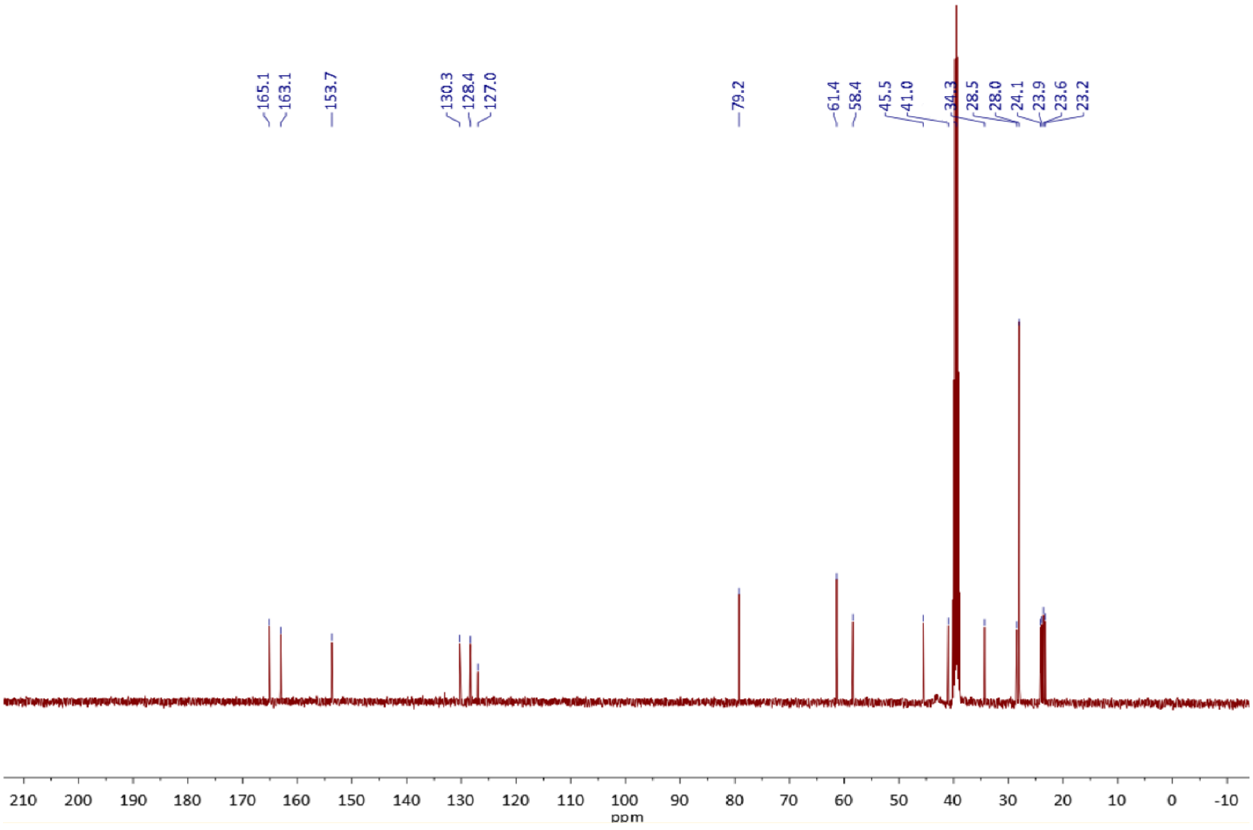

3.1. NMR spectra of β-lactamic acids **4a** and **4b**

Copies of $^{1}$H (400.13 MHz, CDCl3) and $^{13}$C{$^{1}$H} (100.61 MHz, CDCl3) spectra of **4a**

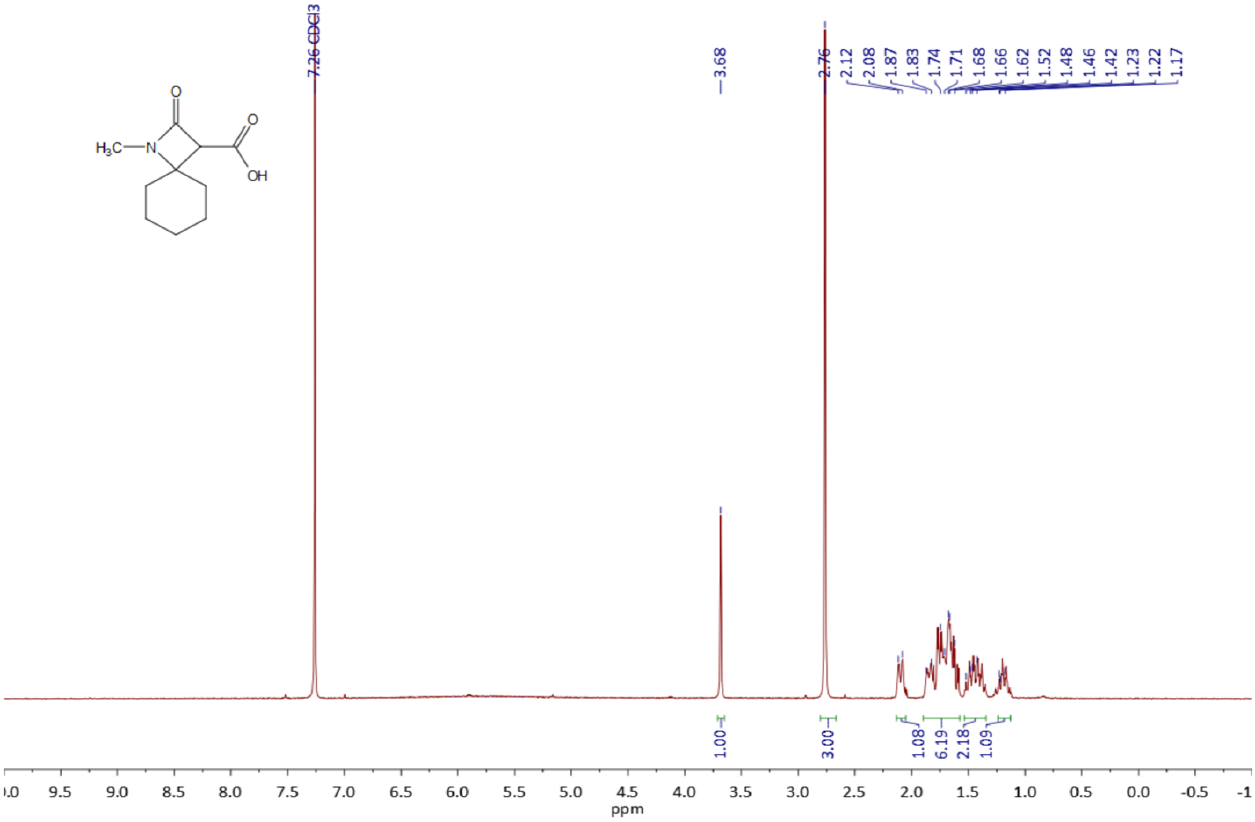


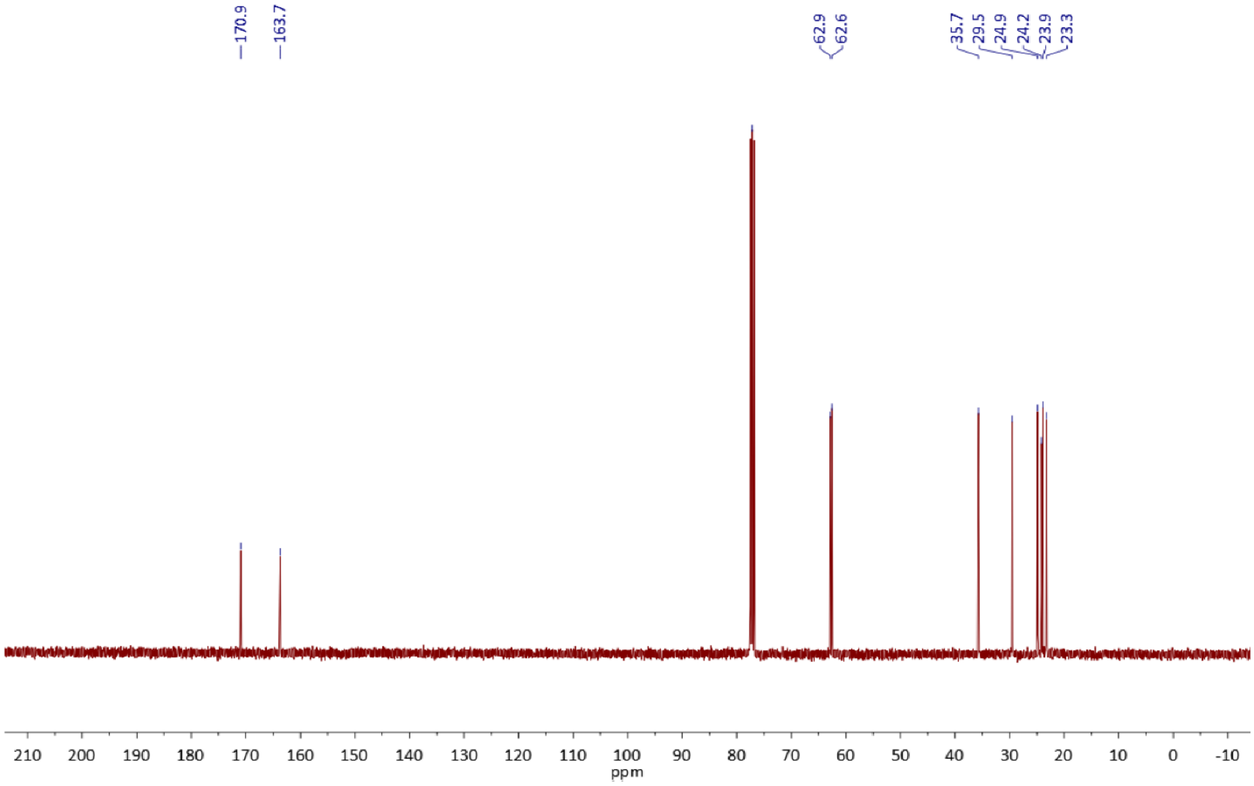

Copies of $^{1}H$ (400.13 MHz, $CDCl_3$) and $^{13}C\{^{1}H\}$ (100.61 MHz, $CDCl_3$) spectra of **4b**

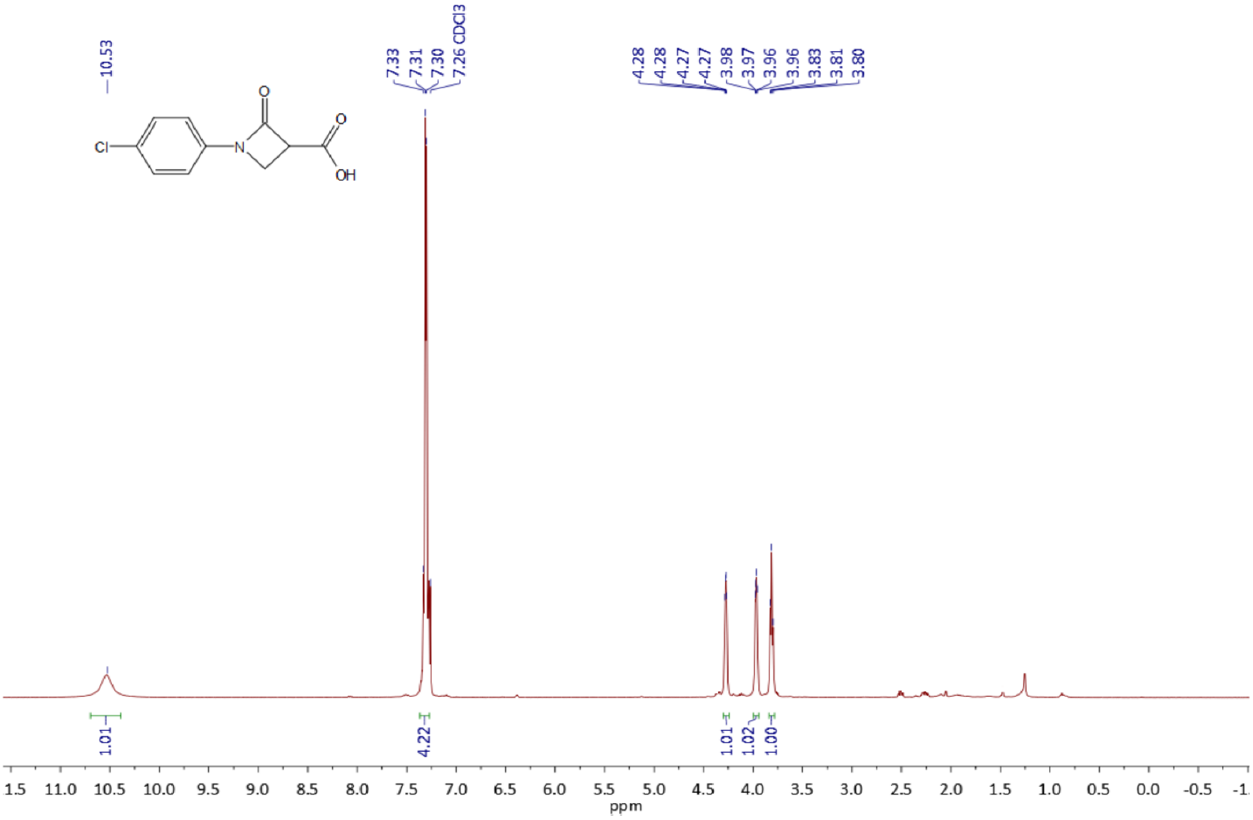


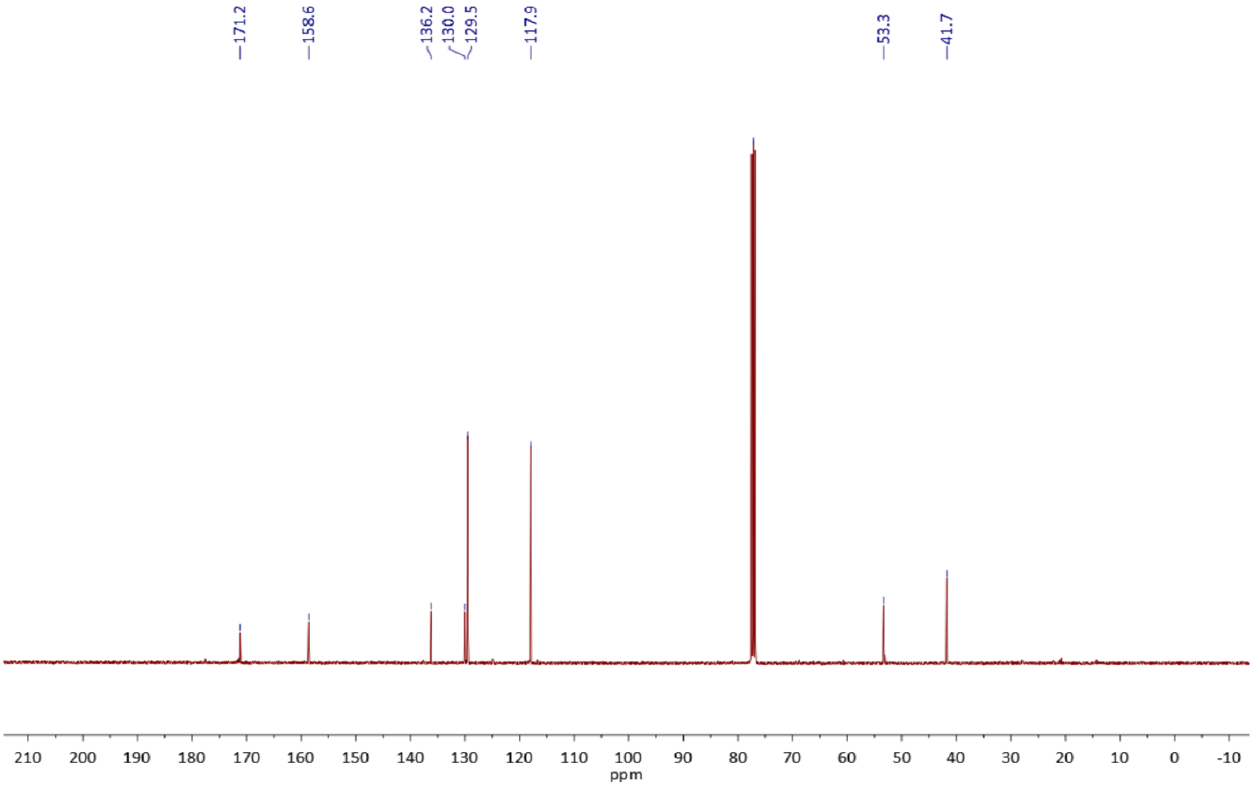

## 4. Crystal structure data for compound 3t

X-ray single crystal analysis was performed on a SuperNova diffractometer. Crystals were kept at 100(2)K during data collection. Using Olex2[2], the structures were solved with the SHELXT[3] structure solution program using Intrinsic Phasing and refined with the SHELXL[4] refinement package using Least Squares minimization.

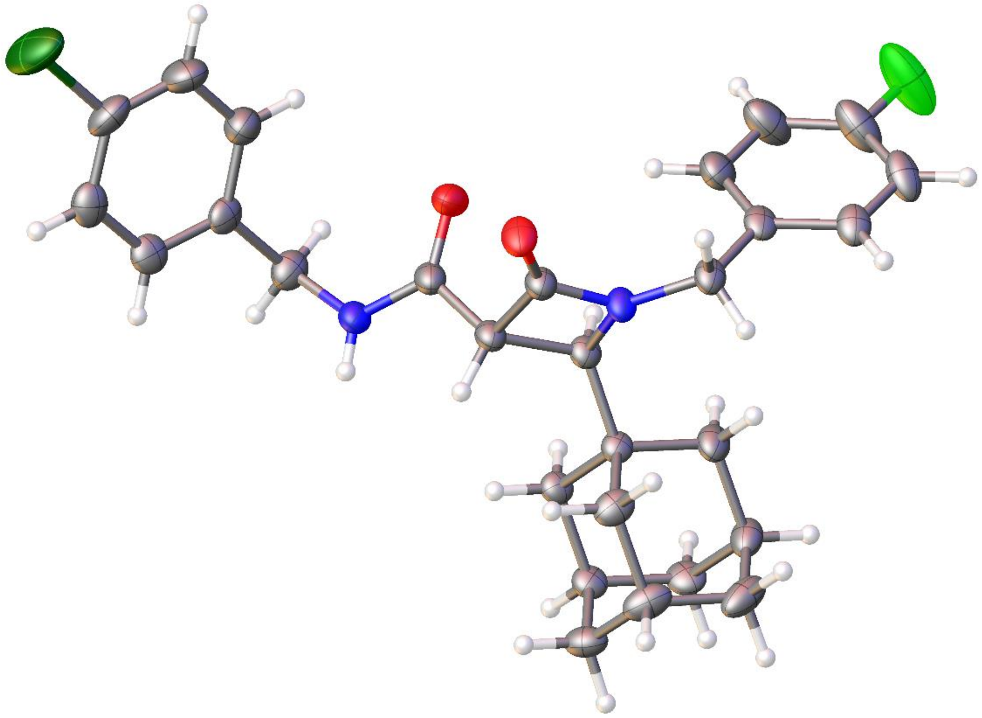

**Figure S1.** ORTEP representation of compound **3t** (thermal ellipsoids are shown at 50% probability).

**Table S1.** Crystal data and structure refinement for **3t**

| **CCDC** | **2323689** |
|---|---|
| Empirical formula | $C_{28}H_{30}ClFN_2O_2$ |
| Formula weight | 480.99 |
| Temperature/K | 100(2) |
| Crystal system | monoclinic |
| Space group | $P2_1/c$ |
| a/Å | 9.7326(3) |
| b/Å | 32.1486(5) |

| | |
|---|---|
| c/Å | 9.6486(2) |
| α/° | 90 |
| β/° | 118.040(4) |
| γ/° | 90 |
| Volume/Å$^3$ | 2664.58(14) |
| Z | 4 |
| $\rho_{calc}$g/cm$^3$ | 1.199 |
| μ/mm$^{-1}$ | 1.536 |
| F(000) | 1016.0 |
| Crystal size/mm$^3$ | 0.16 × 0.1 × 0.08 |
| Radiation | Cu Kα (λ = 1.54184) |
| 2Θ range for data collection/° | 5.498 to 134.966 |
| Index ranges | -7 ≤ h ≤ 11, -38 ≤ k ≤ 38, -11 ≤ l ≤ 11 |
| Reflections collected | 21040 |
| Independent reflections | 4646 [$R_{int}$ = 0.0718, $R_{sigma}$ = 0.0430] |
| Data/restraints/parameters | 4646/0/311 |
| Goodness-of-fit on F$^2$ | 1.055 |
| Final R indexes [I>=2σ (I)] | $R_1$ = 0.0690, w$R_2$ = 0.1838 |
| Final R indexes [all data] | $R_1$ = 0.0746, w$R_2$ = 0.1882 |
| Largest diff. peak/hole / e Å$^{-3}$ | 0.49/-0.75 |

## 5. References


(1) Dar'in, D.; Kantin, G.; Glushakova, D.; Sharoyko, V.; Krasavin, M. Diazo Tetramic Acids Provide Access to Natural-Like Spirocyclic $\Delta^{\alpha,\beta}$-Butenolides through Rh(II)-Catalyzed O–H Insertion/Base-Promoted Cyclization. *J Org Chem* **2023**, *89* (11), 7366–7375. https://doi.org/10.1021/acs.joc.2c02600.

(2) Dolomanov, O. V.; Bourhis, L. J.; Gildea, R. J.; Howard, J. A. K.; Puschmann, H. *OLEX2* : A Complete Structure Solution, Refinement and Analysis Program. *J Appl Crystallogr* **2009**, *42* (2), 339–341. https://doi.org/10.1107/S0021889808042726.

(3) Sheldrick, G. M. *SHELXT* – Integrated Space-Group and Crystal-Structure Determination. *Acta Crystallogr A Found Adv* **2015**, *71* (1), 3–8. https://doi.org/10.1107/S2053273314026370.

(4) Sheldrick, G. M. Crystal Structure Refinement with *SHELXL*. *Acta Crystallogr C Struct Chem* **2015**, *71* (1), 3–8. https://doi.org/10.1107/S2053229614024218.
